# The Effect of Chinese Medicine Compound in the Treatment of Rheumatoid Arthritis on the Level of Rheumatoid Factor and Anti-Cyclic Citrullinated Peptide Antibodies: A Systematic Review and Meta-Analysis

**DOI:** 10.3389/fphar.2021.686360

**Published:** 2021-06-30

**Authors:** Xuan Tang, Zehao Liu, Zhihua Yang, Shengmei Xu, Maojie Wang, Xiumin Chen, Zehuai Wen, Runyue Huang

**Affiliations:** ^1^ The Second Affiliated Hospital of Guangzhou University of Chinese Medicine (Guangdong Provincial Hospital of Chinese Medicine), Guangzhou, China; ^2^ Center for Molecular Medicine, University Medical Center Utrecht, Utrecht, Netherlands; ^3^ Guangdong Provincial Key Laboratory of Clinical Research on Traditional Chinese Medicine Syndrome, Guangzhou, China; ^4^ Guangdong-Hong Kong-Macau Joint Lab on Chinese Medicine and Immune Disease Research, Guangzhou University of Chinese Medicine, Guangzhou, China; ^5^ State Key Laboratory of Dampness Syndrome of Chinese Medicine, The Second Affiliated Hospital of Guangzhou University of Chinese Medicine, Guangzhou, China

**Keywords:** Chinese medicine compound, rheumatoid arthritis, rheumatoid factor, anti-cyclic citrullinated peptide antibodies, meta-analysis

## Abstract

**Objectives:** To evaluate the current evidence whether Chinese medicine compound (CMC) can reduce the serum levels of rheumatoid factor (RF) and anti-cyclic citrullinated peptide antibodies (anti-CCP).

**Methods:** We comprehensively searched PubMed, Embase, the Cochrane Library, China National Knowledge Infrastructure (CNKI), the Database for Chinese Technical Periodicals (VIP), and Wanfang data. We then performed a systematic review and meta-analysis of all randomized controlled trials (RCTs) assessing the CMC therapy methods. This study is registered with PROSPERO, number CRD42020216284.

**Results:** In total, 65 studies were eligible for inclusion, including 6099 patients. The result of the meta-analysis showed that compared with common Western medicine therapy, CMC monotherapy or combined with Western medicine was able to reduce serum RF (SMD= −0.85, 95%CI −1.04 to −0.67) and anti-CCP (SMD= −0.56, 95%CI −0.79 to −0.32) levels to some extent. In the efficacy meta-analysis, a greater number of CMC-treated patients achieved the efficacy criteria after a period of treatment, where the relative risk (RR) was 1.20 [1.08, 1.33] for achieving ACR20, 1.57 [1.38, 1.78] for ACR50, and 2.21 [1.72, 2.84] for ACR70. At the same time, there was a statistically significant difference in the effective rate of the patient's TCM symptoms (RR = 1.22, 95%CI 1.19–1.26).

**Conclusions:** Through this meta-analysis and systematic review, we found that CMC for the treatment of RA is effective in reducing RF and anti-CCP levels and might have better clinical efficacy than Western medicine monotherapy. Some active components are responsible for this efficacy and worth further exploring.

## Highlights

1. Through this meta-analysis and systematic review, we found that CMC to treat RA is effective in reducing RF and anti-CCP levels and might have better clinical efficacy than Western medicine monotherapy.
2. Through frequency analysis of the CMC constituent herbs involved in the 65 literatures included in the meta-analysis, we summarized the active ingredients and pharmacological effects of five high-frequency representative Chinese herbs and found that CMC can reduce RF and anti-CCP levels possibly by regulating the immune response and inhibiting B lymphocyte proliferation.
3. CMC may be a potential and efficacious therapeutic adjunct to delay the progression and improve outcomes of RA.

## Introduction

As an incurable autoimmune disease, rheumatoid arthritis (RA) can cause cartilage and bone damages as well as disability, and finally lead to poor quality of life. The average prevalence of RA is estimated at 0.5–1.0% globally (Ngian, 1999). In China, the people suffer from RA with an estimated prevalence of 0.42% (Zeng et al., 2013). Although remission is now an achievable goal for the majority of patients owing to advances in early diagnosis, new drugs and improved treatment strategies, the rate of reaching the standard of domestic RA treatment in China is still low at present. Currently, mainstream therapeutic strategy, i.e. Western medical treatment, including disease-modifying anti-rheumatic drugs (DMARDs), biologic agents, glucocorticoids (GC), which are considered to be effective means to rapidly alleviate and control the progression of RA, but the toxic side effects caused by Western medicine long-term use (Rainsford, 1999; Wang et al., 2018), the non-response of some patients to drugs (He et al., 2019), and the expensive price of biologic agents are the main problems faced by Western medical treatment. Therefore, there is still a considerable unmet need in RA treatment, which has led to an increasing number of patients with RA to seek complementary and integrative medicines.

Traditional Chinese Medicine (TCM), the most common complementary and alternative therapeutic approach for Western medicine, also has a long history in the treatment of RA, whether taken internally or externally, monotherapy or in combination, it has obvious therapeutic effects with few side effects. Chinese medicine compound (CMC) is the main form and means of clinical use of TCM, which concentrates the advantages and characteristics of TCM in the treatment of diseases. Under the guidance of the theoretical system that the concept of holism and treatment based on syndrome differentiation, CMC is formulated as a mixture of different kinds of Chinese herbal medicines, including decoction, granule and Chinese patent medicine, etc. In China, it is quite common that DMARDs are combined with various CMC in the treatment of RA. Modern scholars of TCM have carried out many clinical studies on CMC for the treatment of RA, but the literature reported in these clinical studies is of varying quality and results. One study (Yao, 2010) showed that the efficacy of the CMC experimental group was better than that of the control group after two months of treatment, and the difference was statistically significant (*p* < 0.05). On the contrary, the results of Chen's randomized controlled trial (Chen et al., 2010) found that the total effective rate in the CMC group was lower than that in the Western medicine group. Especially the efficacy in alleviating pain symptoms was inferior to that of the Western medicine group, and the difference was statistically significant (*p* < 0.05). It is precisely because of the differences in conclusions between studies that we need to conduct a systematic review to objectively evaluate the role and underlying mechanisms of CMC in the treatment of RA.

RF and anti-CCP are serological indicators for the diagnosis of RA. Anti-CCP has higher specificity and sensitivity for RA than RF, which combined detection with RF can compensate for the lack of specificity and sensitivity of RF, and has good diagnostic value for RA (van Venrooij et al., 2008). There are several studies showing that anti-CCP is a sensitive serological indicator of the degree of bone erosion and predict the prognosis of RA patients, thus assisting in the optimal therapeutic management of RA patients (Forslind et al., 2004; Ronnelid et al., 2005; Schoels et al., 2011). So far, no systematic review has been found to describe the efficacy of CMC in reducing RF and CCP levels in RA patients.

The following is a systematic review and meta-analysis of randomized controlled trials (RCTs) of CMC in the treatment of RA, to provide some references for improving RA therapeutic strategy.

## Methods

### Literature Search and Strategy

According to the Preferred Reporting Items for Systematic Reviews and Meta-analyses (PRISMA), we searched the PubMed, embase, Cochrane Library, China National Knowledge Infrastructure (CNKI), the database for Chinese Technical Periodicals (VIP) and Wanfang data from the inception dates to September 31, 2020. The keywords used were as follows: Chinese keywords were Chinese pinyin such as “Zhongyi, Zhongyao and Zhongyiyao” (which means “Traditional Chinese Medicine”) and “Leifengshiguanjieyan, Leifengshixingguanjieyan” (which means “rheumatoid arthritis”). while English searches combined subject terms (MeSH) and free words, with a retrieval strategy of “Arthritis, Rheumatoid” or “rheumatoid arthritis” AND “Medicine, Chinese Traditional” or “Chinese medicine” or “herbal medicine” or “Traditional Chinese Medicine”.

### Study Selection Criteria

#### Study Type

We merely included the RCTs that involved CMC to treat RA, regardless of blinding, publication status or language.

#### Participant Type

Adults (usually over 18 years of age) with a diagnosis of RA either using the 1987 American College of Rheumatology (ACR) classification criteria (Arnett et al., 1988) for RA, or using the 2010 ACR/European League Against Rheumatism (EULAR) classification criteria (Aletaha et al., 2010) for RA, and regardless of gender, age, the severity of disease, duration of disease, etc.

#### Intervention Measures

All experimental groups were administered orally with any types of CMC, including CMC monotherapy or combined with Western medicine. The control groups received only oral Western medicine treatment.

#### Major Research Indicators

##### Primary Outcomes

The primary outcomes include mean serum RF and anti-CCP levels after CMC treatment.

##### Secondary Outcomes

The secondary outcomes pertained to the clinical efficacy.

1) The efficacy of response of RA to treatment with CMC by the ACR outcome measure ACR20, 50 and 70. The ACR20, 50 and 70 response is defined as at least a 20, 50 and 70% reduction from baseline in the number of both tender and swollen joints and at least a 20, 50 and 70% improvement in three or more of the five remaining ACR core set measures (patient’s assessment of pain, level of disability, C-reactive protein level, global assessment of disease by the patient, and global assessment of disease by the physician) (Felson et al., 1995).
2) Standard of curative effect of TCM symptoms. Refer to the “Guiding Principles for Clinical Research of New Chinese Medicines”: 1) Effective: decrease of integral symptoms scored ≥30%, some relief of TCM symptoms; 2) Ineffective: integral symptoms score lower <30%, no relief or even aggravation of TCM symptoms (Zheng, 2002).

#### Exclusion Criteria

Exclusion criteria included republished literature; the literature whose research topic is complications of RA; animal experiments, reviews, conference papers, incomplete case reports or important data reports with no reply from the corresponding author(s).

### Data Selection

Three authors participated in the data extraction of all the studies included in the review. Two authors (Xuan Tang and Zehao Liu) independently extracted the relevant data from the eligible studies. A third author (Zhihua Yang) resolved any divergence still present after discussion. Data extracted from the selected studies included authors' name, publication year, trial design, characteristics of participants, intervention methods, components of CMC, duration of treatment and endpoint evaluation indicators.

### Quality Assessment

Assessment of risk of bias was undertaken for each included study using the Cochrane Collaboration’s risk of bias assessment tool (Green and Higgins, 2008). Seven sources of bias were assessed: random sequence generation, allocation concealment, blinding of participants and personnel, blinding of outcome assessment, incomplete outcome data, selective outcome reporting, and other sources of bias. The evaluation criteria of each item were judged as “low risk of bias”, “unclear risk of bias” and “high risk of bias”.

### Statistical Analysis

Extracted data were combined for meta-analysis using R4.0.3 and Stata12.0 software. The dichotomous data was evaluated using the relative risk (RR) and 95% confidence interval (CI), and the continuous data were combined using the standardized mean difference (SMD) and 95% CI. The *I*-squared [*I*(Zeng et al., 2013)] statistic was used to assess the heterogeneity across the included studies, as suggested by literature (Higgins et al., 2003). The analysis was carried out using a fixed-effects model according to if *I*-squared ≤50%. Instead, a random-effects model was adopted when significant heterogeneity (*I*-squared > 50%) was found. In addition, Egger's test was used to estimate and represent the risk of potential publication bias if the number of included trials reached 10 (Begg and Mazumdar, 1994).

At the same time, we performed descriptive statistics on the frequency of each component of CMC involved in the 65 literature that was finally included in the meta-analysis, in order to explore the high-frequency Chinese medicine for RA treatment and their active ingredients and pharmacological effects, and to find out the potential mechanism that reduces serum RF and anti-CCP levels.

### Subgroup Analysis and Investigation of Heterogeneity

Where sufficient studies were available and the data were heterogeneous, we carried out separate meta-analyses for studies according to some factors including intervention duration and intervention measures of the experimental group.

### Sensitivity Analysis

We performed a sensitivity analysis to explore heterogeneity and the differences in effect size. After excluding different studies in turn and re-performing the meta-analysis of the remaining studies, we assessed whether the results obtained were significantly different from those before the exclusion so as to assess whether the results of the meta-analysis were robust.

## Results

### Literature Search Results

**FIGURE 1:**
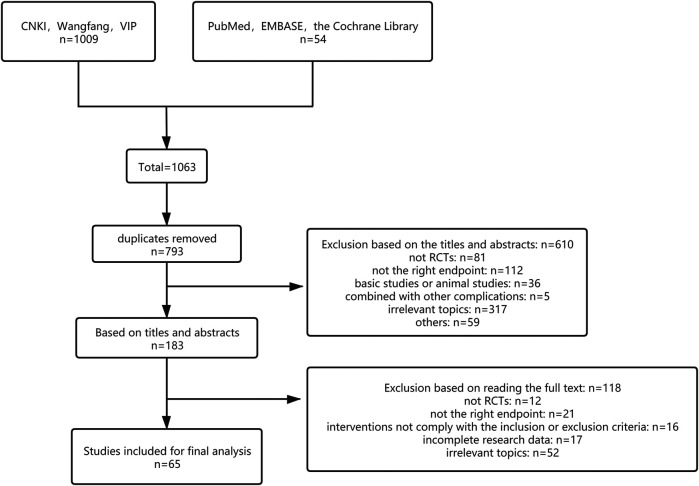
Flow diagraph of study selection.

According to the retrieval strategy, 1,063 literature was initially detected, including 471 of CNKI, After removal of duplicates via literature management software across databases, 793 studies were screened. Through reading titles and abstracts, 610 literature was excluded because they did not meet the inclusion criteria. And 118 literature was excluded after examination of the full text. Finally, 65 studies that met the inclusion criteria were included in our meta-analysis. Figure 1 shows the process and consequences of literature screening. The process followed for the selection of eligible studies is described in a flow diagram (Figure 1).

### Characteristics of Studies

A total of 65 studies were included in the meta-analysis were conducted in China. These studies, published between 2006 to 2020, included 6,131 patients, 3139 RA patients that received CMC, and 2992 RA patients that received Western medicine monotherapy. In the end, 6,099 patients completed the study. A total of 63 studies (Huang et al., 2006; Li, 2006; Wang et al., 2006; Liu et al., 2007; Luo, 2008; Shen et al., 2008; Li, 2009; Liu et al., 2009; Ma et al., 2009; Xiang et al., 2009; Chen et al., 2010; Yao, 2010; Zhou et al., 2010; Han and Song, 2011; Shen et al., 2011; Yang et al., 2011; Li et al., 2012a; Li, 2013; Liu et al., 2013; Wei et al., 2013; Wei and Liu, 2013; Yang, 2013; Cao and Liu, 2014; He and Xiao, 2014; Niu, 2014; Wang and Tao, 2014; Yu and Chen, 2014; Zhang and Chen, 2014; Li et al., 2015a; Shu et al., 2015; Su et al., 2015; Zhao, 2015; Zheng and Shi, 2015; Chen et al., 2016; Han et al., 2016; Jia et al., 2016; Jiang and Wu, 2016; Liu et al., 2016; Qian et al., 2016; You et al., 2016; Gao and Bu, 2017; Guo et al., 2017; Li et al., 2017; Pang et al., 2017; Wang, 2017; Wang and Tu, 2017; Bian and Wang, 2018; Ge, 2018; Guo et al., 2018; Hu, 2018; Ma et al., 2018; Shi and Yang, 2018; Xu et al., 2018; Zeng and Chen, 2018; Fang et al., 2019; Li et al., 2019; Pang et al., 2019; Song et al., 2019; Yuan et al., 2019; Zhang, 2019; Zhao, 2019; Cao et al., 2020; Jiang and Ma, 2020) and 8 studies (Liu et al., 2009; Chen et al., 2010; Han et al., 2016; Gao and Bu, 2017; Pang et al., 2017; Bian and Wang, 2018; Pang et al., 2019; Li, 2020) explored RF and anti-CCP levels respectively after treatment. In addition, 14 studies (Chen et al., 2010; Shen et al., 2011; Yang, 2013; Cao and Liu, 2014; He and Xiao, 2014; Wang and Tao, 2014; Zhang and Chen, 2014; Li et al., 2015b; Shu et al., 2015; Zhao, 2015; Jiang and Wu, 2016; Qian et al., 2016; Wang and Tu, 2017; Pang et al., 2019; Cao et al., 2020) researched the efficacy of response of ACR20, 50 and 70. Moreover, 33 studies (Huang et al., 2006; Li, 2006; Luo, 2008; Shen et al., 2008; Liu et al., 2009; Xiang et al., 2009; Yao, 2010; Zhou et al., 2010; Liu et al., 2013; Wei et al., 2013; Wei and Liu, 2013; Yang, 2013; He and Xiao, 2014; Li et al., 2015a; Shu et al., 2015; Zheng and Shi, 2015; Chen et al., 2016; Han et al., 2016; Jiang and Wu, 2016; Liu et al., 2016; You et al., 2016; Guo et al., 2017; Li et al., 2017; Wang and Tu, 2017; Bian and Wang, 2018; Ge, 2018; Guo et al., 2018; Ma et al., 2018; Shi and Yang, 2018; Xu et al., 2018; Li et al., 2019; Song et al., 2019; Yuan et al., 2019) reported the effective rate of TCM symptoms.

The experimental group received oral CMC or CMC combined with Western medicine as intervention measures, while the control group received conventional Western medicine for the treatment of RA as the positive control. Among all the medications included in the study, CMC included decoction, patent medicines (including capsules, tablets, and pills), and powder. Western medicine mainly included DMARDs such as methotrexate (MTX), leflunomide (LEF), sulfasalazine (SSZ), hydroxychloroquine sulfate (HCQ), as well as nonsteroidal anti-inflammatory drugs (NSAIDs) and GC, etc. There were no statistically significant differences between the basic information about the patients. A summary with baseline characteristics of included patients is shown in Table 1. The details (including dosages, herbs, quality control and mentions) of interventions in above studies were disposed in Supplementary Material 1 and Table 2. All herbs used in are assessable and well documented in Supplementary Material 5 (including their Pinyin Names, Accepted Names, Latin Names, Full Formats and Medicinal Sources).

**TABLE 1:** Characteristics of the included studies.

Author	Year	Sample size		Disease stage		Age (Y)		Intervention methods		Intervention duration	Outcomes
		EG	CG	EG	CG	EG	CG	EG	CG		
Li, X.	2015	36	36	—	—	37.5 ± 11.9	38.6 ± 12.7	MTX + NSAIDs + CMC	MTX + NSAIDs	12W	①③
Li, Y.	2015	29	30	—	—	49.03 ± 13.8	47.98 ± 12.92	CMC	LEF	12W	①④
Cao, Y. H.	2014	31	31	5.6 ± 3.3Y	5.8 ± 3.1Y	42.2 ± 8.6	41.3 ± 9.0	LEF + NSAIDs + CMC	LEF + NSAIDs	12W	①③
Li, J. H.	2019	40	39	6.64 ± 2.65Y	6.18 ± 2.53Y	59.43 ± 9.69	58.36 ± 9.15	MTX + NSAIDs + CMC	MTX + NSAIDs	12W	①④
Bian, Z. Q.	2018	84	79	6.58 ± 2.24Y	5.89 ± 2.01Y	48.58 ± 14.36	50.66 ± 13.57	NASIDs + CMC	NASIDs	12W	①②④
Yuan, L.	2019	15	15	6–12 M	4–12M	30–50	25–50	MTX + NSAIDs + CMC	MTX + NSAIDs	12W	①④
Yu, M.	2014	32	32	5.8 ± 3.6Y	6.1 ± 3.9Y	38.2 ± 6.8	38.5 ± 7.2	MTX + SSZ + NSAIDs + CMC	MTX + SSZ + NSAIDs	12W	①
Shen, Y. P.	2008	30	10	4.65 ± 2.47Y	4.01 ± 2.55Y	35.32 ± 14.43	34.98 ± 14.67	GC + NSAIDs + CMC	GC + NSAIDs	12W	①④
Niu, J. H.	2014	31	30	0.5–40Y	0.5–42Y	14–68	13–65	MTX + CMC	MTX	14W	①
Zhang, H. J.	2014	30	30	3.6 ± 1.7Y	3.9 ± 2.1Y	36.8 ± 10.3	37.8 ± 10.7	NASIDs + CMC	NASIDs	12W	①③
Xiang, C. C.	2009	40	40	4.5 ± 1.5Y	4.9 ± 1.3Y	50.24 ± 7.25	51.12 ± 6.85	MTX + SSZ + NSAIDs + CMC	MTX + SSZ + NSAIDs	12W	①④
Luo, Y. H.	2008	32	28	1–16Y	1–15Y	60–75	62–74	CMC	MTX + SSZ + NSAIDs	12W	①④
Shi, L.	2018	40	38	7.33 ± 4.67Y	7.45 ± 4.30Y	51.47 ± 8.36	53.24 ± 8.65	NASIDs + CMC	NASIDs	8W	①④
Ge, S.	2018	46	46	4.6 ± 0.7Y	4.5 ± 0.8Y	44.8 ± 4.8	44.5 ± 5.3	MTX + NSAIDs + CMC	MTX + NSAIDs	12W	①④
Liu, Y.	2016	35	30	5.97 ± 2.88Y	6.03 ± 2.70Y	46.89 ± 9.15	45.40 ± 8.63	MTX + LEF + CMC	MTX + LEF	4W	①④
Song, W. H.	2019	66	66	6.68 ± 0.84Y	6.73 ± 0.88M	53.27 ± 6.56	52.89 ± 6.41	MTX + CMC	MTX	12W	①④
Guo, H. M.	2017	340	300	5.3 ± 4.5Y	4.9 ± 4.7M	37.4 ± 8.5	36.5 ± 7.8	CMC + NSAIDs	MTX + NSAIDs	12W	①④
Hu, H.	2018	43	43	23.64 ± 3.05M	24.82 ± 4.08M	43.26 ± 4.82	44.05 ± 4.71	MTX + CMC	MTX	8W	①
Li, J.P.	2013	40	40	41.52 ± 5.62M	42.82 ± 2.69M	72.38 ± 6.16	72.32 ± 6.38	LEF + CMC	LEF	12W	①
Wang, S. M.	2006	40	20	2.8 ± 1.4Y	2.6 ± 1.3Y	41.4 ± 11.6	40.2 ± 12.9	MTX + SSZ + NSAIDs + CMC	MTX + SSZ + NSAIDs	36W	①
Jiang, D. et al.	2016	44	44	5.5 ± 1.6Y	5.3 ± 1.4Y	47.3 ± 11.2	48.2 ± 10.1	LEF + CMC	LEF	24W	①③④
Fang, X.G. et al.	2019	57	57	3.03 ± 0.36Y	3.02 ± 0.38Y	46.68 ± 7.59	45.59 ± 7.82	LEF + NSAIDs + CMC	LEF + NSAIDs	12W	①
Mang, Z. L. et al.	2018	25	25	11.60 ± 1.16Y	10.80 ± 1.08Y	49.68 ± 2.47	46.88 ± 2.11	MTX + NSAIDs + CMC	MTX + NSAIDs	6W	①④
Zhao, F. C.	2015	55	55	24.15 ± 12.03M	22.35 ± 14.32M	46.32 ± 12.31	48.34 ± 10.65	MTX + GC + CMC	MTX + GC	9W	①③
He, D. C. et al.	2014	30	30	169.38 ± 25.8D	155.47 ± 24.94D	49.69 ± 11.84	50.16 ± 12.14	MTX + LEF + CMC	MTX + LEF	12W	①③④
Ma, W. K. et al.	2009	34	34	4.71 ± 4.64Y	4.42 ± 4.92Y	48.35 ± 13.24	47.74 ± 14.92	MTX + SSZ + NSAIDs + CMC	MTX + SSZ + NSAIDs	24W	①
Jia, F. Y. et al.	2016	62	58	87 ± 50.97M	81.52 ± 56.27M	49.18 ± 11.40	49.10 ± 13.06	LEF + NSAIDs + CMC	LEF + NSAIDs	8W	①
Li, D.	2020	55	55	4.76 ± 1.56Y	4.21 ± 1.63Y	54.06 ± 8.89	53.56 ± 10.06	MTX + SSZ + NSAIDs + CMC	MTX + SSZ + NSAIDs	12W	②
Jiang, P. et al.	2020	96	90	8.67 ± 3.23Y	8.48 ± 3.40Y	45.41 ± 4.87	45.65 ± 4.90	MTX + SSZ + NSAIDs + CMC	MTX + SSZ + NSAIDs	4W	①
Han, L. et al.	2016	40	36	7.36 ± 6.23Y	7.46 ± 6.35Y	67.53 ± 7.24	68.14 ± 7.72	LEF + GC + CMC	LEF + GC	12W	①②④
Huang, G. D. et al.	2006	47	47	7.8 ± 3.2Y	7.9 ± 3.5Y	38 ± 7	38 ± 7	MTX + D-PEN + CMC	MTX + D-PEN	24W	①④
Zhang, K. L.	2019	82	78	4.3 ± 1.0Y	4.2 ± 1.1Y	54.9 ± 4.9	55.9 ± 5.4	MTX + LEF + CMC	MTX + LEF	4W	①
Li, A. M.	2009	67	67	68 ± 47M	69 ± 43M	50.12 ± 11.67	50.01 ± 12.35	CMC	MTX + NSAIDs	16W	①
Li, J. et al.	2017	47	45	3.2 ± 1.3Y	3.4 ± 1.2Y	51.6 ± 5.8	50.3 ± 6.1	MTX + NSAIDs + CMC	MTX + NSAIDs	12W	①④
Qian, X. et al.	2016	41	41	4.9 ± 1.1Y	4.3 ± 0.8Y	45.7 ± 12.6	46.4 ± 14.2	NASIDs + CMC	NASIDs	12W	①③
Cao, M. Z. et al.	2020	42	38	2.55 ± 0.69Y	2.65 ± 0.72Y	38.67 ± 9.52	39.11 ± 9.37	MTX + CMC	MTX	6W	①③
Yang, Y. J.	2013	60	60	2.1 ± 0.8Y	1.9 ± 0.9Y	32.2 ± 10.6	33.6 ± 10.2	CMC	LEF + NSAIDs + Thymosins	24W	①③
Zhou, C. Y. et al.	2010	50	50	52.74 ± 65.82M	50.37 ± 53.81M	48.03 ± 9.47	49.42 ± 10.16	CMC	MTX	12W	①④
Liu, X. D. et al.	2009	30	30	5.35 ± 2.61Y	4.65 ± 2.42Y	46.55 ± 9.21	47.86 ± 8.30	MTX + SSZ + NSAIDs + CMC	MTX + SSZ + NSAIDs	12W	①②④
Liu, B. et al.	2007	50	50	16M	15M	50	52	MTX + CMC	MTX	16W	①
Wei, Y. et al.	2013	80	80	3.1 ± 1.5Y	4.65 ± 2.42Y	35.1 ± 11.5	36.5 ± 10.1	LEF + SSZ + NSAIDs + CMC	LEF + SSZ + NSAIDs	12W	①④
Zheng, X. B. et al.	2015	30	30	52.3 ± 10.8M	53.6 ± 11.5M	49.6 ± 18.9	49.1 ± 19.4	MTX + CMC	MTX	12W	①④
Shu, C. et al.	2015	22	20	4.57 ± 3.41Y	4.08 ± 2.95Y	45.35 ± 10.42	46.59 ± 10.84	CMC	MTX + LEF	12W	①③④
Wei, W. et al.	2013	45	45	3.5 ± 2.2Y	3.4 ± 2.3Y	68.4 ± 2.5	69.2 ± 2.8	MTX + HCQ + NSAIDs + TNFi + CMC	MTX + HCQ + NSAIDs + TNFi	12W	①④
Han, S. L. et al.	2011	35	35	4.1 ± 1.35Y	3.88 ± 2.12Y	47.22 ± 8.13	46.86 ± 7.20	MTX + SSZ + NSAIDs + CMC	MTX + SSZ + NSAIDs	12W	①
Xu, G. S. et al.	2018	38	38	48.00M	46.50M	50.32 ± 10.99	48.56 ± 14.15	MTX + LEF + NSAIDs + CMC	MTX + LEF + NSAIDs	12W	①④
Pang, A. M. et al.	2017	39	39	7.61 ± 2.13M	6.85 ± 2.91M	41.63 ± 10.72	42.16 ± 9.82	MTX + NSAIDs + CMC	MTX + NSAIDs	12W	①②
Pang, J. et al.	2019	31	31	44.80 ± 27.40M	46.27 ± 28.92M	47.50 ± 13.04	46.13 ± 12.73	MTX + NSAIDs + CMC	MTX + NSAIDs	24W	①②③
Zhao, L.	2019	30	30	118.6 ± 49.6M	117.4 ± 46.5M	52.6 ± 6.8	53.4 ± 6.6	MTX + CMC	MTX	8W	①
Zeng, J. Y. et al.	2018	40	40	-	-	58.62 ± 5.09	58.74 ± 5.16	MTX + HCQ + NSAIDs + CMC	MTX + HCQ + NSAIDs	8W	①
Wang, H. T.	2017	40	40	3 ± 0.35Y	3 ± 0.21Y	42 ± 1.36	40 ± 1.32	CMC	LEF + NSAIDs	4W	①
Su, S. Z. et al.	2015	34	36	6 ± 2.3Y	5 ± 3.1Y	53 ± 1.2	55 ± 0.9	MTX + LEF + NSAIDs + CMC	MTX + LEF + NSAIDs	NR	①
Wang, Y. et al.	2017	52	52	4.7 ± 2.2Y	4.4 ± 2.1Y	45.1 ± 17.2	43.7 ± 14.8	MTX + LEF + CMC	MTX + LEF	12W	①③④
Gao, D. et al.	2017	60	60	7.73 ± 1.48Y	7.43 ± 1.37Y	55.02 ± 7.99	54.26 ± 6.78	MTX + CMC	MTX	12W	①②
Guo, H. L. et al.	2018	40	40	3.95 ± 3.00Y	3.67 ± 2.61Y	39.65 ± 12.99	40.83 ± 10.7	MTX + CMC	MTX	12W	①④
You, B. R. et al.	2016	43	43	2.1 ± 0.8Y	2.3 ± 0.6Y	42.3 ± 5.8	41.7 ± 5.2	MTX + CMC	MTX	24W	①④
Liu, E. C. et al.	2013	50	50	56.37 ± 65.30M	52.26 ± 52.61M	46.23 ± 9.68	49.62 ± 8.62	CMC	MTX	12W	①④
Li, S.W.	2006	31	30	5.17 ± 3.37Y	6.22 ± 4.65Y	49.5	51.7	MTX + SSZ + CMC	MTX + SSZ	8W	①④
Chen, F. et al.	2016	35	35	26.40 ± 6.12M	25.2 ± 4.48M	37.52 ± 6.61	37.34 ± 6.48	MTX + LEF + NSAIDs + CMC	MTX + LEF + NSAIDs	8W	①④
Li, Z. L. et al.	2012	30	31	1.15 ± 0.61Y	1.00 ± 0.60Y	50.40 ± 9.19	57.28 ± 11.39	CMC	Alfacalcidol capsule + caltrate D tablet	12W	①
Yang, B. et al.	2011	20	20	5.3Y	5.1Y	43	41	MTX + NSAIDs + CMC	MTX + NSAIDs	12W	①
Yao, J. H.	2010	58	38	4.08 ± 1.32Y	3.98 ± 1.42Y	36.52 ± 4.85	35.80 ± 4.92	CMC	LEF + NSAIDs + placebo	8W	①④
Shen, H. B. et al.	2011	40	40	32.5 ± 28.0M	38.0 ± 24.3M	49.0 ± 12.6	52.6 ± 10.1	CMC	MTX + NSAIDs	12W	①③
Chen, Z. W. et al.	2010	45	45	47.32 ± 48.32M	52.21 ± 41.31M	45.76 ± 10.21	46.23 ± 12.21	CMC	MTX	24W	①②③
Wang, Z. et al.	2014	47	41	3.8 ± 6.2Y	4.0 ± 6.4Y	42.82 ± 12.45	44.78 ± 12.38	MTX + LEF + CMC	MTX + LEF	12W	①③

EG, experimental group; CG, control group; CMC, Chinese medicine compound; SSZ, Sulfasalazine; MTX, Methotrexate; LEF, leflunomide; GC, glucocorticoid; HCQ, Hydroxychloroquine; NSAIDs, Nona-steroidal anti-inflammatory drugs; D-PEN, D-penicillamine; TNFi, TNF-α inhibitor; NR, Not Reported; W, weeks. ①RF; ②Anti-CCP; ③ACR20/ACR50/ACR70; ④ Standard of curative effect of TCM syndromes.

**TABLE 2:** A summary table describing the composition of the CMCs.

Study	Source	Species, concentration	Quality control reported? (Y/N)	Chemical analysis reported? (Y/N)
Li. (2006)	The First Affiliated Hospital of Henan College of Traditional Chinese Medicine	Gentiana macrophylla Pall.[Gentianaceae;Radix Gentianae Macrophyllae], 9 g; Angelica dahurica (Hoffm.) Benth. & Hook.f. ex Franch. & Sav. [Apiaceae;Radix Angelicae Pubescentis], 9 g; Saposhnikovia divaricata (Turcz. ex Ledeb.) Schischk. [Apiaceae;Radix Saposhnikoviae], 6 g; Asarum sieboldii[Aristolochiaceae; Herba cum Radix Asari], 3 g; Conioselinum anthriscoides ‘Chuanxiong’[Apiaceae;Rhizoma Ligustici], 9 g; Angelica sinensis (Oliv.) Diels[Apiaceae;Radix Angelicae Sinensis], 12 g; Rehmannia glutinosa (Gaertn.) DC. [Orobanchaceae;Radix Rehmanniae Preparata], 15 g; Paeonia lactiflora Pall.[Paeoniaceae;Radix Paeoniae Alba], 12 g; Neolitsea cassia (L.) Kosterm.[Lauraceae;Ramulus Cinnamomi], 6 g; Poria[Polyporaceae; Sclerotium Poriae Cocos], 12 g; Eucommia ulmoides Oliv. [Eucommiaceae; Cortex Eucommiae], 15 g; Achyranthes bidentata Blume [Amaranthaceae;Radix Achyranthis Bidentatae], 30 g; Codonopsis pilosula[Campanulaceae; Radix Codonopsis], 12 g; Glycyrrhiza glabra[Fabaceae; Radix Glycyrrhizae], 10 g; Astragalus mongholicus Bunge[Fabaceae;Radix Astragali seu Hedysari], 20 g; Dipsacus asper[Caprifoliaceae; Radix Dipsaci], 15 g; Citrus × aurantium L.[Rutaceae;Pericarpium Citri Reticulatae], 9 g; Zingiber officinale[Zingiberaceae; Rhizoma Zingiberis], 9 g	N	N
Huang et al. (2006)	Xiangya Hospital of Central South University & Hengyang Hospital affiliated with Hunan College of Traditional Chinese Medicine	Centipede[Scolopendridae; Scolopendra], 1 g; Pheretima asiatica Michaelsen[Megascolecidae;Lumbricus], 10 g; Agkistrodon[Deinagkistrodon;Bungarus], 1 g; Aconitum carmichaeli Debeaux [Ranunculaceae;Radix Aconiti], 3g(decocted earlier); Lindera aggregata (Sims) Kosterm. [Lauraceae; Radix Linderae], 3g(decocted earlier); Calamus draco Willd. [Calamus draco Willd.; Sanguis Draconis], 3 g; Arisaema heterophyllum Blume [Araceae; Rhizoma Arisaematis], 5g(decocted earlier); Eupolyphaga[Corydidae;Eupolyphaga Seu Steleophaga], 5 g; Smilax nipponica [Magnoliaceae; Dashenjin], 10 g; Clematis chinensis Osbeck[Ranunculaceae;Radix Clematidis], 10 g; Frankincense[Burseraceae; Olibanum], 10 g; Commiphora myrrha[Burseraceae; Resina Commiphorae], 10 g; Dipsacus asper[Caprifoliaceae; Radix Dipsaci], 10 g; Angelica dahurica (Hoffm.) Benth. & Hook.f. ex Franch. & Sav.[Apiaceae;Radix Angelicae Dahuricae], 10 g; Garden Balsam Stem [Euphorbiaceae; Speranskia tuberculata (Bunge) Baill], 10 g; Angelica sinensis (Oliv.) Diels[Apiaceae;Radix Angelicae Sinensis], 20 g; Conioselinum anthriscoides ‘Chuanxiong’[Apiaceae;Rhizoma Ligustici], 20 g; Glycyrrhiza glabra[Fabaceae; Radix Glycyrrhizae], 20 g	Y-Prepared according to *Chinese Pharmacopeia 2005*	N
Wang et al. (2006)	Shiyan City Hospital of Traditional Chinese Medicine	Rehmannia glutinosa (Gaertn.) DC. [Orobanchaceae;Radix Rehmanniae Preparata], 12 g; Paeonia lactiflora Pall. [Paeoniaceae;Radix Paeoniae Rubra], 10 g; Paeonia lactiflora Pall.[Paeoniaceae;Radix Paeoniae Alba], 10 g; Angelica sinensis (Oliv.) Diels[Apiaceae;Radix Angelicae Sinensis], 15 g; Conioselinum anthriscoides ‘Chuanxiong’[Apiaceae;Rhizoma Ligustici], 10 g; Sinomenium acutum[Menispermaceae; Caulis Sinomenii], 15-30 g; Tripterygium wilfordii[Celastraceae; Radix Tripterygii Wilfordii], 10 g; Trachelospermum jasminoides (Lindl.) Lem. [Apocynaceae; Caulis Trachelospermi], 15-30 g; Spatholobus suberectus Dunn[Fabaceae;Caulis Spatholobi], 30 g; Reynoutria multiflora (Thunb.) Moldenke [Polygonaceae; Caulis Polygoni Multiflori], 30 g	N	N
Liu et al. (2007)	The First Affiliated Hospital of Tianjin College of Traditional Chinese Medicine	Angelica sinensis (Oliv.) Diels[Apiaceae;Radix Angelicae Sinensis], 10 g; Conioselinum anthriscoides ‘Chuanxiong’[Apiaceae;Rhizoma Ligustici], 8 g; Paeonia lactiflora Pall.[Paeoniaceae;Radix Paeoniae Alba], 10 g; Rehmannia glutinosa (Gaertn.) DC. [Orobanchaceae;Radix Rehmanniae Preparata], 15 g	N	N
Luo et al. (2008)	Hospital attached to Beiliu Health School of Guangxi Autonomous Region	Astragalus mongholicus Bunge[Fabaceae;Radix Astragali seu Hedysari], 30 g; Codonopsis pilosula[Campanulaceae; Radix Codonopsis], 15 g; Atractylodes macrocephala Koidz.[Asteraceae;Rhizoma Atractylodis Macrocephalae], 15 g; Paeonia × suffruticosa [Paeoniaceae;Cortex Moutan Radicis], 15 g; Gentiana macrophylla Pall.[Gentianaceae;Radix Gentianae Macrophyllae], 15 g; Zaocys dhumnades(Cantor)[Natricinae;Zaocys dhumnades], 15 g; Dioscorea oppositifolia[Dioscoreaceae; Rhizoma Dioscoreae], 18 g; Coix lacryma-jobi L.[Poaceae;Semen Coicis], 18 g; Curcuma longa[Zingiberaceae; Rhizoma Curcumae Longae], 12 g; Bombyx mori Linnaeus[Bombyx Linnaeus;Bombyx Batryticatus], 9 g; Carthamus tinctorius[Asteraceae; Flos Carthami], 6 g; Buthus martensii Karsch[Scorpiones;Scorpio], 3 g; Asarum sieboldii[Aristolochiaceae; Herba cum Radix Asari], 3 g	N	N
Shen et al. (2008)	Hongmiaopo Hospital, Xi'an, Shaanxi Province	Rehmannia glutinosa[Orobanchaceae; Radix Rehmanniae]; Ophiopogon japonicus (Thunb.) Ker Gawl. [Asparagaceae; Radix Ophiopogonis]; Rehmannia glutinosa (Gaertn.) DC. [Orobanchaceae;Radix Rehmanniae Preparata] ;Eucommia ulmoides Oliv. [Eucommiaceae; Cortex Eucommiae] ; Achyranthes bidentata Blume [Amaranthaceae;Radix Achyranthis Bidentatae] ; Taxillus chinensis (DC.) Danser[Loranthaceae; Herba Taxilli] ; Astragalus mongholicus Bunge[Fabaceae;Radix Astragali seu Hedysari] ; Pseudostellaria heterophylla (Miq.) Pax [Caryophyllaceae; Radix Pseudostellariae heterophylly] ; Angelica sinensis (Oliv.) Diels[Apiaceae;Radix Angelicae Sinensis] ;Salvia miltiorrhiza Bunge[Lamiaceae; Radix Salviae Miltiorrhizae]; Polygonatum sibiricum Redouté [Asparagaceae; Rhizoma Polygonati];Gentiana macrophylla Pall.[Gentianaceae;Radix Gentianae Macrophyllae]; Angelica dahurica (Hoffm.) Benth. & Hook.f. ex Franch. & Sav. [Apiaceae;Radix Angelicae Pubescentis] ; Spatholobus suberectus Dunn[Fabaceae;Caulis Spatholobi]; Silkworm shit [Bombycidae; Bombyx mori L.]; Glycyrrhiza glabra[Fabaceae; Radix Glycyrrhizae]	N	N
Xiang et al. (2009)	The First Affiliated Hospital of Guangxi College of Traditional Chinese Medicine	Gentiana macrophylla Pall.[Gentianaceae;Radix Gentianae Macrophyllae], 15 g; Eucommia ulmoides Oliv. [Eucommiaceae; Cortex Eucommiae], 15 g; Dipsacus asper[Caprifoliaceae; Radix Dipsaci], 15 g; Rehmannia glutinosa (Gaertn.) DC. [Orobanchaceae;Radix Rehmanniae Preparata], 15 g; Angelica dahurica (Hoffm.) Benth. & Hook.f. ex Franch. & Sav. [Apiaceae;Radix Angelicae Pubescentis], 15 g; Taxillus chinensis (DC.) Danser[Loranthaceae; Herba Taxilli], 15 g; Reynoutria multiflora (Thunb.) Moldenke[Polygonaceae;Radix Polygoni Multiflori], 15 g; Lindera aggregata (Sims) Kosterm. [Lauraceae; Radix Linderae], 15 g; Pheretima asiatica Michaelsen[Megascolecidae;Lumbricus], 10 g; Bombyx mori Linnaeus[Bombyx Linnaeus;Bombyx Batryticatus], 10 g; Neolitsea cassia[Lauraceae; Cortex Cinnamomi], 10 g; Astragalus mongholicus Bunge[Fabaceae;Radix Astragali seu Hedysari], 30 g	N	N
Ma et al. (2009)	The Second Affiliated Hospital of Guiyang College of Traditional Chinese Medicine	Panax notoginseng (Burkill) F.H.Chen[Araliaceae;Radix Notoginseng]; Lindera aggregata (Sims) Kosterm. [Lauraceae; Radix Linderae]; Sinomenium acutum[Menispermaceae; Caulis Sinomenii]; Cibotium barometz (L.) J.Sm.[Cyatheaceae;Rhizoma Cibotii]; Homalomena occulta (Lour.) Schott [Araceae; Rhizoma Homalomenae]; Curcuma longa[Zingiberaceae; Rhizoma Curcumae Longae]; Paeonia lactiflora Pall.[Paeoniaceae;Radix Paeoniae Alba]; etc.	Y-Prepared according to *Chinese Pharmacopeia 2005*	N
Li et al. (2009)	Qingdao Fifth People's Hospital	Tripterygium wilfordii[Celastraceae; Radix Tripterygii Wilfordii], 15 g; Sinomenium acutum[Menispermaceae; Caulis Sinomenii], 20 g; Lonicera japonica Thunb. [Caprifoliaceae;Caulis Lonicerae], 20 g; Spatholobus suberectus Dunn[Fabaceae;Caulis Spatholobi], 30 g; Morus alba[Moraceae; Ramulus Mori], 30 g; Paeonia lactiflora Pall.[Paeoniaceae;Radix Paeoniae Alba], 30 g; Anemarrhena asphodeloides[Liliaceae; Rhizoma Anemarrhenae], 8 g; Saposhnikovia divaricata (Turcz. ex Ledeb.) Schischk. [Apiaceae;Radix Saposhnikoviae], 12 g; Epimedium sagittatum (Siebold & Zucc.) Maxim. [Berberidaceae;Herba Epimedii], 15 g; Conioselinum anthriscoides ‘Chuanxiong’[Apiaceae;Rhizoma Ligustici], 15 g; Pheretima asiatica Michaelsen[Megascolecidae;Lumbricus], 12 g; Buthus martensii Karsch[Scorpiones;Scorpio], 10 g; Taxillus chinensis (DC.) Danser[Loranthaceae; Herba Taxilli], 15 g; Angelica sinensis (Oliv.) Diels[Apiaceae;Radix Angelicae Sinensis], 10 g; Rehmannia glutinosa (Gaertn.) DC. [Orobanchaceae;Radix Rehmanniae Preparata], 15 g	N	N
Liu et al. (2009)	Zhejiang Integrated Chinese and Western Medicine Hospital	Saposhnikovia divaricata (Turcz. ex Ledeb.) Schischk. [Apiaceae;Radix Saposhnikoviae] ;Angelica dahurica (Hoffm.) Benth. & Hook.f. ex Franch. & Sav.[Apiaceae;Radix Angelicae Dahuricae]; Clematis chinensis Osbeck[Ranunculaceae;Radix Clematidis]; Buthus martensii Karsch[Scorpiones;Scorpio] ; Centipede[Scolopendridae; Scolopendra] ; Sinapis alba L. [Brassicaceae;Semen sinapis.]; Lonicera japonica Thunb. [Caprifoliaceae;Caulis Lonicerae] ; Salvia miltiorrhiza Bunge[Lamiaceae; Radix Salviae Miltiorrhizae] ; Coix lacryma-jobi L.[Poaceae;Semen Coicis] ; Bombyx mori Linnaeus[Bombyx Linnaeus;Bombyx Batryticatus] ; Corydalis yanhusuo[Papaveraceae;Rhizoma Corydalis] ; Atractylodes macrocephala Koidz.[Asteraceae;Rhizoma Atractylodis Macrocephalae]	Y-Prepared according to *Chinese Pharmacopeia 2005*	N
Chen et al. (2010)	Shanghai Hospital of Traditional Chinese Medicine	Hansenia weberbaueriana (Fedde ex H.Wolff) Pimenov & Kljuykov [Apiaceae;Rhizoma et Radix Notopterygii], 30 g; Rehmannia glutinosa[Orobanchaceae; Radix Rehmanniae], 30 g; Astragalus mongholicus Bunge[Fabaceae;Radix Astragali seu Hedysari], 30 g; Aconitum carmichaeli Debeaux [Ranunculaceae;Radix Aconiti], 9 g; Sauromatum giganteum (Engl.) Cusimano [Araceae;Rhizoma Typhonii ], 9 g; Caragana sinica (Buc’hoz) Rehde, 30 g; Rumex japonicus Houtt. [Polygonaceae; Radix Rumicis Japonici], 30 g; Sinapis alba L. [Brassicaceae;Semen sinapis.], 12 g; Curcuma longa[Zingiberaceae; Rhizoma Curcumae Longae], 12 g	Y-Prepared according to *Chinese Pharmacopeia 2005*	N
Yao et al. (2010)	Heze Hospital of Traditional Chinese Medicine	Tripterygium wilfordii[Celastraceae; Radix Tripterygii Wilfordii], (decocted earlier)20 g; Sarcandra glabra (Thunb.) Nakai[Chloranthaceae;Herba Sarcandrae], 15 g; Lonicera japonica [Caprifoliaceae;Flos Lonicerae], 24 g; Equus asinus L. [Equidae; Colla Corii Asini], 24 g; Scleromitrion diffusum (Willd.) R.J.Wang[Rubiaceae;Herba Hedyotis], 30 g; Phellodendron amurense Rupr. [Rutaceae;Cortex Phellodendri], 12 g; Atractylodes lancea (Thunb.) DC.[Asteraceae;Rhizoma Atractylodis], 9 g; Coix lacryma-jobi L.[Poaceae;Semen Coicis], 30 g; TuPoria[Polyporaceae; Sclerotium Poriae Cocos], 20 g; Bolbostemma paniculatum (Maxim.) Franquet [Cucurbitaceae;Rhizoma Bolbostematis ], 12 g; Paeonia lactiflora Pall. [Paeoniaceae;Radix Paeoniae Rubra], 20 g; Hansenia weberbaueriana (Fedde ex H.Wolff) Pimenov & Kljuykov [Apiaceae;Rhizoma et Radix Notopterygii], 9 g; Angelica dahurica (Hoffm.) Benth. & Hook.f. ex Franch. & Sav. [Apiaceae;Radix Angelicae Pubescentis], 9 g	N	N
Zhou et al. (2010)	Xiyuan Hospital, Chinese Academy of Traditional Chinese Medicine	Lonicera japonica [Caprifoliaceae;Flos Lonicerae], 30 g; Angelica sinensis (Oliv.) Diels[Apiaceae;Radix Angelicae Sinensis], 20 g; Scrophularia ningpoensis[Scrophulariaceae; Radix Scrophulariae], 20 g; Glycyrrhiza glabra[Fabaceae; Radix Glycyrrhizae], 10 g; Scleromitrion diffusum (Willd.) R.J.Wang[Rubiaceae;Herba Hedyotis], 30 g; Cremastra appendiculata (D.Don) Makino[Orchidaceae;Pseudobulbus Cremastrae seu Pleiones], 9 g; Sigesbeckia orientalis[Asteraceae; Herba Siegesbeckiae], 30 g; Reynoutria japonica Houtt. [Polygonaceae; Rhizoma Polygoni Cuspidati], 15 g; TuPoria[Polyporaceae; Sclerotium Poriae Cocos], 20 g; Paeonia lactiflora Pall.[Paeoniaceae;Radix Paeoniae Alba], 30 g; Clematis chinensis Osbeck[Ranunculaceae;Radix Clematidis], 20 g; Dioscorea collettii var. hypoglauca (Palib.) S.J.Pei & C.T.Ting[Dioscoreaceae;Rhizome Dioscoreae Hypoglaucae], 20 g	N	N
Shen et al. (2011)	the Third Hospital of Beijing University, Beijing Military General Hospital and Wangjing Hospital of China Academy of Chinese Medical Sciences	Erodium stephanianum Willd. [Geraniaceae; Herba Geranii], 15 g; Manis pentadactyla Linnaeus [Manidae; Radix Actinidiae chinensis], 15 g; Sigesbeckia orientalis[Asteraceae; Herba Siegesbeckiae], 15 g; TuPoria[Polyporaceae; Sclerotium Poriae Cocos], 15 g; Reynoutria japonica Houtt. [Polygonaceae; Rhizoma Polygoni Cuspidati], 15 g; Sanguisorba officinalis L. [Rosaceae; Radix Sanguisorbae] , 15 g; Xuchangqin g 10 g; Ephedra sinica Stapf [Ephedraceae;Herba Ephedrae], 5 g	N	N
Han and Song (2011)	Hangzhou Xixi Street Community Health Service Center	Clematis chinensis Osbeck[Ranunculaceae;Radix Clematidis], 30 g; Buthus martensii Karsch[Scorpiones;Scorpio], 3 pieces; Centipede[Scolopendridae; Scolopendra], 1 piece; Sinapis alba L. [Brassicaceae;Semen sinapis.], 12 g; Salvia miltiorrhiza Bunge[Lamiaceae; Radix Salviae Miltiorrhizae], 20 g; Coix lacryma-jobi L.[Poaceae;Semen Coicis], 30 g	N	N
Yang et al. (2011)	Institute of Integrated Chinese and Western Medicine, Xiangya Hospital, Central South University	Atractylodes lancea (Thunb.) DC.[Asteraceae;Rhizoma Atractylodis], 12 g; Phellodendron amurense Rupr. [Rutaceae;Cortex Phellodendri], 12 g; Achyranthes bidentata Blume [Amaranthaceae;Radix Achyranthis Bidentatae], 12 g; Coix lacryma-jobi L.[Poaceae;Semen Coicis], 12 g	Y-Prepared according to *Chinese Pharmacopeia 2005*	N
Li et al. (2012)	Shanghai Guanghua Hospital of Integrative Medicine	Kidney-Yin deficiency syndrome: Rehmannia glutinosa (Gaertn.) DC. [Orobanchaceae;Radix Rehmanniae Preparata], 15 g; Shanyu 12 g; Eucommia ulmoides Oliv. [Eucommiaceae; Cortex Eucommiae], 12 g; Dipsacus asper[Caprifoliaceae; Radix Dipsaci], 10 g; Psoralea fructus[Fabaceae; Fructus Psoraliae], 15 g; Angelica sinensis (Oliv.) Diels[Apiaceae;Radix Angelicae Sinensis], 10 g; Achyranthes bidentata Blume [Amaranthaceae;Radix Achyranthis Bidentatae], 15 g; Pheretima asiatica Michaelsen[Megascolecidae;Lumbricus], 10g. kidney-Yang deficiency syndrome: Aconitum carmichaeli[Ranunculaceae; Radix Aconiti Lateralis Preparata], 10 g; Curculigo orchioides Gaertn.[Hypoxidaceae;Rhizoma Curculigins], 30 g; Gynochthodes officinalis (F.C.How) Razafim. [Rubiaceae; Radix Morindae Officinalis], 15 g; Epimedium sagittatum (Siebold & Zucc.) Maxim. [Berberidaceae;Herba Epimedii], 12 g; Angelica sinensis (Oliv.) Diels[Apiaceae;Radix Angelicae Sinensis], 10 g; Buthus martensii Karsch[Scorpiones;Scorpio], 2 g	N	N
Liu et al. (2013)	Anhui Mengcheng County Hospital of Traditional Chinese Medicine	Strychnos nux-vomica L. [Loganiaceae; Semen Strychni]; Frankincense[Burseraceae; Olibanum] ; Commiphora myrrha[Burseraceae; Resina Commiphorae] ; Glycyrrhiza glabra[Fabaceae; Radix Glycyrrhizae]; etc.	N	N
Wei et al. (2013)	Hangzhou Economic and Technological Development Zone Maternal and Child Health Hospital	Lonicera japonica [Caprifoliaceae;Flos Lonicerae], 30 g; Scrophularia ningpoensis[Scrophulariaceae; Radix Scrophulariae], 20 g; Angelica sinensis (Oliv.) Diels[Apiaceae;Radix Angelicae Sinensis], 20 g; Glycyrrhiza glabra[Fabaceae; Radix Glycyrrhizae], 10 g; Chrysanthemum × morifolium (Ramat.) Hemsl.[Asteraceae;Flos Chrysanthemi], 20 g; Phellodendron amurense Rupr. [Rutaceae;Cortex Phellodendri], 12 g; Atractylodes lancea (Thunb.) DC.[Asteraceae;Rhizoma Atractylodis],12 g; TuPoria[Polyporaceae; Sclerotium Poriae Cocos], 20 g; Achyranthes bidentata Blume [Amaranthaceae;Radix Achyranthis Bidentatae], 12 g; Sinomenium acutum[Menispermaceae; Caulis Sinomenii], 20 g; Zaocys dhumnades(Cantor)[Natricinae;Zaocys dhumnades], 6 g; Pheretima asiatica Michaelsen[Megascolecidae;Lumbricus], 10 g	N	N
Li (2013)	The Second Affiliated Hospital of Tianjin University of Traditional Chinese Medicine	Caragana sinica (Buc’hoz) Rehde, 30 g; Scrophularia ningpoensis[Scrophulariaceae; Radix Scrophulariae], 25 g; Coix lacryma-jobi L.[Poaceae;Semen Coicis], 25 g; Hansenia weberbaueriana (Fedde ex H.Wolff) Pimenov & Kljuykov [Apiaceae;Rhizoma et Radix Notopterygii], 15 g; Angelica dahurica (Hoffm.) Benth. & Hook.f. ex Franch. & Sav. [Apiaceae;Radix Angelicae Pubescentis], 15 g; Sinomenium acutum[Menispermaceae; Caulis Sinomenii], 15 g; Paeonia lactiflora Pall. [Paeoniaceae;Radix Paeoniae Rubra], 15 g; Sigesbeckia orientalis[Asteraceae; Herba Siegesbeckiae],15 g; Atractylodes lancea (Thunb.) DC.[Asteraceae;Rhizoma Atractylodis],15 g; Salvia miltiorrhiza Bunge[Lamiaceae; Radix Salviae Miltiorrhizae], 10 g; Clematis chinensis Osbeck[Ranunculaceae;Radix Clematidis], 10 g; Bombyx mori Linnaeus[Bombyx Linnaeus;Bombyx Batryticatus], 10 g; Eupolyphaga[Corydidae;Eupolyphaga Seu Steleophaga], 5 g	N	N
Wei et al. (2013)	People's Hospital of Yuyao City, Zhejiang Province	Centipede[Scolopendridae; Scolopendra], 2 pieces; Agkistrodon[Deinagkistrodon;Bungarus], 1 piece; Buthus martensii Karsch[Scorpiones;Scorpio], 10 g; Hansenia weberbaueriana (Fedde ex H.Wolff) Pimenov & Kljuykov [Apiaceae;Rhizoma et Radix Notopterygii], 15 g; Angelica dahurica (Hoffm.) Benth. & Hook.f. ex Franch. & Sav. [Apiaceae;Radix Angelicae Pubescentis], 15 g; Corydalis yanhusuo[Papaveraceae;Rhizoma Corydalis], 15 g; Gentiana macrophylla Pall.[Gentianaceae;Radix Gentianae Macrophyllae], 15 g; Stephania tetrandra[Menispermaceae; Radix Stephaniae Tetrandrae], 15 g; Poria[Polyporaceae; Sclerotium Poriae Cocos], 20 g; Liquidambar formosana Hance [Altingiaceae; Fructus Liquidambaris], 20 g; Astragalus mongholicus Bunge[Fabaceae;Radix Astragali seu Hedysari], 30 g; Codonopsis pilosula[Campanulaceae; Radix Codonopsis], 30 g; Atractylodes macrocephala Koidz.[Asteraceae;Rhizoma Atractylodis Macrocephalae], 15 g; Glycyrrhiza glabra[Fabaceae; Radix Glycyrrhizae], 10 g	N	N
Yang et al. (2013)	The Third Hospital of Baoding City, Hebei Province	Strychnos nux-vomica L. [Loganiaceae; Semen Strychni], 0.2 g; Dioscorea nipponica Makino[Dioscoreaceae;Rhizoma Dioscoreae Nipponicae], 8 g; Rhododendron molle (Blume) G.Don [Ericaceae; Rhododendri mollis flos], 0.2 g; Epimedium sagittatum (Siebold & Zucc.) Maxim. [Berberidaceae;Herba Epimedii], 3 g; Paeonia lactiflora Pall.[Paeoniaceae;Radix Paeoniae Alba], 3 g; Wushe 3 g; Dibiechong 3 g; Sarcandra glabra (Thunb.) Nakai[Chloranthaceae;Herba Sarcandrae], 4 g	N	N
Wang Tao (2014)	Zhejiang Integrated Chinese and Western Medicine Hospital	Yangtao Actinidia Root [Actinidiaceae; Actinidia chinensis Planch.], 30 g; Pheretima asiatica Michaelsen[Megascolecidae;Lumbricus], 10 g; Bombyx mori Linnaeus[Bombyx Linnaeus;Bombyx Batryticatus], 10 g; Poria[Polyporaceae; Sclerotium Poriae Cocos], 15 g; Glycyrrhiza glabra[Fabaceae; Radix Glycyrrhizae], 5 g; Spatholobus suberectus Dunn[Fabaceae;Caulis Spatholobi], 30 g; Angelica sinensis (Oliv.) Diels[Apiaceae;Radix Angelicae Sinensis], 15 g; Rehmannia glutinosa (Gaertn.) DC. [Orobanchaceae;Radix Rehmanniae Preparata], 15 g; Coix lacryma-jobi L.[Poaceae;Semen Coicis], 30 g; Astragalus mongholicus Bunge[Fabaceae;Radix Astragali seu Hedysari], 30 g; Paeonia lactiflora Pall. [Paeoniaceae;Radix Paeoniae Rubra], 15 g	Y-Prepared according to *Chinese Pharmacopeia 2010*	N
He Xiao (2014)	Wuhan General Hospital of Guangzhou Military Region	Deerhorn glue[Cervidae;Colla Corni Cervi], 10 g; Aconitum carmichaeli[Ranunculaceae; Radix Aconiti Lateralis Preparata], 10 g; Neolitsea cassia (L.) Kosterm.[Lauraceae;Ramulus Cinnamomi], 10 g; Angelica dahurica (Hoffm.) Benth. & Hook.f. ex Franch. & Sav. [Apiaceae;Radix Angelicae Pubescentis], 10 g; Asarum sieboldii[Aristolochiaceae; Herba cum Radix Asari], 5 g; Angelica sinensis (Oliv.) Diels[Apiaceae;Radix Angelicae Sinensis], 15 g; Clematis chinensis Osbeck[Ranunculaceae;Radix Clematidis], 15 g; Sinomenium acutum[Menispermaceae; Caulis Sinomenii], 20 g; Paeonia lactiflora Pall. [Paeoniaceae;Radix Paeoniae Rubra], 10 g; Paeonia lactiflora Pall.[Paeoniaceae;Radix Paeoniae Alba], 10 g; Pheretima asiatica Michaelsen[Megascolecidae;Lumbricus], 10 g; Coix lacryma-jobi L.[Poaceae;Semen Coicis], 30 g; Rehmannia glutinosa[Orobanchaceae; Radix Rehmanniae], 10 g; Glycyrrhiza glabra[Fabaceae; Radix Glycyrrhizae], 15 g; Centipede[Scolopendridae; Scolopendra], 2 pieces ; Vincetoxicum mukdenense Kitag.[Apocynaceae;Radix Cynanchi Paniculati], 15 g	Y-Prepared according to *Chinese Pharmacopeia 2010*	N
Zhang et al. (2014)	263 Clinical Department, General Hospital of Beijing Military Region	Angelica dahurica (Hoffm.) Benth. & Hook.f. ex Franch. & Sav. [Apiaceae;Radix Angelicae Pubescentis], 15 g; Clematis chinensis Osbeck[Ranunculaceae;Radix Clematidis], 15 g; Buthus martensii Karsch[Scorpiones;Scorpio], 6 g; Pheretima asiatica Michaelsen[Megascolecidae; Lumbricus], 12 g; Atractylodes lancea (Thunb.) DC.[Asteraceae; Rhizoma Atractylodis], 12 g;Neolitsea cassia (L.) Kosterm.[Lauraceae; Ramulus Cinnamomi], 10 g; Phellodendron amurense Rupr. [Rutaceae; Cortex Phellodendri], 10 g; Zhechong10 g; Wuqiaoshe 10 g; Carthamus tinctorius[Asteraceae; Flos Carthami], 10 g; Achyranthes bidentata Blume [Amaranthaceae; Radix Achyranthis Bidentatae], 15 g; Curcuma longa[Zingiberaceae; Rhizoma Curcumae Longae], 15 g	N	N
Niu (2014)	Xi'an Hospital of Traditional Chinese Medicine	Paeonia lactiflora Pall.[Paeoniaceae;Radix Paeoniae Alba], 20 g; Chaenomeles lagenaria (Loisel.) Koidz. [Rosaceae; Fructus Chaenomelis], 15 g; Pyrola calliantha Andres [Ericaceae; Herba Pyrolae], 15 g; Lycopodium japonicum[Lycopodiaceae; Herba Lycopodii], 15 g; Atractylodes macrocephala Koidz.[Asteraceae;Rhizoma Atractylodis Macrocephalae], 15 g; Neolitsea cassia (L.) Kosterm.[Lauraceae;Ramulus Cinnamomi], 12 g; Gentiana macrophylla Pall.[Gentianaceae;Radix Gentianae Macrophyllae], 12 g; Piper kadsura[Piperaceae; Caulis Piperis Kadsurae], 12 g; Aconitum carmichaeli Debeaux [Ranunculaceae;Radix Aconiti], 10 g; Saposhnikovia divaricata (Turcz. ex Ledeb.) Schischk. [Apiaceae;Radix Saposhnikoviae], 10 g; Angelica sinensis (Oliv.) Diels[Apiaceae;Radix Angelicae Sinensis], 10 g; Achyranthes bidentata Blume [Amaranthaceae;Radix Achyranthis Bidentatae], 10 g; Frankincense[Burseraceae; Olibanum], 10 g; Cremastra appendiculata (D.Don) Makino[Orchidaceae;Pseudobulbus Cremastrae seu Pleiones], 10 g; Glycyrrhiza glabra[Fabaceae; Radix Glycyrrhizae], 6 g; Ephedra sinica Stapf [Ephedraceae;Herba Ephedrae], 4 g	N	N
Cao et al. (2014)	Anhui Provincial Hospital of Traditional Chinese Medicine	Compound Qiyi Capsule: Coix lacryma-jobi L.[Poaceae;Semen Coicis] ; Astragalus mongholicus Bunge[Fabaceae;Radix Astragali seu Hedysari] ; Centipede[Scolopendridae; Scolopendra] ; Tripterygium wilfordii[Celastraceae; Radix Tripterygii Wilfordii],; Astragalus mongholicus Bunge[Fabaceae;Radix Astragali seu Hedysari],n Qingre Chubi Capsules: Astragalus mongholicus Bunge[Fabaceae;Radix Astragali seu Hedysari],n ; Gardenia jasminoides J.Ellis[Rubiaceae;Fructus Gardeniae] ; Prunus persica (L.) Batsch[Rosaceae;Semen Persicae] ; Clematis chinensis Osbeck[Ranunculaceae;Radix Clematidis]; etc.	Y-Prepared according to *Chinese Pharmacopeia 2010*	N
Yu and Chen (2014)	Guangxi Yongfu County People's Hospital	Coix lacryma-jobi L.[Poaceae;Semen Coicis], 20 g; Atractylodes lancea (Thunb.) DC.[Asteraceae;Rhizoma Atractylodis], 12 g; Glycyrrhiza glabra[Fabaceae; Radix Glycyrrhizae], 6 g; Hansenia weberbaueriana (Fedde ex H.Wolff) Pimenov & Kljuykov [Apiaceae;Rhizoma et Radix Notopterygii], 10 g; Angelica dahurica (Hoffm.) Benth. & Hook.f. ex Franch. & Sav. [Apiaceae;Radix Angelicae Pubescentis], 10 g; Saposhnikovia divaricata (Turcz. ex Ledeb.) Schischk. [Apiaceae;Radix Saposhnikoviae], 10 g; Ephedra sinica Stapf [Ephedraceae;Herba Ephedrae], 6 g; Neolitsea cassia (L.) Kosterm.[Lauraceae;Ramulus Cinnamomi], 10 g; Aconitum carmichaeli Debeaux [Ranunculaceae;Radix Aconiti], 10 g; Angelica sinensis (Oliv.) Diels[Apiaceae;Radix Angelicae Sinensis], 8 g; Conioselinum anthriscoides ‘Chuanxiong’[Apiaceae;Rhizoma Ligustici], 8 g	N	N
Su et al. (2015)	Dongguan City Hospital of Traditional Chinese Medicine	Angelica dahurica (Hoffm.) Benth. & Hook.f. ex Franch. & Sav. [Apiaceae;Radix Angelicae Pubescentis], 20 g; Taxillus chinensis (DC.) Danser[Loranthaceae; Herba Taxilli], 30 g; Eucommia ulmoides Oliv. [Eucommiaceae; Cortex Eucommiae], 15 g; Asarum sieboldii[Aristolochiaceae; Herba cum Radix Asari], 3 g; Angelica sinensis (Oliv.) Diels[Apiaceae;Radix Angelicae Sinensis], 10 g; Paeonia lactiflora Pall.[Paeoniaceae;Radix Paeoniae Alba], 20 g; Glycyrrhiza glabra[Fabaceae; Radix Glycyrrhizae], 6 g; Rehmannia glutinosa (Gaertn.) DC. [Orobanchaceae;Radix Rehmanniae Preparata], 15 g; Conioselinum anthriscoides ‘Chuanxiong’[Apiaceae;Rhizoma Ligustici], 15 g; Neolitsea cassia (L.) Kosterm.[Lauraceae;Ramulus Cinnamomi], 15 g; Codonopsis pilosula[Campanulaceae; Radix Codonopsis], 15 g; Gleditsia sinensis Lam. [Fabaceae; Spina Gleditsiae], 12 g; Quanchong 5 g; Centipede[Scolopendridae; Scolopendra], 2 pieces; Spatholobus suberectus Dunn[Fabaceae;Caulis Spatholobi], 30 g	N	N
Shu et al. (2015)	Tongling Hospital of Traditional Chinese Medicine	Sophora flavescens Aiton[Fabaceae;Radix Sophorae Flavescentis], 9 g; Sinomenium acutum[Menispermaceae; Caulis Sinomenii], 9 g; Phellodendron amurense Rupr. [Rutaceae;Cortex Phellodendri], 9 g; Dioscorea collettii var. hypoglauca (Palib.) S.J.Pei & C.T.Ting[Dioscoreaceae;Rhizome Dioscoreae Hypoglaucae], 10 g; Astragalus mongholicus Bunge[Fabaceae;Radix Astragali seu Hedysari], 30 g; Angelica sinensis (Oliv.) Diels[Apiaceae;Radix Angelicae Sinensis], 15 g; Sargentodoxa cuneata (Oliv.) Rehder [Lardizabalaceae; Caulis Sargentodoxae], 15 g; Spatholobus suberectus Dunn[Fabaceae;Caulis Spatholobi], 15 g; Tripterygium wilfordii[Celastraceae; Radix Tripterygii Wilfordii], (decocted earlier)10 g; Equus asinus L. [Equidae; Colla Corii Asini], 25 g; Citrus × aurantium L.[Rutaceae;Pericarpium Citri Reticulatae], 9 g; Taxillus chinensis (DC.) Danser[Loranthaceae; Herba Taxilli], 15 g; Centipede[Scolopendridae; Scolopendra], 1 piece; Zaocys dhumnades(Cantor)[Natricinae;Zaocys dhumnades], 10 g	N	N
Li et al. (2015)	Yiji Mountain Hospital of Wannan Medical College	Astragalus mongholicus Bunge[Fabaceae;Radix Astragali seu Hedysari], 30 g; Equus asinus L. [Equidae; Colla Corii Asini], 20 g; Angelica sinensis (Oliv.) Diels[Apiaceae;Radix Angelicae Sinensis], 15 g; Sargentodoxa cuneata (Oliv.) Rehder [Lardizabalaceae; Caulis Sargentodoxae], 15 g; Spatholobus suberectus Dunn[Fabaceae;Caulis Spatholobi], 15 g; Cuscuta chinensis Lam. [Convolvulaceae;Semen Cuscutae] 12 g; Dioscorea collettii var. hypoglauca (Palib.) S.J.Pei & C.T.Ting[Dioscoreaceae;Rhizome Dioscoreae Hypoglaucae], 12 g; Epimedium sagittatum (Siebold & Zucc.) Maxim. [Berberidaceae;Herba Epimedii], 12 g; Sinomenium acutum[Menispermaceae; Caulis Sinomenii], 9 g; Pinellia ternata (Thunb.) Makino [Araceae; Rhizoma Pinelliae], 9 g; Sophora flavescens Aiton[Fabaceae;Radix Sophorae Flavescentis], 9 g; Phellodendron amurense Rupr. [Rutaceae;Cortex Phellodendri], 9 g	N	N
Zhao (2015)	Shandong Provincial Hospital of Traditional Chinese Medicine	Saposhnikovia divaricata (Turcz. ex Ledeb.) Schischk. [Apiaceae;Radix Saposhnikoviae], 30 g; Gypsum [Mineral; Gypsum Fibrosum], 30 g; Gardenia jasminoides J.Ellis[Rubiaceae;Fructus Gardeniae], 30 g; Astragalus mongholicus Bunge[Fabaceae;Radix Astragali seu Hedysari], 30 g; Citrus × aurantium L.[Rutaceae;Pericarpium Citri Reticulatae], 30 g;Atractylodes macrocephala Koidz.[Asteraceae;Rhizoma Atractylodis Macrocephalae], 25 g; Pogostemon cablin (Blanco) Benth. [Lamiaceae; Pogostemon Cablin (Blanco) Benth.] 15 g; Actaea cimicifuga L. [Ranunculaceae; Rhizoma Cimicifugae], 15 g; Bupleurum falcatum L. [Apiaceae; Radix Bupleuri], 15 g; Codonopsis pilosula[Campanulaceae; Radix Codonopsis], 25 g; Angelica sinensis (Oliv.) Diels[Apiaceae;Radix Angelicae Sinensis], 20 g; Glycyrrhiza glabra[Fabaceae; Radix Glycyrrhizae], 10 g	N	N
Li et al. (2015)	Fuyang People's Hospital, Anhui Province	Atractylodes lancea (Thunb.) DC.[Asteraceae;Rhizoma Atractylodis], 10 g; Phellodendron amurense Rupr. [Rutaceae;Cortex Phellodendri], 10 g; Stephania tetrandra[Menispermaceae; Radix Stephaniae Tetrandrae], 10 g; Conioselinum anthriscoides ‘Chuanxiong’[Apiaceae;Rhizoma Ligustici], 10 g; Hansenia weberbaueriana (Fedde ex H.Wolff) Pimenov & Kljuykov [Apiaceae;Rhizoma et Radix Notopterygii], 10 g; Angelica dahurica (Hoffm.) Benth. & Hook.f. ex Franch. & Sav.[Apiaceae;Radix Angelicae Dahuricae], 6 g; Clematis chinensis Osbeck[Ranunculaceae;Radix Clematidis], 10 g; Neolitsea cassia (L.) Kosterm.[Lauraceae;Ramulus Cinnamomi], 5 g; Sinapis alba L. [Brassicaceae;Semen sinapis.], 10 g; Prunus persica (L.) Batsch[Rosaceae;Semen Persicae], 10 g; Carthamus tinctorius[Asteraceae; Flos Carthami], 10 g; Zaocys dhumnades(Cantor)[Natricinae;Zaocys dhumnades], 10 g; Sigesbeckia orientalis[Asteraceae; Herba Siegesbeckiae], 30 g; Medicated leaven[Massa Medicata Fermentata], 10 g	N	N
Zheng and Shi (2015)	Jiangxi Guangfeng County People's Hospital	Aconitum carmichaeli Debeaux [Ranunculaceae;Radix Aconiti]; Aconitum kusnezoffii Rchb.[Ranunculaceae;Radix Aconiti Kusnezoffii]; Cyperus rotundus L. [Cyperaceae; Rhizoma Cyperi];Conioselinum anthriscoides ‘Chuanxiong’[Apiaceae;Rhizoma Ligustici]; Ephedra sinica Stapf [Ephedraceae;Herba Ephedrae],;Angelica sinensis (Oliv.) Diels[Apiaceae;Radix Angelicae Sinensis];Arisaema heterophyllum Blume [Araceae; Rhizoma Arisaematis];Angelica dahurica (Hoffm.) Benth. & Hook.f. ex Franch. & Sav.[Apiaceae;Radix Angelicae Dahuricae];Commiphora myrrha[Burseraceae; Resina Commiphorae];Frankincense[Burseraceae; Olibanum];Pheretima asiatica Michaelsen[Megascolecidae; Lumbricus]	N	N
Qian et al. (2016)	The First Affiliated Hospital of Nanjing University of Chinese Medicine	Angelica dahurica (Hoffm.) Benth. & Hook.f. ex Franch. & Sav. [Apiaceae;Radix Angelicae Pubescentis], 10 g; Eucommia ulmoides Oliv. [Eucommiaceae; Cortex Eucommiae], 15 g; Achyranthes bidentata Blume [Amaranthaceae;Radix Achyranthis Bidentatae], 10 g; Asarum sieboldii[Aristolochiaceae; Herba cum Radix Asari], 6 g; Panax ginseng C.A.Mey.[Araliaceae;Radix Ginseng], 10 g; Taxillus chinensis (DC.) Danser[Loranthaceae; Herba Taxilli], 20 g; Saposhnikovia divaricata (Turcz. ex Ledeb.) Schischk. [Apiaceae;Radix Saposhnikoviae], 10 g; Neolitsea cassia[Lauraceae; Cortex Cinnamomi], 10 g; Angelica sinensis (Oliv.) Diels[Apiaceae;Radix Angelicae Sinensis], 10 g; Gentiana macrophylla Pall.[Gentianaceae;Radix Gentianae Macrophyllae], 10 g; Conioselinum anthriscoides ‘Chuanxiong’[Apiaceae;Rhizoma Ligustici], 10 g; Glycyrrhiza glabra[Fabaceae; Radix Glycyrrhizae], 6 g; Paeonia lactiflora Pall.[Paeoniaceae; Radix Paeoniae Alba], 10 g	N	N
Jia et al. (2016)	Linfen Fourth People's Hospital	Astragalus mongholicus Bunge[Fabaceae;Radix Astragali seu Hedysari], 30 g; Angelica sinensis (Oliv.) Diels[Apiaceae;Radix Angelicae Sinensis], 20 g; Conioselinum anthriscoides ‘Chuanxiong’[Apiaceae;Rhizoma Ligustici], 15 g; Shudi 15 g; Saposhnikovia divaricata (Turcz. ex Ledeb.) Schischk. [Apiaceae;Radix Saposhnikoviae], 15 g; Hansenia weberbaueriana (Fedde ex H.Wolff) Pimenov & Kljuykov [Apiaceae;Rhizoma et Radix Notopterygii], 10 g; Angelica dahurica (Hoffm.) Benth. & Hook.f. ex Franch. & Sav. [Apiaceae;Radix Angelicae Pubescentis], 10 g; Neolitsea cassia (L.) Kosterm.[Lauraceae;Ramulus Cinnamomi], 15 g; Cucumis melo L. [Cucurbitaceae; Retinervus Luffae Fructus], 15 g; Gentiana macrophylla Pall.[Gentianaceae;Radix Gentianae Macrophyllae], 15 g; Piper kadsura[Piperaceae; Caulis Piperis Kadsurae], 12 g; Trachelospermum jasminoides (Lindl.) Lem. [Apocynaceae; Caulis Trachelospermi], 20 g; Uncaria rhynchophylla (Miq.) Miq. [Rubiaceae; Ramulus Uncariae Cum Uncis], 20 g; Spatholobus suberectus Dunn[Fabaceae;Caulis Spatholobi], 30 g; Paeonia lactiflora Pall.[Paeoniaceae;Radix Paeoniae Alba], 30 g; Paeonia lactiflora Pall. [Paeoniaceae;Radix Paeoniae Rubra], 15 g;Epimedium sagittatum (Siebold & Zucc.) Maxim. [Berberidaceae;Herba Epimedii], 15 g; Taxillus chinensis (DC.) Danser[Loranthaceae; Herba Taxilli], 12 g; Pheretima asiatica Michaelsen[Megascolecidae;Lumbricus], 12 g; Buthus martensii Karsch[Scorpiones;Scorpio], 10 g; Curcuma longa[Zingiberaceae; Rhizoma Curcumae Longae], 10 g; Coix lacryma-jobi L.[Poaceae;Semen Coicis],10 g; Glycyrrhiza glabra[Fabaceae; Radix Glycyrrhizae], 10 g	N	N
You et al. (2016)	The Fifth Affiliated Hospital of Xinjiang Medical University	Rehmannia glutinosa (Gaertn.) DC. [Orobanchaceae;Radix Rehmanniae Preparata], 15 g; Astragalus mongholicus Bunge[Fabaceae;Radix Astragali seu Hedysari],n 15 g; Shanyu 12 g; Paeonia × suffruticosa [Paeoniaceae;Cortex Moutan Radicis], 12 g; Poria[Polyporaceae; Sclerotium Poriae Cocos], 12 g; Coptis chinensis Franch.[Ranunculaceae;Rhizoma Coptidis], 12 g; Dioscorea oppositifolia[Dioscoreaceae; Rhizoma Dioscoreae], 10 g; Chrysanthemum × morifolium (Ramat.) Hemsl.[Asteraceae;Flos Chrysanthemi], 10 g; Alisma plantago-aquatica subsp. orientale (Sam.) Sam.[Alismataceae;Rhizoma Alismatis], 10 g; Angelica sinensis (Oliv.) Diels[Apiaceae;Radix Angelicae Sinensis], 10 g	N	N
Han et al. (2016)	Xinjiang Uygur Autonomous Region Hospital of Traditional Chinese Medicine	Zaocys dhumnades(Cantor)[Natricinae;Zaocys dhumnades], 12 g; Taxillus chinensis (DC.) Danser[Loranthaceae; Herba Taxilli], 20 g; Buthus martensii Karsch[Scorpiones;Scorpio], 6 g; Lonicera japonica Thunb. [Caprifoliaceae;Caulis Lonicerae], 30 g; Coix lacryma-jobi L.[Poaceae;Semen Coicis], 30 g; Atractylodes lancea (Thunb.) DC.[Asteraceae;Rhizoma Atractylodis],12 g; Astragalus mongholicus Bunge[Fabaceae;Radix Astragali seu Hedysari], 30 g; Saposhnikovia divaricata (Turcz. ex Ledeb.) Schischk. [Apiaceae;Radix Saposhnikoviae], 12 g; Conioselinum anthriscoides ‘Chuanxiong’[Apiaceae;Rhizoma Ligustici], 12 g; Epimedium sagittatum (Siebold & Zucc.) Maxim. [Berberidaceae;Herba Epimedii], 10 g; Rehmannia glutinosa (Gaertn.) DC. [Orobanchaceae;Radix Rehmanniae Preparata], 10 g; Psoralea fructus[Fabaceae; Fructus Psoraliae], 10 g	N	N
Liu et al. (2016)	Henan Provincial Chinese Medicine Hospital, Zhengzhou Henan, China	Forsythia suspensa (Thunb.) Vahl [Oleaceae; Fructus Forsythiae], 10 g; Phellodendron amurense Rupr. [Rutaceae;Cortex Phellodendri], 10 g; Paeonia lactiflora Pall.[Paeoniaceae;Radix Paeoniae Alba], 10 g; Stephania tetrandra[Menispermaceae; Radix Stephaniae Tetrandrae], 15 g; Coix lacryma-jobi L.[Poaceae;Semen Coicis], 15 g; Grona styracifolia (Osbeck) H.Ohashi & K.Ohashi [Fabaceae; Herba Lysimachiae], 10 g; Lonicera japonica [Caprifoliaceae;Flos Lonicerae], 10 g; Clematis chinensis Osbeck[Ranunculaceae;Radix Clematidis], 15 g; Viola philippica Cav. [Violaceae; Herba Violae], 10 g; Agkistrodon[Deinagkistrodon;Bungarus], 10 g; Patrinia scabiosifolia Link [Caprifoliaceae; Herba Patriniae], 10 g; Saposhnikovia divaricata (Turcz. ex Ledeb.) Schischk. [Apiaceae;Radix Saposhnikoviae], 10 g; Lonicera japonica Thunb. [Caprifoliaceae;Caulis Lonicerae], 10 g; Morus alba[Moraceae; Ramulus Mori], 10 g; Pheretima asiatica Michaelsen[Megascolecidae;Lumbricus], 6 g; Glycyrrhiza glabra[Fabaceae; Radix Glycyrrhizae], 6 g	N	N
Chen et al. (2016)	Jingfukang Pharmaceutical Co., Ltd.	Ant ; Panax ginseng C.A.Mey.[Araliaceae;Radix Ginseng]; Aconitum carmichaeli Debeaux [Ranunculaceae;Radix Aconiti]; Neolitsea cassia (L.) Kosterm.[Lauraceae;Ramulus Cinnamomi]; Atractylodes lancea (Thunb.) DC.[Asteraceae;Rhizoma Atractylodis]; Phellodendron amurense Rupr. [Rutaceae;Cortex Phellodendri] ; Coix lacryma-jobi L.[Poaceae;Semen Coicis] ; Alisma plantago-aquatica subsp. orientale (Sam.) Sam.[Alismataceae;Rhizoma Alismatis]; Centipede[Scolopendridae; Scolopendra] ; Zaocys dhumnades(Cantor)[Natricinae;Zaocys dhumnades] ; Lycopodium japonicum[Lycopodiaceae; Herba Lycopodii]; Strychnos nux-vomica L. [Loganiaceae; Semen Strychni]; Spatholobus suberectus Dunn[Fabaceae;Caulis Spatholobi]; Garden Balsam Stem [Euphorbiaceae; Speranskia tuberculata (Bunge) Baill]; Salvia miltiorrhiza Bunge[Lamiaceae; Radix Salviae Miltiorrhizae]	Y-Prepared according to *Chinese Pharmacopeia 2015*	N
Jiang and Wu (2016)	Nantong Hospital of Traditional Chinese Medicine, Jiangsu Province, China	Aconitum carmichaeli Debeaux [Ranunculaceae;Radix Aconiti], 8 g; Neolitsea cassia (L.) Kosterm.[Lauraceae;Ramulus Cinnamomi], 8 g; Angelica sinensis (Oliv.) Diels[Apiaceae;Radix Angelicae Sinensis], 10 g; Shengdi15 g; Paeonia lactiflora Pall.[Paeoniaceae;Radix Paeoniae Alba], 20 g; Anemarrhena asphodeloides[Liliaceae; Rhizoma Anemarrhenae], 20 g; Lonicera japonica Thunb. [Caprifoliaceae;Caulis Lonicerae], 20 g; Pheretima asiatica Michaelsen[Megascolecidae;Lumbricus], 12 g; Bombyx mori Linnaeus[Bombyx Linnaeus;Bombyx Batryticatus], 12 g; Zaocys dhumnades(Cantor)[Natricinae;Zaocys dhumnades], 10 g; Glycyrrhiza glabra[Fabaceae; Radix Glycyrrhizae], 6 g	N	N
Wang and Tu (2017)	Dazhou Central Hospital, Sichuan Province, China	Astragalus mongholicus Bunge[Fabaceae;Radix Astragali seu Hedysari], 60 g; Neolitsea cassia (L.) Kosterm.[Lauraceae;Ramulus Cinnamomi], 20 g; Paeonia lactiflora Pall.[Paeoniaceae;Radix Paeoniae Alba], 20 g; Zingiber officinale[Zingiberaceae; Rhizoma Zingiberis], 10 g; Ziziphus jujuba Mill. [Rhamnaceae; Fructus Jujubae], 10 g; Glycyrrhiza glabra[Fabaceae; Radix Glycyrrhizae], 5 g; Tripterygium wilfordii[Celastraceae; Radix Tripterygii Wilfordii], 15 g; Spatholobus suberectus Dunn[Fabaceae;Caulis Spatholobi], 30 g; Piper kadsura[Piperaceae; Caulis Piperis Kadsurae], 30 g; Trachelospermum jasminoides (Lindl.) Lem. [Apocynaceae; Caulis Trachelospermi], 30 g; Yinhuateng 30 g	N	N
Pang et al. (2017)	Affiliated Hospital of Shandong University of Chinese Medicine, Jinan, China	Angelica sinensis (Oliv.) Diels[Apiaceae;Radix Angelicae Sinensis], 20 g; Paeonia lactiflora Pall.[Paeoniaceae;Radix Paeoniae Alba], 30 g; Atractylodes macrocephala Koidz.[Asteraceae;Rhizoma Atractylodis Macrocephalae], 15 g; Atractylodes lancea (Thunb.) DC.[Asteraceae;Rhizoma Atractylodis],10 g; Sinomenium acutum[Menispermaceae; Caulis Sinomenii], 20 g; Sarcandra glabra (Thunb.) Nakai[Chloranthaceae;Herba Sarcandrae], 30 g; Conioselinum anthriscoides ‘Chuanxiong’[Apiaceae;Rhizoma Ligustici], 20 g; Saposhnikovia divaricata (Turcz. ex Ledeb.) Schischk. [Apiaceae;Radix Saposhnikoviae], 9 g; Glycyrrhiza glabra[Fabaceae; Radix Glycyrrhizae], 30 g	N	N
Li et al. (2017)	First Affiliated Hospital of Tianjin University of Traditional Chinese Medicine, Tianjin, China	Angelica dahurica (Hoffm.) Benth. & Hook.f. ex Franch. & Sav. [Apiaceae;Radix Angelicae Pubescentis], 15 g; Hansenia weberbaueriana (Fedde ex H.Wolff) Pimenov & Kljuykov [Apiaceae;Rhizoma et Radix Notopterygii], 15 g; Conioselinum anthriscoides ‘Chuanxiong’[Apiaceae;Rhizoma Ligustici], 15 g; Frankincense[Burseraceae; Olibanum], 10 g; Gentiana macrophylla Pall.[Gentianaceae;Radix Gentianae Macrophyllae], 10 g; Aucklandia costus Falc. [Asteraceae; Radix Aucklandiae], 10 g; Morus alba[Moraceae; Ramulus Mori], 10 g; Piper kadsura[Piperaceae; Caulis Piperis Kadsurae], 10 g; Angelica sinensis (Oliv.) Diels[Apiaceae;Radix Angelicae Sinensis], 20 g; Neolitsea cassia (L.) Kosterm.[Lauraceae;Ramulus Cinnamomi], 12 g; Glycyrrhiza glabra[Fabaceae; Radix Glycyrrhizae], 6 g	N	N
Gao and Bu, (2017)	The Second Traditional Chinese Medicine Hospital of Jiangsu, Nanjing Jiangsu, China	Rehmannia glutinosa (Gaertn.) DC. [Orobanchaceae;Radix Rehmanniae Preparata], 30 g; Astragalus mongholicus Bunge[Fabaceae;Radix Astragali seu Hedysari],n 15 g; Shanyu 20 g; Paeonia × suffruticosa [Paeoniaceae;Cortex Moutan Radicis], 10 g; Poria[Polyporaceae; Sclerotium Poriae Cocos], 15 g; Coptis chinensis Franch.[Ranunculaceae;Rhizoma Coptidis], 10 g; Dioscorea oppositifolia[Dioscoreaceae; Rhizoma Dioscoreae], 20 g; Chrysanthemum × morifolium (Ramat.) Hemsl.[Asteraceae;Flos Chrysanthemi], 10 g; Alisma plantago-aquatica subsp. orientale (Sam.) Sam.[Alismataceae;Rhizoma Alismatis], 10 g; Paeonia lactiflora Pall. [Paeoniaceae;Radix Paeoniae Rubra], 15 g; Angelica sinensis (Oliv.) Diels[Apiaceae;Radix Angelicae Sinensis], 10 g	N	N
Gao and Bu (2017)	Rheumatism Hospital of Shanxi Jinkang, Taiyuan, Chian	Ant ; Atractylodes macrocephala Koidz.[Asteraceae;Rhizoma Atractylodis Macrocephalae] ; Aconitum carmichaeli[Ranunculaceae; Radix Aconiti Lateralis Preparata] ; Neolitsea cassia (L.) Kosterm.[Lauraceae;Ramulus Cinnamomi]; etc.	N	N
Wang and Tu (2017)	Tongji Hospital Affiliated to Tongji Medical School of Huazhong University of Science and Technology, Wuhan Hubei, China	Aconitum carmichaeli Debeaux [Ranunculaceae;Radix Aconiti], 20 g; Ephedra sinica Stapf [Ephedraceae;Herba Ephedrae], 6 g; Astragalus mongholicus Bunge[Fabaceae;Radix Astragali seu Hedysari], 30 g; Paeonia lactiflora Pall.[Paeoniaceae;Radix Paeoniae Alba], 20 g; Glycyrrhiza glabra[Fabaceae; Radix Glycyrrhizae], 10 g; Neolitsea cassia (L.) Kosterm.[Lauraceae;Ramulus Cinnamomi], 10 g; Clematis chinensis Osbeck[Ranunculaceae;Radix Clematidis], 10 g; Asarum sieboldii[Aristolochiaceae; Herba cum Radix Asari], 3 g; Eupolyphaga[Corydidae;Eupolyphaga Seu Steleophaga], 10 g; Morus alba[Moraceae; Ramulus Mori], 20 g; Lycopodium japonicum[Lycopodiaceae; Herba Lycopodii], 20 g; Angelica dahurica (Hoffm.) Benth. & Hook.f. ex Franch. & Sav. [Apiaceae;Radix Angelicae Pubescentis], 20 g; Taxillus chinensis (DC.) Danser[Loranthaceae; Herba Taxilli], 20 g; Reynoutria multiflora (Thunb.) Moldenke[Polygonaceae;Radix Polygoni Multiflori], 20 g; Dipsacus asper[Caprifoliaceae; Radix Dipsaci], 15 g	N	N
Ma et al. (2018)	Lanzhou Traditional Chinese Medicine Orthopedics Hospital, Gansu Province, China	Paeonia lactiflora Pall. [Paeoniaceae;Radix Paeoniae Rubra], 15 g; Angelica sinensis (Oliv.) Diels[Apiaceae;Radix Angelicae Sinensis], 15 g; Pheretima asiatica Michaelsen[Megascolecidae;Lumbricus], 10 g; Astragalus mongholicus Bunge[Fabaceae;Radix Astragali seu Hedysari], 25 g; Conioselinum anthriscoides ‘Chuanxiong’[Apiaceae;Rhizoma Ligustici], 10 g; Arisaema erubescens[Araceae; Arisaemae cum Bile], 10 g; Sinapis alba L. [Brassicaceae;Semen sinapis.], 10 g; Buthus martensii Karsch[Scorpiones;Scorpio], 5 g; Angelica dahurica (Hoffm.) Benth. & Hook.f. ex Franch. & Sav. [Apiaceae;Radix Angelicae Pubescentis], 15 g; Hansenia weberbaueriana (Fedde ex H.Wolff) Pimenov & Kljuykov [Apiaceae;Rhizoma et Radix Notopterygii], 15 g; Neolitsea cassia (L.) Kosterm.[Lauraceae;Ramulus Cinnamomi], 10 g; Paeonia lactiflora Pall.[Paeoniaceae;Radix Paeoniae Alba], 15 g; Glycyrrhiza glabra[Fabaceae; Radix Glycyrrhizae], 5 g	N	N
Guo et al. (2018)	Affiliated Hospital of Gansu University of Traditional Chinese Medicine, Gansu Province, China	Cervus nippon Temminck [Cervidae; Cornu Cervi Degelatinatum] ; Equus asinus L. [Equidae; Colla Corii Asini] ; Buthus martensii Karsch[Scorpiones;Scorpio] ; Panax notoginseng (Burkill) F.H.Chen[Araliaceae;Radix Notoginseng]; Neolitsea cassia[Lauraceae; Cortex Cinnamomi] ; Coptis chinensis Franch.[Ranunculaceae;Rhizoma Coptidis] ; Ligustrum lucidum W.T.Aiton [Oleaceae; Fructus Ligustri Lucidi] ; Chinemys reevesii (Gray) [Testudinidae; Plastrum Testudinis] ; Trionyx sinensis Wiegmann [Trionychidae; Carapax Trionycis]; Drynaria roosii[Polypodiaceae; Rhizoma Drynariae] ; Spatholobus suberectus Dunn[Fabaceae;Caulis Spatholobi] ; Glycyrrhiza glabra[Fabaceae; Radix Glycyrrhizae]	N	N
Xu et al. (2018)	Kangmei Pharmaceutical Co., Ltd.	Lonicera japonica Thunb. [Caprifoliaceae;Caulis Lonicerae], 30 g; Angelica sinensis (Oliv.) Diels[Apiaceae;Radix Angelicae Sinensis], 30 g; Scrophularia ningpoensis[Scrophulariaceae; Radix Scrophulariae], 20 g; Glycyrrhiza glabra[Fabaceae; Radix Glycyrrhizae], 10 g; Dioscorea nipponica Makino[Dioscoreaceae;Rhizoma Dioscoreae Nipponicae], 30 g; Arctium lappa L. [Arctium lappa L.; Fructus Arctii], 15 g; Bombyx mori Linnaeus[Bombyx Linnaeus;Bombyx Batryticatus], 10 g; etc.	N	N
Bian and Wang (2018)	Guangdong Shengkang Pharmaceutical Co., Ltd.	Astragalus mongholicus Bunge[Fabaceae;Radix Astragali seu Hedysari], 30 g; Neolitsea cassia (L.) Kosterm.[Lauraceae;Ramulus Cinnamomi], 15 g; Paeonia lactiflora Pall. [Paeoniaceae;Radix Paeoniae Rubra], 15 g; Paeonia lactiflora Pall.[Paeoniaceae;Radix Paeoniae Alba], 15 g; Angelica sinensis (Oliv.) Diels[Apiaceae;Radix Angelicae Sinensis], 20 g; Arisaema erubescens[Araceae; Arisaemae cum Bile], 15 g; Zingiber officinale[Zingiberaceae; Rhizoma Zingiberis],10 g; Bombyx mori Linnaeus[Bombyx Linnaeus;Bombyx Batryticatus], 15 g; Cremastra appendiculata (D.Don) Makino[Orchidaceae;Pseudobulbus Cremastrae seu Pleiones], 15 g; Sinapis alba L. [Brassicaceae;Semen sinapis.], 9 g; Corydalis yanhusuo[Papaveraceae;Rhizoma Corydalis], 15 g; Pheretima asiatica Michaelsen[Megascolecidae;Lumbricus], 15 g; Lonicera japonica Thunb. [Caprifoliaceae;Caulis Lonicerae], 30 g; Glycyrrhiza glabra[Fabaceae; Radix Glycyrrhizae], 6 g	N	N
Shi et al. (2018)	Tongling City Hospital of Traditional Chinese Medicine, Shanxi Province, China	Damp-heat obstruction type: Gentiana macrophylla Pall.[Gentianaceae;Radix Gentianae Macrophyllae], 15 g; Angelica dahurica (Hoffm.) Benth. & Hook.f. ex Franch. & Sav. [Apiaceae;Radix Angelicae Pubescentis], 15 g; Stephania tetrandra[Menispermaceae; Radix Stephaniae Tetrandrae], 9 g; Poria[Polyporaceae; Sclerotium Poriae Cocos], 15 g; Coix lacryma-jobi L.[Poaceae;Semen Coicis], 30 g; Lycium barbarum L. [Lycium barbarum L.; Lycii Cortex], 15 g; Lonicera japonica Thunb. [Caprifoliaceae;Caulis Lonicerae], 30 g; Dipsacus asper[Caprifoliaceae; Radix Dipsaci], 15 g; Drynaria roosii[Polypodiaceae; Rhizoma Drynariae], 15 g; Anemarrhena asphodeloides[Liliaceae; Rhizoma Anemarrhenae], 15 g; Phellodendron amurense Rupr. [Rutaceae;Cortex Phellodendri], 15 g; Frankincense[Burseraceae; Olibanum], 9 g; Glycyrrhiza glabra[Fabaceae; Radix Glycyrrhizae], 6 g. Kidney qi deficiency and cold type: Epimedium sagittatum (Siebold & Zucc.) Maxim. [Berberidaceae;Herba Epimedii], 15 g; Curculigo orchioides Gaertn.[Hypoxidaceae;Rhizoma Curculigins], 12 g; Angelica dahurica (Hoffm.) Benth. & Hook.f. ex Franch. & Sav. [Apiaceae;Radix Angelicae Pubescentis], 15 g; Hansenia weberbaueriana (Fedde ex H.Wolff) Pimenov & Kljuykov [Apiaceae;Rhizoma et Radix Notopterygii], 9 g; Neolitsea cassia (L.) Kosterm.[Lauraceae;Ramulus Cinnamomi], 15 g; Dipsacus asper[Caprifoliaceae; Radix Dipsaci], 15 g; Drynaria roosii[Polypodiaceae; Rhizoma Drynariae], 15 g; Clematis chinensis Osbeck[Ranunculaceae;Radix Clematidis], 15 g; Psoralea fructus[Fabaceae; Fructus Psoraliae], 15 g; Achyranthes bidentata Blume [Amaranthaceae;Radix Achyranthis Bidentatae], 15 g; Lycopodium japonicum[Lycopodiaceae; Herba Lycopodii], 30 g; Saposhnikovia divaricata (Turcz. ex Ledeb.) Schischk. [Apiaceae;Radix Saposhnikoviae], 10 g; Aconitum carmichaeli[Ranunculaceae; Radix Aconiti Lateralis Preparata], 9 g; Glycyrrhiza glabra[Fabaceae; Radix Glycyrrhizae], 6g. Blood stasis type: Eucommia ulmoides Oliv. [Eucommiaceae; Cortex Eucommiae], 15 g; Dipsacus asper[Caprifoliaceae; Radix Dipsaci], 15 g; Deerhorn glue[Cervidae;Colla Corni Cervi], 10 g; Cyperus rotundus L. [Cyperaceae; Rhizoma Cyperi], 15 g; Psoralea fructus[Fabaceae; Fructus Psoraliae], 15 g; Drynaria roosii[Polypodiaceae; Rhizoma Drynariae], 15 g; Salvia miltiorrhiza Bunge[Lamiaceae; Radix Salviae Miltiorrhizae], 20 g; Reynoutria multiflora (Thunb.) Moldenke[Polygonaceae;Radix Polygoni Multiflori], 15 g; Frankincense[Burseraceae; Olibanum], 9 g; Commiphora myrrha[Burseraceae; Resina Commiphorae], 9 g; Angelica dahurica (Hoffm.) Benth. & Hook.f. ex Franch. & Sav. [Apiaceae;Radix Angelicae Pubescentis], 15 g; Spatholobus suberectus Dunn[Fabaceae;Caulis Spatholobi], 30 g; Achyranthes bidentata Blume [Amaranthaceae;Radix Achyranthis Bidentatae], 15 g; Angelica sinensis (Oliv.) Diels[Apiaceae;Radix Angelicae Sinensis], 15 g; Paeonia lactiflora Pall. [Paeoniaceae;Radix Paeoniae Rubra], 12 g	N	N
Ge et al. (2018)	Jiaozuo Traditional Chinese Medicine Hospital, Henan Province, China	Piper kadsura[Piperaceae; Caulis Piperis Kadsurae], 12 g; Sinomenium acutum[Menispermaceae; Caulis Sinomenii], 12 g; Spatholobus suberectus Dunn[Fabaceae;Caulis Spatholobi], 12 g; Yinhuateng 12 g; Saposhnikovia divaricata (Turcz. ex Ledeb.) Schischk. [Apiaceae;Radix Saposhnikoviae], 10 g; Dioscorea nipponica Makino[Dioscoreaceae;Rhizoma Dioscoreae Nipponicae], 10 g; Sinapis alba L. [Brassicaceae;Semen sinapis.], 10 g; Buthus martensii Karsch[Scorpiones;Scorpio], 6 g; Angelica sinensis (Oliv.) Diels[Apiaceae;Radix Angelicae Sinensis], 15 g; Arisaema erubescens[Araceae; Arisaemae cum Bile], 10 g; Conioselinum anthriscoides ‘Chuanxiong’[Apiaceae;Rhizoma Ligustici], 8 g; Paeonia lactiflora Pall.[Paeoniaceae;Radix Paeoniae Alba], 10 g; Prunus persica (L.) Batsch[Rosaceae;Semen Persicae], 9 g; Carthamus tinctorius[Asteraceae; Flos Carthami], 6 g	N	N
Zeng and Chen (2018)	Liaoning Good Nurse Pharmaceutical Co., Ltd.	Rehmannia glutinosa (Gaertn.) DC. [Orobanchaceae;Radix Rehmanniae Preparata] ; Epimedium sagittatum (Siebold & Zucc.) Maxim. [Berberidaceae;Herba Epimedii] ; Clematis chinensis Osbeck[Ranunculaceae;Radix Clematidis] ; Cibotium barometz (L.) J.Sm.[Cyatheaceae;Rhizoma Cibotii] ; Anemarrhena asphodeloides[Liliaceae; Rhizoma Anemarrhenae] ; Lycopodium japonicum[Lycopodiaceae; Herba Lycopodii] ; Drynaria roosii[Polypodiaceae; Rhizoma Drynariae]; etc.	Y-Prepared according to *Chinese Pharmacopeia 2015*	N
Hu (2018)	Neijiang Hospita of Traditional Chinese Medicine, Sichuan Neijiang, China	Wind-cold-dampness syndrome: Clematis chinensis Osbeck[Ranunculaceae;Radix Clematidis], 15 g; Hansenia weberbaueriana (Fedde ex H.Wolff) Pimenov & Kljuykov [Apiaceae;Rhizoma et Radix Notopterygii], 9 g; Neolitsea cassia (L.) Kosterm.[Lauraceae;Ramulus Cinnamomi], 9 g; Commiphora myrrha[Burseraceae; Resina Commiphorae], 9 g; Conioselinum anthriscoides ‘Chuanxiong’[Apiaceae;Rhizoma Ligustici], 9 g; Gentiana macrophylla Pall.[Gentianaceae;Radix Gentianae Macrophyllae], 9 g; Angelica dahurica (Hoffm.) Benth. & Hook.f. ex Franch. & Sav. [Apiaceae;Radix Angelicae Pubescentis], 9 g; Frankincense[Burseraceae; Olibanum], 9 g; Angelica sinensis (Oliv.) Diels[Apiaceae;Radix Angelicae Sinensis], 9g. Wind-heat-dampness syndrome: Morus alba[Moraceae; Ramulus Mori], 20 g; Lonicera japonica Thunb. [Caprifoliaceae;Caulis Lonicerae], 20 g; Paeonia lactiflora Pall.[Paeoniaceae;Radix Paeoniae Alba], 15 g; Stephania tetrandra[Menispermaceae; Radix Stephaniae Tetrandrae], 12 g; Erythrina indica Lam. [Leguminosae; Cortex Erythrinae] , 12 g; Anemarrhena asphodeloides[Liliaceae; Rhizoma Anemarrhenae], 10 g; Saposhnikovia divaricata (Turcz. ex Ledeb.) Schischk. [Apiaceae;Radix Saposhnikoviae], 9 g; Neolitsea cassia (L.) Kosterm.[Lauraceae;Ramulus Cinnamomi], 6g. of Phlegm and blood stasis syndrome: Codonopsis pilosula[Campanulaceae; Radix Codonopsis], 15 g; Taxillus chinensis (DC.) Danser[Loranthaceae; Herba Taxilli], 12 g; Gentiana macrophylla Pall.[Gentianaceae;Radix Gentianae Macrophyllae], 12 g; Rehmannia glutinosa (Gaertn.) DC. [Orobanchaceae;Radix Rehmanniae Preparata], 12 g; Poria[Polyporaceae; Sclerotium Poriae Cocos], 12 g; Paeonia lactiflora Pall.[Paeoniaceae;Radix Paeoniae Alba], 12 g; Eucommia ulmoides Oliv. [Eucommiaceae; Cortex Eucommiae], 10 g; Angelica sinensis (Oliv.) Diels[Apiaceae;Radix Angelicae Sinensis], 10 g; Achyranthes bidentata Blume [Amaranthaceae;Radix Achyranthis Bidentatae], 10 g; Conioselinum anthriscoides ‘Chuanxiong’[Apiaceae;Rhizoma Ligustici], 10 g; Angelica dahurica (Hoffm.) Benth. & Hook.f. ex Franch. & Sav. [Apiaceae;Radix Angelicae Pubescentis], 9 g; Saposhnikovia divaricata (Turcz. ex Ledeb.) Schischk. [Apiaceae;Radix Saposhnikoviae], 9 g; Neolitsea cassia[Lauraceae; Cortex Cinnamomi], 3 g; Asarum sieboldii[Aristolochiaceae; Herba cum Radix Asari], 3g. Kidney-Yang deficiency syndrome: Wooly datvhmanspipe herb [Aristolochiaceae;Herba Aristolochiae Mollissimae], 15 g; Atractylodes macrocephala Koidz.[Asteraceae;Rhizoma Atractylodis Macrocephalae], 15 g; Epimedium sagittatum (Siebold & Zucc.) Maxim. [Berberidaceae;Herba Epimedii], 15 g; Clematis chinensis Osbeck[Ranunculaceae;Radix Clematidis], 15 g; Gynochthodes officinalis (F.C.How) Razafim. [Rubiaceae; Radix Morindae Officinalis], 12 g; Achyranthes bidentata Blume [Amaranthaceae;Radix Achyranthis Bidentatae], 12 g; Dioscorea oppositifolia[Dioscoreaceae; Rhizoma Dioscoreae], 12 g; Poria[Polyporaceae; Sclerotium Poriae Cocos], 12 g; Cibotium barometz (L.) J.Sm.[Cyatheaceae;Rhizoma Cibotii], 12 g; Aconitum carmichaeli[Ranunculaceae; Radix Aconiti Lateralis Preparata], 9 g; Shanyu 9 g; Neolitsea cassia (L.) Kosterm.[Lauraceae;Ramulus Cinnamomi], 9 g	N	N
Yuan et al. (2019)	The First Affiliated Hospital of Guangxi University of Chinese Medicine, Nanning Guangxi, China	Gypsum [Mineral; Gypsum Fibrosum], 30 g; Anemarrhena asphodeloides[Liliaceae; Rhizoma Anemarrhenae], 20 g; Non-glutinous rice [Gramineae; Oryza sativa L.], 20 g; Neolitsea cassia (L.) Kosterm.[Lauraceae;Ramulus Cinnamomi], 6 g; Glycyrrhiza glabra[Fabaceae; Radix Glycyrrhizae], 6 g; Angelica sinensis (Oliv.) Diels[Apiaceae;Radix Angelicae Sinensis], 10 g; Rehmannia glutinosa[Orobanchaceae; Radix Rehmanniae], 50 g; Prunus armeniaca L. [Rosaceae; Semen Armeniacae Amarum], 12 g; Coix lacryma-jobi L.[Poaceae;Semen Coicis], 12 g; Clematis chinensis Osbeck[Ranunculaceae;Radix Clematidis], 12 g; Arnebia euchroma (Royle ex Benth.) I.M.Johnst. [Boraginaceae; Radix Lithospermi], 30 g; Paeonia lactiflora Pall. [Paeoniaceae;Radix Paeoniae Rubra], 30 g;	N	N
Pang et al. (2019)	Zhongshan Hospital of Traditional Chinese Medicine Affiliated to Guangzhou University of Chinese Medicine, Guangdong Zhongshan, China	Neolitsea cassia (L.) Kosterm.[Lauraceae;Ramulus Cinnamomi] ; Tripterygium hypoglaucum (H.Lév.) Hutch. [Celastraceae; Tripterygium hypoglaucum(Devl.)Hutch.] ; Paeonia lactiflora Pall.[Paeoniaceae;Radix Paeoniae Alba] ; Spatholobus suberectus Dunn[Fabaceae;Caulis Spatholobi] ; Zaocys dhumnades(Cantor)[Natricinae;Zaocys dhumnades] ; Coix lacryma-jobi L.[Poaceae;Semen Coicis] ; Zingiber officinale[Zingiberaceae; Rhizoma Zingiberis]; etc.	N	N
Zhao (2019)	Shenzhen Second People's Hospital, Guangdong Shenzhen, China	Paeonia lactiflora Pall.[Paeoniaceae;Radix Paeoniae Alba], 12 g; Anemarrhena asphodeloides[Liliaceae; Rhizoma Anemarrhenae], 12 g; Neolitsea cassia (L.) Kosterm.[Lauraceae;Ramulus Cinnamomi], 9 g; Paeonia lactiflora Pall. [Paeoniaceae;Radix Paeoniae Rubra], 9 g; Ephedra sinica Stapf [Ephedraceae;Herba Ephedrae], 9 g; Atractylodes macrocephala Koidz.[Asteraceae;Rhizoma Atractylodis Macrocephalae], 9 g; Saposhnikovia divaricata (Turcz. ex Ledeb.) Schischk. [Apiaceae;Radix Saposhnikoviae], 9 g; Glycyrrhiza glabra[Fabaceae; Radix Glycyrrhizae], 6 g; Aconitum carmichaeli[Ranunculaceae; Radix Aconiti Lateralis Preparata], 6 g; Zingiber officinale[Zingiberaceae; Rhizoma Zingiberis], 3 pieces	N	N
Li et al. (2019)	Kangmei Pharmaceutical Co., Ltd.	Neolitsea cassia (L.) Kosterm.[Lauraceae;Ramulus Cinnamomi], 15 g; Anemarrhena asphodeloides[Liliaceae; Rhizoma Anemarrhenae], 10-15 g; Paeonia lactiflora Pall.[Paeoniaceae;Radix Paeoniae Alba], 10–30 g; Ephedra sinica Stapf [Ephedraceae;Herba Ephedrae], 10 g; Zingiber officinale[Zingiberaceae; Rhizoma Zingiberis],10 g; Saposhnikovia divaricata (Turcz. ex Ledeb.) Schischk. [Apiaceae;Radix Saposhnikoviae], 10 g; Atractylodes macrocephala Koidz.[Asteraceae;Rhizoma Atractylodis Macrocephalae], 10 g; Aconitum carmichaeli[Ranunculaceae; Radix Aconiti Lateralis Preparata], 10-30 g; Glycyrrhiza glabra[Fabaceae; Radix Glycyrrhizae], 10 g	N	N
Song et al. (2019)	Qinghai Hospital of Traditional Chinese Medicine, Qinghai Xining, China	Rehmannia glutinosa (Gaertn.) DC. [Orobanchaceae;Radix Rehmanniae Preparata], 30 g; Angelica dahurica (Hoffm.) Benth. & Hook.f. ex Franch. & Sav. [Apiaceae;Radix Angelicae Pubescentis], 15 g; Paeonia lactiflora Pall.[Paeoniaceae;Radix Paeoniae Alba], 15 g; Angelica sinensis (Oliv.) Diels[Apiaceae;Radix Angelicae Sinensis], 12 g; Neolitsea cassia[Lauraceae; Cortex Cinnamomi], 10 g; Deerhorn glue[Cervidae;Colla Corni Cervi], 10 g; Sinapis alba L. [Brassicaceae;Semen sinapis.], 10 g; Ephedra sinica Stapf [Ephedraceae;Herba Ephedrae], 10 g; Glycyrrhiza glabra[Fabaceae; Radix Glycyrrhizae], 9 g; Poria[Polyporaceae; Sclerotium Poriae Cocos], 9 g	N	N
Zhang et al. (2019)	Luoyang Zhenggu Hospital (Henan Orthopaedic Hospital), Zhengzhou, China	Coix lacryma-jobi L.[Poaceae;Semen Coicis], 30 g; Angelica sinensis (Oliv.) Diels[Apiaceae;Radix Angelicae Sinensis], 12 g; Saposhnikovia divaricata (Turcz. ex Ledeb.) Schischk. [Apiaceae;Radix Saposhnikoviae], 10 g; Conioselinum anthriscoides ‘Chuanxiong’[Apiaceae;Rhizoma Ligustici], 10 g; Ephedra sinica Stapf [Ephedraceae;Herba Ephedrae], 10 g; Atractylodes lancea (Thunb.) DC.[Asteraceae;Rhizoma Atractylodis],10 g; Glycyrrhiza glabra[Fabaceae; Radix Glycyrrhizae], 10 g; Hansenia weberbaueriana (Fedde ex H.Wolff) Pimenov & Kljuykov [Apiaceae;Rhizoma et Radix Notopterygii], 5 g; Angelica dahurica (Hoffm.) Benth. & Hook.f. ex Franch. & Sav. [Apiaceae;Radix Angelicae Pubescentis], 5 g; Neolitsea cassia (L.) Kosterm.[Lauraceae;Ramulus Cinnamomi], 5 g	N	N
Fang et al. (2019)	Taihe Hospital of Shiyan City, Affiliated Hospital of Hubei Medical College, Shiyan Hubei, China	Astragalus mongholicus Bunge[Fabaceae;Radix Astragali seu Hedysari], 15 g; Conioselinum anthriscoides ‘Chuanxiong’[Apiaceae;Rhizoma Ligustici], 15 g; Aconitum carmichaeli[Ranunculaceae; Radix Aconiti Lateralis Preparata], 15 g; Zaocys dhumnades(Cantor)[Natricinae;Zaocys dhumnades], 15 g; Paeonia lactiflora Pall.[Paeoniaceae;Radix Paeoniae Alba], 10 g; Zingiber officinale Roscoe [Zingiberaceae; Rhizoma Zingiberis], 10 g; Neolitsea cassia (L.) Kosterm.[Lauraceae;Ramulus Cinnamomi], 10 g; Aconitum carmichaeli Debeaux [Ranunculaceae;Radix Aconiti], 10 g; Atractylodes macrocephala Koidz.[Asteraceae;Rhizoma Atractylodis Macrocephalae], 10 g; Angelica sinensis (Oliv.) Diels[Apiaceae;Radix Angelicae Sinensis], 10 g; Glycyrrhiza glabra[Fabaceae; Radix Glycyrrhizae], 6 g; Ephedra sinica Stapf [Ephedraceae;Herba Ephedrae], 9 g; Asarum sieboldii[Aristolochiaceae; Herba cum Radix Asari], 5 g; Centipede[Scolopendridae; Scolopendra], 1 piece; Buthus martensii Karsch[Scorpiones;Scorpio], 1 piece; Spatholobus suberectus Dunn[Fabaceae;Caulis Spatholobi], 30 g	N	N
Cao et al. (2020)	Affiliated Hospital of Liaoning University of Traditional Chinese Medicine, Shenyang, China	Angelica dahurica (Hoffm.) Benth. & Hook.f. ex Franch. & Sav. [Apiaceae;Radix Angelicae Pubescentis], 9 g; Taxillus chinensis (DC.) Danser[Loranthaceae; Herba Taxilli], 9 g; Eucommia ulmoides Oliv. [Eucommiaceae; Cortex Eucommiae], 9 g; Achyranthes bidentata Blume [Amaranthaceae;Radix Achyranthis Bidentatae], 9 g; Gentiana macrophylla Pall.[Gentianaceae;Radix Gentianae Macrophyllae], 9 g; Neolitsea cassia[Lauraceae; Cortex Cinnamomi], 9 g; Saposhnikovia divaricata (Turcz. ex Ledeb.) Schischk. [Apiaceae;Radix Saposhnikoviae], 9 g; Conioselinum anthriscoides ‘Chuanxiong’[Apiaceae;Rhizoma Ligustici], 9 g; Angelica sinensis (Oliv.) Diels[Apiaceae;Radix Angelicae Sinensis], 9 g; Paeonia lactiflora Pall.[Paeoniaceae;Radix Paeoniae Alba], 9 g; Asarum sieboldii[Aristolochiaceae; Herba cum Radix Asari], 5 g; Rehmannia glutinosa[Orobanchaceae; Radix Rehmanniae], 9 g; Hansenia weberbaueriana (Fedde ex H.Wolff) Pimenov & Kljuykov [Apiaceae;Rhizoma et Radix Notopterygii], 9 g; Neolitsea cassia (L.) Kosterm.[Lauraceae;Ramulus Cinnamomi], 9 g	N	N
Li (2020)	Zhengzhou Hospital of Traditional Chinese Medicine, Zhengzhou, China	Cremastra appendiculata (D.Don) Makino[Orchidaceae;Pseudobulbus Cremastrae seu Pleiones], 10 g; Sinapis alba L. [Brassicaceae;Semen sinapis.], 10 g; Bombyx mori Linnaeus[Bombyx Linnaeus;Bombyx Batryticatus], 10 g; Pheretima asiatica Michaelsen[Megascolecidae;Lumbricus], 10 g; Arisaema erubescens[Araceae; Arisaemae cum Bile], 15 g	N	N
Jiang and Ma (2020)	Jingzhou Hospital of Traditional Chinese Medicine, Jingzhou Hubei, China	Dioscorea nipponica Makino[Dioscoreaceae;Rhizoma Dioscoreae Nipponicae], 20 g; Sinomenium acutum[Menispermaceae; Caulis Sinomenii], 30 g; Sinapis alba L. [Brassicaceae;Semen sinapis.], 10 g; Paeonia lactiflora Pall. [Paeoniaceae;Radix Paeoniae Rubra], 10 g; Bolbostemma paniculatum (Maxim.) Franquet [Cucurbitaceae;Rhizoma Bolbostematis ], 8 g; Curcuma kwangsiensis S.G.Lee & C.F.Liang [Zingiberaceae; Rhizoma Curcumae], 6 g	N	N

### Quality of the Included Studies

**FIGURE 2:**
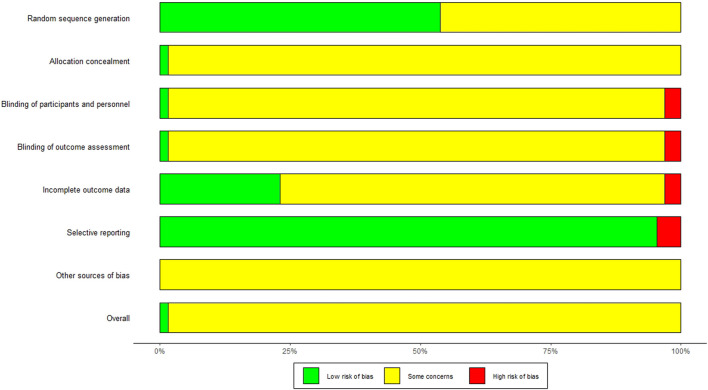
Evaluation for bias risk of included studies.

The quality of the trials was assessed and graded according to the criteria described in The Cochrane Handbook 4.2.6. Overall, for most of the included studies, the risk of bias was unclear. In addition, although the random design was mentioned in all studies, only 53.85% (35/65) of the studies described specific methods of random sequence generation. The 35 studies all used the simple random grouping method, regarding random sequence generation of which 32 studies used the random number table method and the other 3 studies selected by draw lots. Except for the study of Zhou (Zhou et al., 2010), the use of allocation concealment was not mentioned in the rest literature. Only one trial (Jiang and Wu, 2016) mentioned the double-blind of patients and personnel, and two studies (Huang et al., 2006; Xu et al., 2018) explicitly mentions the absence of the use of blinding, while the rest literature did not mention the use of blindness. There were 14 studies (Huang et al., 2006; Ma et al., 2009; Li et al., 2012a; He and Xiao, 2014; Li et al., 2015a; Li et al., 2015b; Shu et al., 2015; Chen et al., 2016; Li et al., 2017; Bian and Wang, 2018; Xu et al., 2018; Fang et al., 2019; Li et al., 2019; Pang et al., 2019) described the dropouts, and two trials (Li, 2006; Zhang and Chen, 2014) were not reported the number of people and the reasons for the failure at follow-up. Pre-designed outcomes were reported in most studies, detecting a low risk of reporting bias. The review authors’ judgments about each methodological quality item were presented as percentages across all the included studies in Figure 2.

### Effects of Interventions

#### Primary Outcomes

##### Serum RF Level After Treatment

**FIGURE 3:**
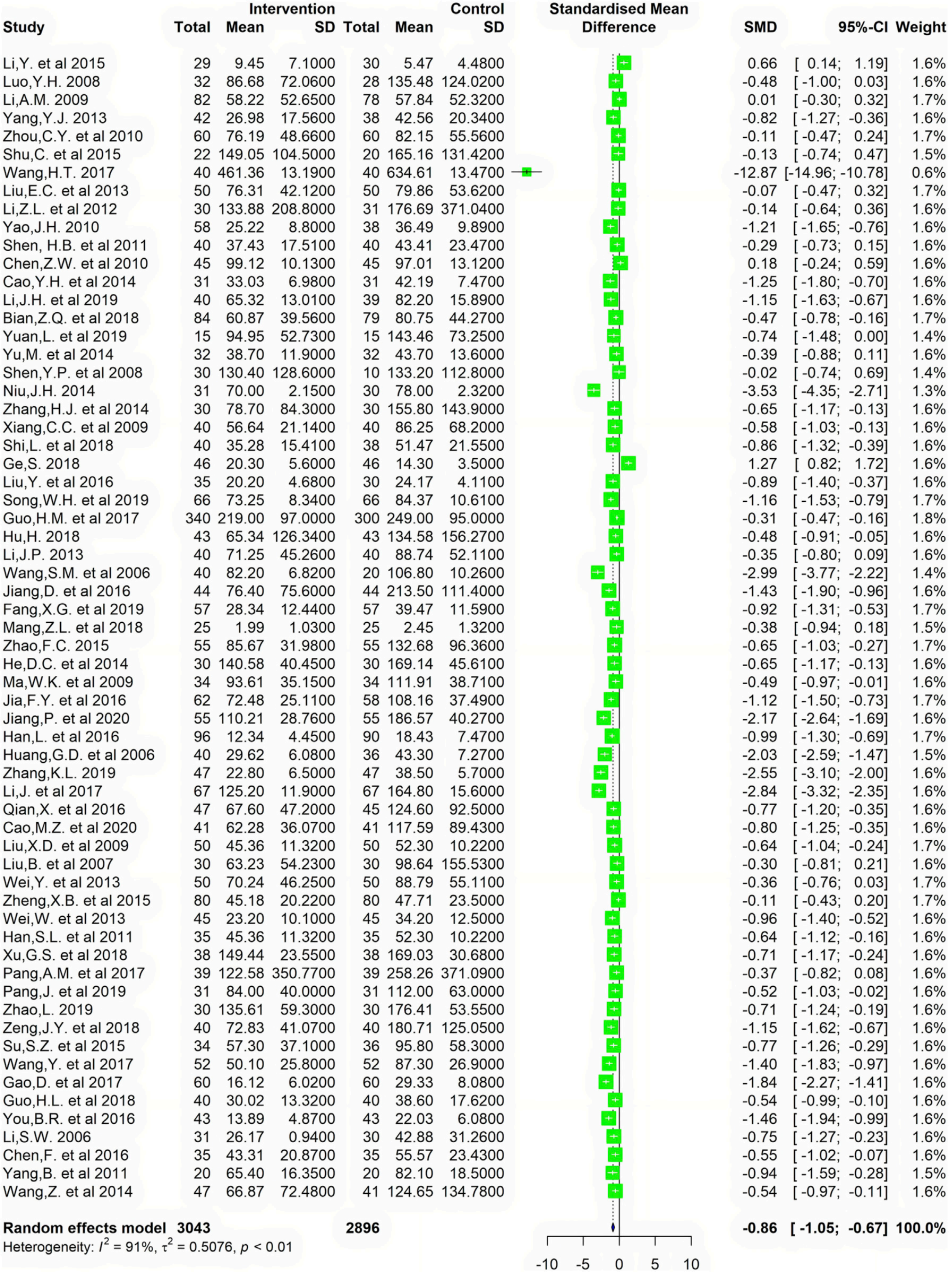
Forest plot and meta-analysis of RF.

Sixty-three studies including 5,939 patients (3,043 in the experimental group and 2,896 in the control group) provided the serum RF concentration data. The SMD of the RF levels in treatment group (CMC involved) between baseline and follow-up was 2.64 and 95%CI was [2.25, 3.02], while the SMD in control group (no CMC involved) between baseline and follow-up was 2.08 and 95%CI was [1.75, 2.40] (Supplementary Material 2). After treatment, the results showed that CMC had a significant difference in reducing serum RF level compared with the control group. (SMD = -0.86, 95%CI = [−1.05, −0.67]) (Figure 3).

##### Serum Anti-CCP Level After Treatment

**FIGURE 4:**
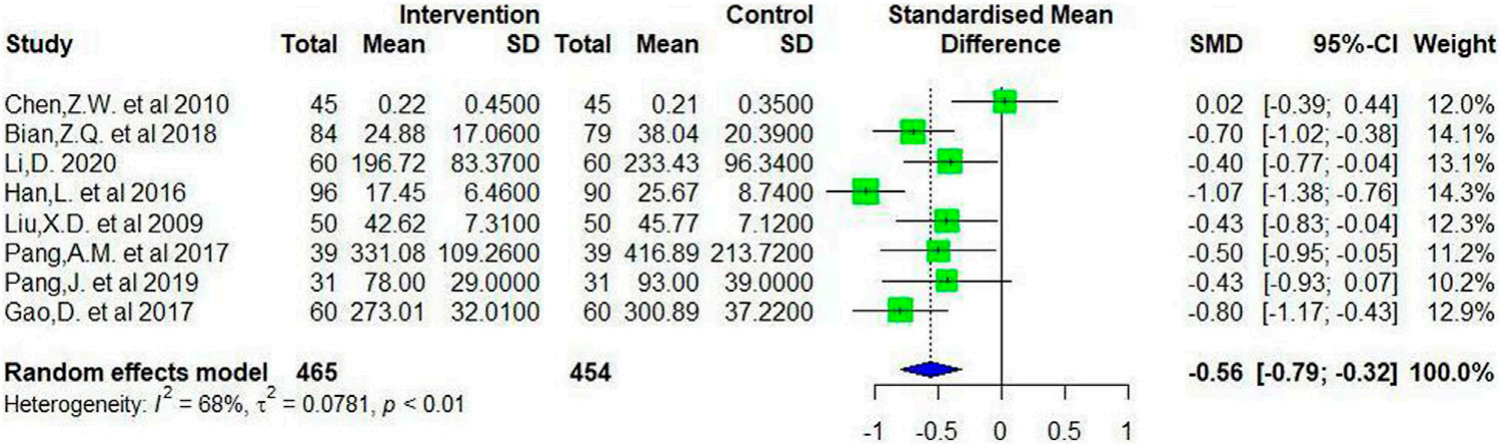
Forest plot and meta-analysis of anti-CCP.

Eight studies have observed changes in serum anti-CCP level before and after treatment and provided relevant data. The SMD of the anti-CCP levels in treatment group (CMC involved) between baseline and follow-up was 2.15 and 95%CI was [1.28, 3.01], while the SMD in control group (no CMC involved) between baseline and follow-up was 1.62 and 95%CI was [0.91,2.33] (Supplementary Material 3). Since a significant heterogeneity was detected [*I* (Zeng et al., 2013) = 68%, *p* < 0.01], the randomized effect model was adopted for analysis and the results indicated that CMC could significantly reduce the level of anti-CCP after treatment (SMD = −0.56, 95%CI = [−0.79, −0.32]) (Figure 4).

#### Secondary Outcomes

##### ACR Response

**FIGURE 5:**
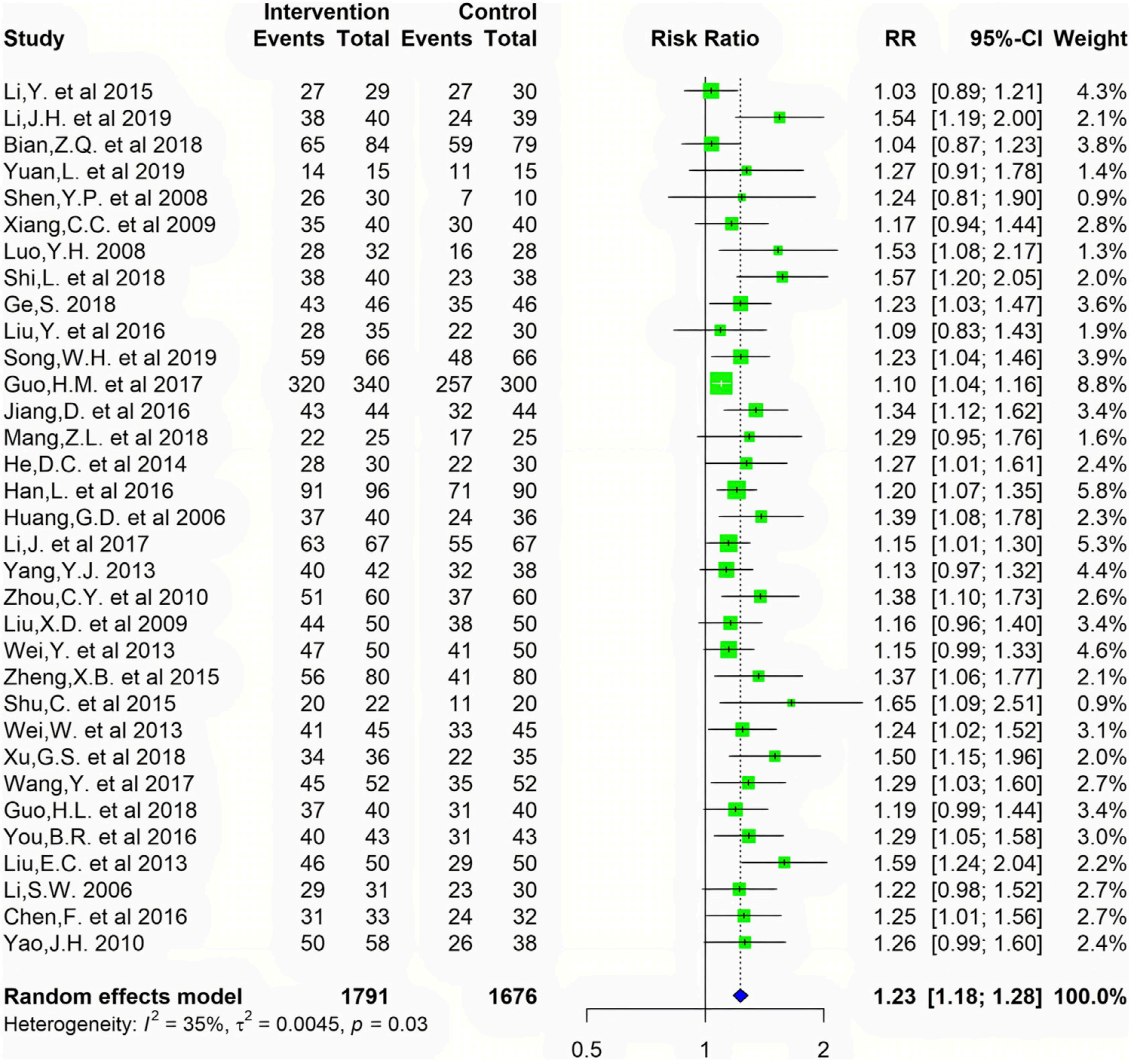
Forest plot and meta-analysis of ACR response.

A total of 14 studies reported ACR20 response rates over the treatment time, and 11 of them also reported the proportion of patients who achieved ACR50 and ACR70 response after treatment. The *I*-squared was 76% and the *p*-value was <0.01, so a random-effects model was adopted for the meta-analysis of ACR20 response. The results of the pooled analysis suggest that the proportion of RA patients who achieved an ACR20 response after CMC treatment was superior to that of the control group. (SMD = 1.20, 95%CI = [1.08, 1.33]) (Figure 5).

The low risk of heterogeneity was detected among all studies in terms of the ACR50 and ACR70 response of CMC therapy in RA patients. The analysis was performed using a fixed-effects model and the results demonstrated that the RR of achieving ACR50 was 1.50 [1.38, 1.78] and correspondingly the RR of achieving ACR70 was 2.12 [1.65, 2.72] (Figure 5).

##### Clinical Efficacy of TCM Symptoms

**FIGURE 6:**
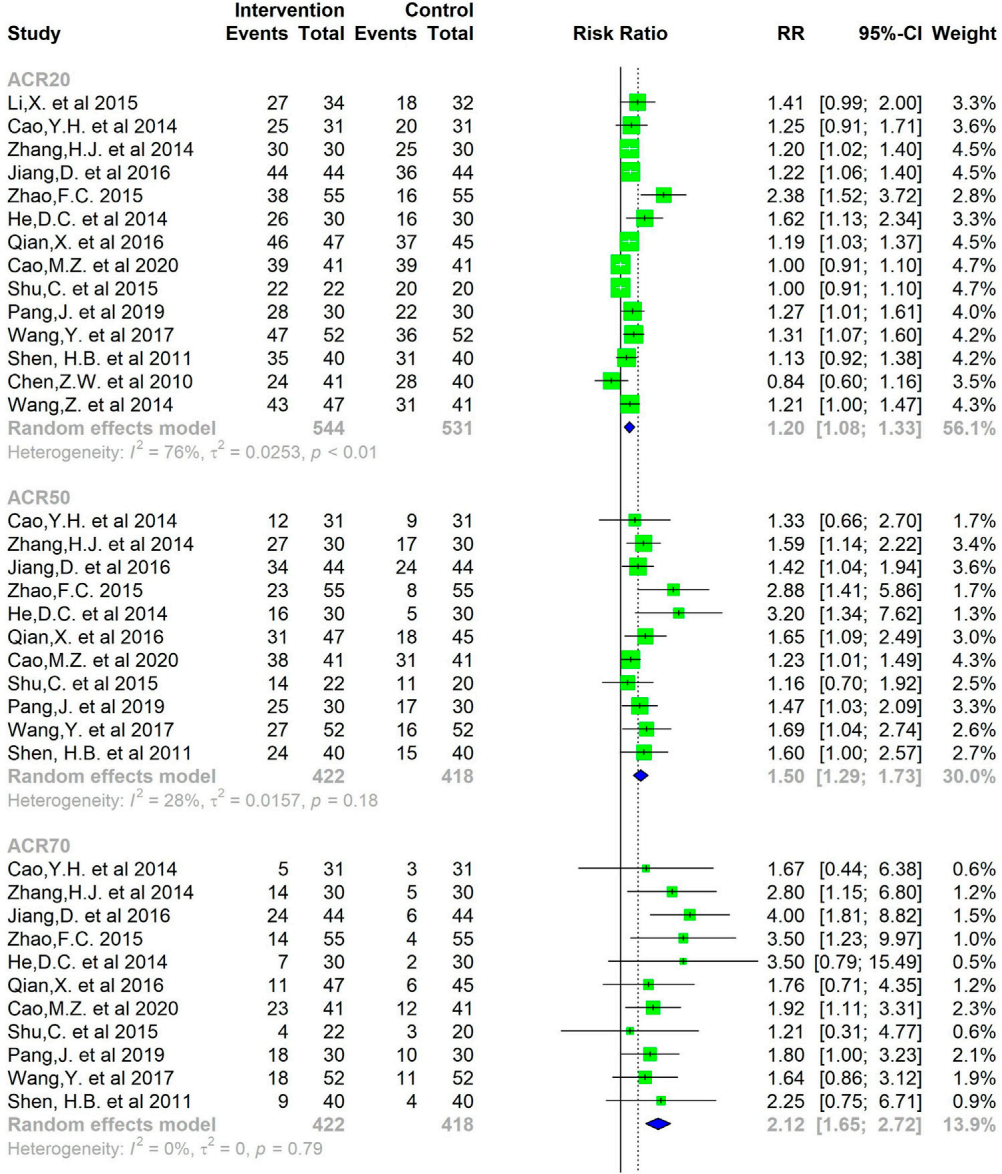
Forest plot and meta-analysis of clinical efficacy of TCM syndromes.

Data on the clinical efficacy of TCM symptoms were available for 33 studies containing a total of 3,467 cases. The meta-analysis yielded a pooled RR of 1.23 (*I* ^*2*^ = 35%, 95% CI 1.18–1.28). As shown in Figure 6, CMC has a good effect on patients with RA.

### Sensitivity and Subgroup Analysis

Through sensitivity analysis, after excluding different studies in turn, it was found that there was no change in the significance of any of the outcomes. However, in the sensitivity analysis of the primary outcomes, anti-CCP, it was found that *I*-squared dropped to 43.4% when a study (Han et al., 2016) was excluded. Considering the difference in the average age of the included patients, unlike other studies, this study included elderly patients with RA, aged ≥60 years old. When analyzing the response rate of TCM syndromes, it was found that after a study (Guo et al., 2017) were excluded, *I*-squared could be reduced to 11.9%. By reviewing the original article, it was found that the sample size of this study was large compared with other studies (total sample size >600), suggesting that the source of heterogeneity may be caused by the difference in sample size between studies.

After the heterogeneity test, we conducted a subgroup analysis to explore the source of heterogeneity when the heterogeneity between included studies was found to be significant and not negligible. To assess whether the length of the intervention duration and the difference in intervention measures affect that CMC lower levels of RF and anti-CCP, we conducted a subgroup analysis of the primary outcomes RF and anti-CCP. The subgroup analysis was based on the different number of weeks in terms of intervention duration (4 weeks; 6 weeks; 8 weeks; 9 weeks; 12 weeks; 14 weeks; 16 weeks; 24 weeks; 36 weeks; not reported), and different intervention measures (CMC monotherapy or CMC combined with Western medicine) specified the subgroups. Table 3 summarizes the results of RF and anti-CCP subgroup analysis.

**TABLE 3:** The results of meta-analysis of RF and anti-CCP subgroup analysis.

Variable	No. of Trials	No. of EG	No. of CG	SMD (95% CI)	*I* ^2^ (%)
RF					
Intervention Duration					
4W	4	253	238	−2.93 (−4.56, −1.31)	98
6W	2	67	63	−0.63 (−1.05, −0.21)	27
8W	8	339	312	−0.86 (−1.07, −0.66)	34
9W	1	55	55	−0.65 (−1.03, −0.27)	NA
12W	36	1,600	1,522	−0.73 (−0.95, −0.52)	88
14W	1	31	30	−3.53 (−4.35, −2.71)	NA
16W	2	117	117	−1.60 (−4.02, 0.83)	98
24W	7	304	304	−0.90 (−1.57, −0.23)	93
36W	1	40	20	−2.99 (−3.77, −2.22)	NA
NR	1	34	36	−0.77 (−1.26, −0.29)	NA
Intervention Measure					
CMC	12	530	498	−0.72 (−1.27, −0.17)	94
CMC + WM	51	2,513	2,398	−0.91 (−1.10, −0.73)	91
Anti-CCP					
Intervention Duration					
12W	6	389	378	−0.67 (−0.89, −0.45)	54
24W	2	76	76	−0.18 (−0.62, 0.26)	47
Intervention Measure					
CMC	1	45	45	0.02 (−0.39, 0.44)	NA
CMC + WM	7	420	409	−0.65 (−0.85, −0.45)	49

EG, experimental group; CG, control group; W, weeks; CMC, Chinese medicine compound; WM, Western medicine; SMD, standardized mean difference; NR, not reported; NA, not applicable.

We found that the heterogeneity could not be eliminated after grouping according to the results of subgroup analysis, indicating that differences in intervention duration and intervention measures might not be the underlying source of heterogeneity. Combined with the results of subgroup analysis and sensitivity analysis, we discussed the heterogeneity between studies, considering that the heterogeneity between included studies was mainly related to the following aspects:

1) There were differences in the CMC components as well as the dosage regimens of intervention measures among studies; 2) The average age and disease course of the included participants were different, resulting in varying severity of the disease; 3) Due to the slow-acting of the herbal medicine, it usually takes several months after treatment to show significant therapeutic effects. However, the duration of intervention varies greatly from study to study, which may lead to the heterogeneity of the results.

### Publication Bias

Analysis for publication bias was performed using Egger’s test for endpoints of RF. Egger’s asymmetry coefficient, known to be low powered, did detect potential bias in the meta-analysis of RF (*p <* 0.01). These publication bias may be due to the included studies were all published in Chinese. Moreover, all literature included had positive results, and the articles with negative results were often not able to be published, which caused publication bias to some extent. As described above, the publication bias could not be excluded.

### Frequency Distribution Analysis

A total of 71 CMCs were recorded, and 156 single Chinese medicines were obtained, which were sorted by frequency of occurrence from high to low, we listed the Chinese medicine with a frequency of more than 10 times. There were a total of 26 traditional Chinese herbs (Table 4). The top five were Angelica sinensis (Oliv.) Diels [Apiaceae;Radix Angelicae Sinensis] (Danggui), Glycyrrhiza glabra L.[Fabaceae;Radix Glycyrrhizae] (Gancao), Neolitsea cassia (L.) Kosterm.[Lauraceae;Ramulus Cinnamomi] (Guizhi), Paeonia lactiflora Pall.[Paeoniaceae;Radix Paeoniae Alba] (Baishao), and Angelica dahurica (Hoffm.) Benth. & Hook.f. ex Franch. & Sav. [Apiaceae;Radix Angelicae Pubescentis] (Duhuo).

**TABLE 4:** The frequency of Chinese medicine (more than 10 times of traditional Chinese medicine).

No.	Pinyin name	Full format	Frequency
1	Danggui	Angelica sinensis (Oliv.) Diels [Apiaceae; Radix Angelicae Sinensis]	38
2	Gancao	Glycyrrhiza glabra L.[Fabaceae; Radix Glycyrrhizae]	32
3	Guizhi	Neolitsea cassia (L.) Kosterm.[Lauraceae; Ramulus Cinnamomi]	27
4	Baishao	Paeonia lactiflora Pall.[Paeoniaceae; Radix Paeoniae Alba]	27
5	Duhuo	Angelica dahurica (Hoffm.) Benth. & Hook.f. ex Franch. & Sav. [Apiaceae; Radix Angelicae Pubescentis]	22
6	Chuanxiong	Conioselinum anthriscoides 'Chuanxiong'[Apiaceae; Rhizoma Ligustici]	21
7	Yiyiren	Coix lacryma-jobi L.[Poaceae; Semen Coicis]	18
8	Huangqi	Astragalus mongholicus Bunge[Fabaceae; Radix Astragali seu Hedysari]	17
9	Fangfeng	Saposhnikovia divaricata (Turcz. ex Ledeb.) Schischk. [Apiaceae; Radix Saposhnikoviae]	17
10	Weilingxian	Clematis chinensis Osbeck[Ranunculaceae; Radix Clematidis]	16
11	Shudihuang	Rehmannia glutinosa (Gaertn.) DC. [Orobanchaceae; Radix Rehmanniae Preparata]	16
12	Dilong	Pheretima asiatica Michaelsen[Megascolecidae; Lumbricus]	15
13	Jixueteng	Spatholobus suberectus Dunn[Fabaceae; Caulis Spatholobi]	15
14	Qinjiao	Gentiana macrophylla Pall.[Gentianaceae; Radix Gentianae Macrophyllae]	13
15	Niuxi	Achyranthes bidentata Blume [Amaranthaceae; Radix Achyranthis Bidentatae]	13
16	Qianghuo	Hansenia weberbaueriana (Fedde ex H.Wolff) Pimenov & Kljuykov [Apiaceae; Rhizoma et Radix Notopterygii]	12
17	Chishao	Paeonia lactiflora Pall. [Paeoniaceae; Radix Paeoniae Rubra]	12
18	Cangzhu	Atractylodes lancea (Thunb.) DC.[Asteraceae; Rhizoma Atractylodis]	11
19	Rendongteng	Lonicera japonica Thunb. [Caprifoliaceae;Caulis Lonicerae]	11
20	Baizhu	Atractylodes macrocephala Koidz.[Asteraceae; Rhizoma Atractylodis Macrocephalae]	11
21	Sangjisheng	Taxillus chinensis (DC.) Danser[Loranthaceae; Herba Taxilli]	11
22	Wushaoshe	Zaocys dhumnades(Cantor) [Natricinae; Zaocys dhumnades]	11
23	Quanxie	Buthus martensii Karsch[Scorpiones; Scorpio]	11
24	Baijiezi	Sinapis alba L. [Brassicaceae; Semen sinapis.]	10
25	Mahuang	Ephedra sinica Stapf [Ephedraceae; Herba Ephedrae]	10
26	Huangbo	Phellodendron amurense Rupr. [Rutaceae; Cortex Phellodendri]	10

## Discussion

### Main Findings

RF and anti-CCP belong to the serum biomarkers involved in the 2010 ACR/EULAR RA classification criteria, and they precede the onset of clinical symptoms and predict a more severe disease course, indicating a pathogenic role in RA (Kay and Upchurch, 2012).

There is a study confirmed that the combined presence of anti-CCP and IgM-RF mediates increased production of proinflammatory cytokine *in vitro* and this combination is associated with increased systemic inflammation and disease activity in RA (Moura et al., 2012). Accumulating evidences suggest that the presence of high titers of RF in combination with anti-CCP associated with increased disease activity, more aggressive arthritis, worse prognosis and reduced rates of remission in patients with RA (Jónsson et al., 1995; Moeez et al., 2013; Sokolove et al., 2014; Valesini et al., 2015). Moreover, anti-CCP not only has a great diagnostic value for RA in terms of sensitivity and specificity but also seem to be better predictors of poor prognostic features such as progressive joint destruction (Harre et al., 2012). In addition, anti-CCP status predicts response to therapy as well (Burska et al., 2014).

The observations suggest that RF and anti-CCP are capable of promoting a more accurate prognosis and by targeting these two markers will contribute to a better disease management. There is no doubt that underscores the utility of these autoantibodies in RA.

In China, CMC has a long history of treating RA, and several clinical trials have been conducted in recent years on the treatment of RA with CMC. However, to our knowledge, this is the first systematic review and meta-analysis of published RCTs to assess the effect of CMC in reducing the levels of serum RF and anti-CCP in patients with RA. We revealed that 65 RCTs observed the comparison between CMC or CMC combined with Western medicine and Western medicine monotherapy in the treatment of RA in terms of changing the serological markers RF and anti-CCP level. The pooled analysis showed that after treatment, serum RF and anti-CCP levels were decreased more significantly in the experimental group compared to the Western medicine control groups than at baseline. It indicated that CMC in the treatment of RA had a potential role in reducing serum RF and anti-CCP levels. In addition, we found that the CMC group had better ACR response and more effective clinical efficacy of TCM syndromes. The RR of ACR70 was higher than that of ACR20 and ACR50, and the proportion of patients achieving an ACR20 response was not significant than that of ACR50 and ACR70 response. This might be partially explained as high heterogeneity as well as difference in effect estimates observed in ACR20. The study of Chen (Chen et al., 2010) might be the source of the heterogeneity, which was related to the contradiction between the results reported in his study with other studies. In the results of this study, the ACR20 response rate was lower in the experimental group than in the control group, and the difference was not statistically significant. In addition, in the meta-analysis of ACR50, we found that the two studies of Zhao (Zhao, 2015) and He (He and Xiao, 2014) showed a high confidence interval value, indicating lower external validity.

Furthermore, this study summarized the frequency of CMC constituent involved in 65 research literatures and found that the top five from high to low were Angelica sinensis (Oliv.) Diels [Apiaceae;Radix Angelicae Sinensis] (Danggui), Glycyrrhiza glabra L.[Fabaceae;Radix Glycyrrhizae] (Gancao), Neolitsea cassia (L.) Kosterm.[Lauraceae;Ramulus Cinnamomi] (Guizhi), Paeonia lactiflora Pall.[Paeoniaceae;Radix Paeoniae Alba] (Baishao), and Angelica dahurica (Hoffm.) Benth. & Hook.f. ex Franch. & Sav. [Apiaceae;Radix Angelicae Pubescentis] (Duhuo). The active ingredients and pharmacological effects of the above five high-frequency representative Chinese herb were summarized in Table 5, and as illustrated in this table, most of the main active ingredients of the five Chinese herbs have immunomodulatory effects. The polysaccharides isolated from Angelica sinensis (Oliv.) Diels [Apiaceae;Radix Angelicae Sinensis] (Danggui) can regulate the immune response by regulating expression of Th1 and Th2 related cytokines (Chen et al., 2013). Cinnamaldehyde is isolated from the essential oil produced from the Neolitsea cassia (L.) Kosterm.[Lauraceae;Ramulus Cinnamomi] (Guizhi), and it has been reported to reduce the release of pro-inflammatory cytokines by inhibiting the activation of macrophages and monocytes through suppression of the mitogen-activated protein kinases (MAPKs) signaling pathway (Chao et al., 2008). Total glycoside of paeony (TGP) is extracted from the dried root of Paeonia lactiflora Pall.[Paeoniaceae;Radix Paeoniae Alba] (Baishao). Paeoniflorin (Pae) is the major active component of TGP. Pae can balance the subsets of immune cells through inhibiting abnormal activated cell subsets and restoring regulatory cell subsets by integrating multiple signaling pathways, such as JAK2/STAT3 pathway, MAPKs/NF-κB pathway, PI3K/Akt/mTOR pathway, etc. (Zhang et al., 2019; Zhang and Wei, 2020). It is well known that the pathogenesis of RA is related to B lymphocytes, and RF and anti-CCP are considered to be the results of B lymphocyte activation and differentiation into plasma cells (Cambridge et al., 2003; Dörner et al., 2004). One study confirmed through animal experiments that Pae inhibits the activation and proliferation of B cells by selectively blocking the LPS/TLR4 signaling pathway (Zhang et al., 2015). In addition, another study found that Pae can regulate PI3K/Akt/mTOR pathways mediated by BAFF (B-cell activating factor)/BAFF-R, thereby inhibiting antibody production by B lymphocytes in CIA rats. This may be one of the mechanisms of action of Pae to downregulate antibodies production and treat RA (Li et al., 2012b). Therefore, we figured that the mechanism by which TCM or CMC can reduce serum RF and anti-CCP levels might be related to the regulation of the immune response and inhibition of B lymphocytes proliferation.

**TABLE 5:** Main active ingredients, pharmacological effects and mechanisms of the top five Chinese medicine in frequency distribution.

Pinyin name	Full Format	Main active ingredients	Pharmacological effects and mechanisms
Danggui	Angelica sinensis (Oliv.) Diels [Apiaceae; Radix Angelicae Sinensis]	Polysaccharides	Immunomodulatory activity: regulating the expression of Th1- and Th2-related cytokines (Chen et al., 2013)
Gancao	Glycyrrhiza glabra L.[Fabaceae; Radix Glycyrrhizae]	Glycyrrhizin acid, Glycyrrhetinic acid, Licorice flavonoids, Licorice polysaccharides	Anti-inflammatory: Inhibiting the transcriptions of iNOS, COX-2, TNF-α and IL-6 by blocking the activation of NF-κB signaling pathways (Kim et al., 2008; Chang et al., 2010)
Guizhi	Neolitsea cassia (L.) Kosterm.[Lauraceae; Ramulus Cinnamomi]	Cinnamaldehyde	Immuno-modulating: Inhibiting the production of proinflammatory cytokines (IL-1β, TNF-α, etc.) production within LPS, LTA and polyIC stimulated macrophages and monocytes (Chao et al., 2008)
Baishao	Paeonia lactiflora Pall.[Paeoniaceae; Radix Paeoniae Alba]	Total glucosides of paeony	Anti-inflammatory and immune-regulatory: Inhibiting the activation of macrophages NF-κB and the release of inflammatory cytokines such as TNF-α, IL-1β, IL-17, and inhibit the proliferation of B lymphocytes (Zhang et al., 2019; Zhang and Wei, 2020)
Duhuo	Angelica dahurica (Hoffm.) Benth. & Hook.f. ex Franch. & Sav. [Apiaceae; Radix Angelicae Pubescentis]	Angelicae Pubescentis Radix's volatile oil	Anti-inflammatory and analgesic: Inhibiting COX-1 and COX-2 to varying degrees (Fan et al., 2009; Qiu et al., 2011)

Th, T helper; iNOS, inducible nitric oxide synthase; COX-, cyclooxygenase-; TNF-α, tumor necrosis factor-α; IL-, interleukin-; NF-κB, nuclear factor-kappa B; LPS, lipopolysaccharide; LTA, lipoteichoic acid; polyIC, polyinosinic-polycytidylic acid.

### Limitations for Research

There are some limitations in our systematic review. Firstly, since there has been no standardized CMC abroad, the above researches were all completed in China. Moreover, the foundation and clinical effects of CMC in other countries may be inconsistent with these data. With the successful development and proven definitive efficacy and safety of biologicals worldwide, biologicals are now more commonly used in the treatment of RA in many places. However, their use is not common in China as Europe, which is due to the more expensive treatment price and economic cost of biologicals compared to traditional treatment options, and this is also one of the reasons why early, adequate, and full course use of biologicals is difficult for RA patients in many developing countries (Joyce et al., 2014). It follows that among the studies included in the systematic review and meta-analysis, we found that only one (Wei and Liu, 2013) of the interventions used biologicals. Therefore, the conclusions of this systematic review have some limitations. Secondly, the overall quality of the literature included was poor, and the design of the methodological part need to be improved. Most of the included studies had inadequate or missing descriptions of randomization, allocation concealment and blinding method, resulting in an inability to fully assess the internal validity of the trials. Additionally, the sample size of most studies was too small to perform robust analysis, which fundamentally affected the strength of synthesis evidence, and therefore resulting in the limitations and conservatism of this systematic review. Thirdly, due to the different dosage forms used in each study intervention (including decoction, capsule, tablet, pill, etc.), the composition and dosage of CMC administered were also different. These had led to a diversity of intervention measures with no uniform standard, which may lead to heterogeneity. If all CMC intervention measures are combined into the same category of treatment for analysis, the inferred results of meta-analysis would be limited to some extent. Finally, outcome measures and measuring instruments for RF and anti-CCP varied from studies.

## Conclusion

In summary, CMC may be an effective treatment to treat RA. In terms of reducing the levels of RF and anti-CCP, CMC (or CMC combined with Western medicine) is more effective than only Western medicine.

Through a systematic review of the potency of CMC to change serum biomarkers levels and the clinical efficacy for the treatment of RA, we found that CMC may be a potential and efficacious therapeutic adjunct to delay the progression and improve outcomes of RA. In the future, RCTs with larger samples and higher quality should be carried out to provide more accurate and complete data to support and verify the potential of TCM compound in the treatment of RA.

## Data Availability

The original contributions presented in the study are included in the article/Supplementary Material, further inquiries can be directed to the corresponding author.
