# Supplementary figures and images for: The Effect of Chinese Medicine Compound in the Treatment of Rheumatoid Arthritis on the Level of Rheumatoid Factor and Anti-Cyclic Citrullinated Peptide Antibodies: A Systematic Review and Meta-Analysis
Source: Front Pharmacol. 2021 Jun 30;12:686360. doi: 10.3389/fphar.2021.686360 (PMC8278104; doi:10.3389/fphar.2021.686360)

Supplementary Material 2

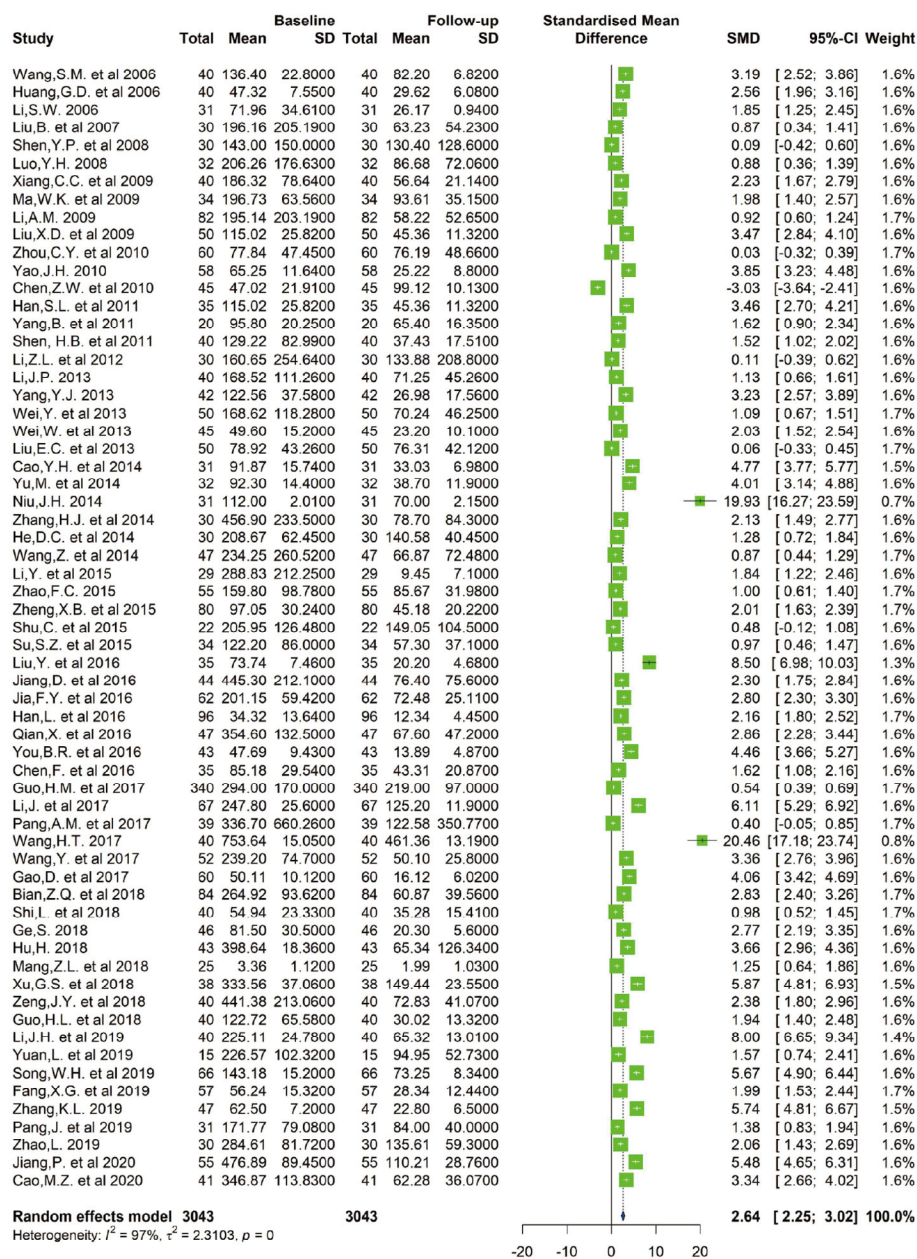

Serum level of RF level in treatment group(Baseline VS Follow-up)

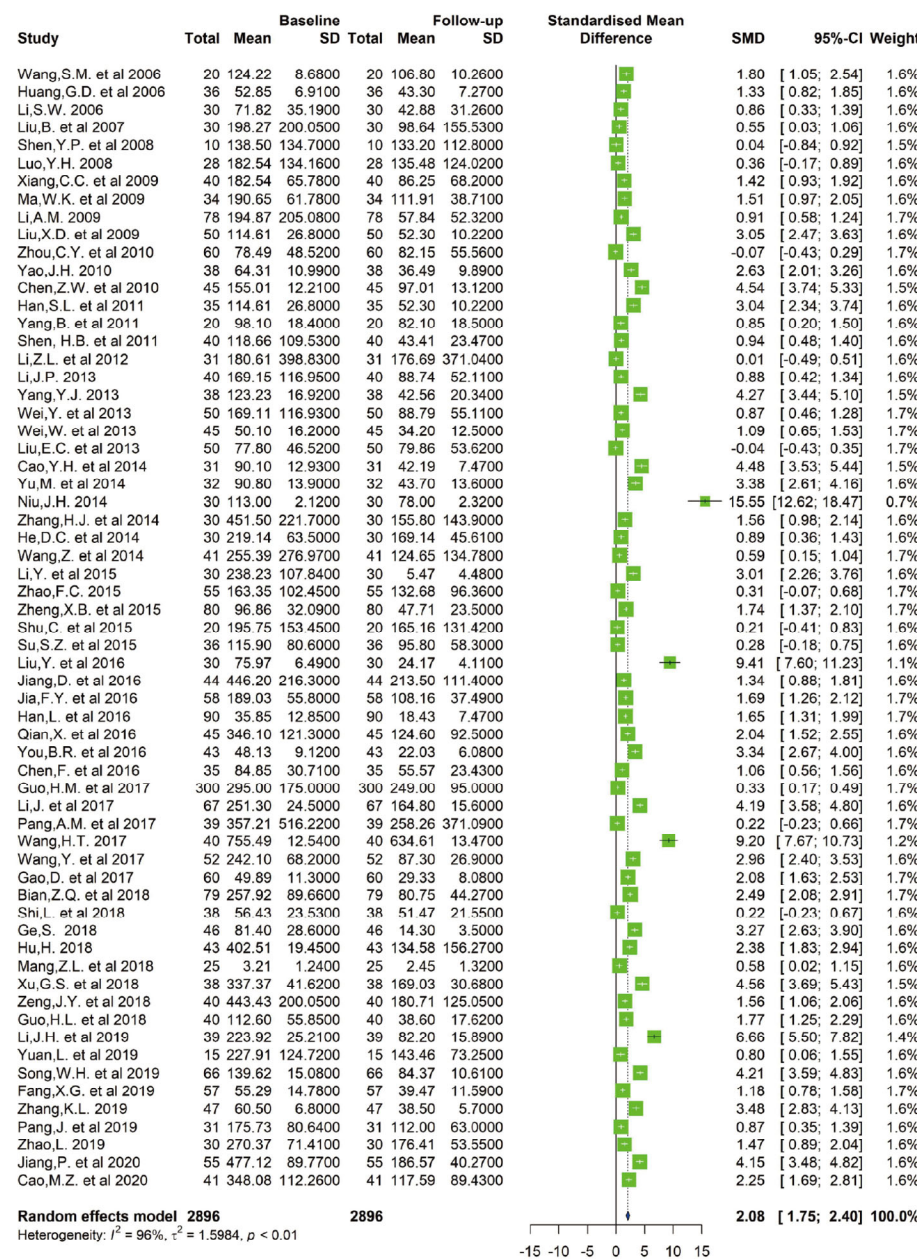

Supplement: Supplementary file 1 [file DataSheet2.pdf]
