# Supplementary material for: The Effect of Chinese Medicine Compound in the Treatment of Rheumatoid Arthritis on the Level of Rheumatoid Factor and Anti-Cyclic Citrullinated Peptide Antibodies: A Systematic Review and Meta-Analysis
Source: Front Pharmacol. 2021 Jun 30;12:686360. doi: 10.3389/fphar.2021.686360 (PMC8278104; doi:10.3389/fphar.2021.686360)
Supplement: Supplementary file 3 [file DataSheet3.pdf]

Supplementary Material 3

Study	Baseline Total	Baseline Mean	Baseline SD	Follow-up Total	Follow-up Mean	Follow-up SD	SMD	95%-CI	Weight
Liu,X.D. et al 2009	50	101.26	21.4300	50	42.62	7.3100	3.63	[2.99; 4.28]	12.2%
Chen,Z.W. et al 2010	45	0.25	0.4500	45	0.22	0.4500	0.07	[-0.35; 0.48]	12.7%
Han,L. et al 2016	96	38.23	14.2800	96	17.45	6.4600	1.87	[1.53; 2.21]	12.8%
Pang,A.M. et al 2017	39	650.26	522.2600	39	331.08	109.2600	0.84	[0.37; 1.30]	12.6%
Gao,D. et al 2017	60	398.41	20.5500	60	273.01	32.0100	4.63	[3.94; 5.33]	12.1%
Bian,Z.Q. et al 2018	84	96.58	37.2500	84	24.88	17.0600	2.46	[2.06; 2.87]	12.7%
Pang,J. et al 2019	31	181.67	66.2100	31	78.00	29.0000	2.00	[1.39; 2.62]	12.3%
Li,D. 2020	60	493.37	210.2100	60	196.72	83.3700	1.84	[1.41; 2.27]	12.7%
**Random effects model**	**465**			**465**			**2.15**	**[1.28; 3.01]**	**100.0%**

Heterogeneity: $I^2 = 96\%$, $\tau^2 = 1.4805$, $p < 0.01$

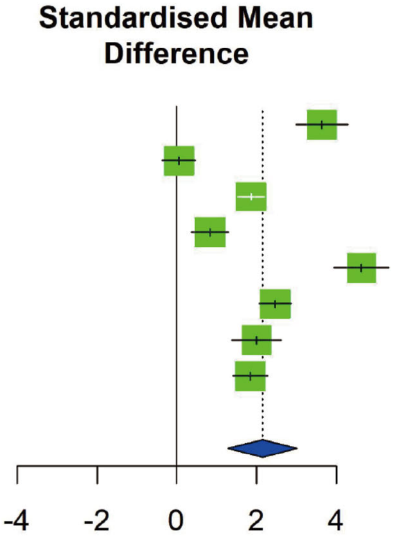

Serum level of anti-CCP level in treatment group(Baseline VS Follow-up)

Study	Baseline Total	Baseline Mean	Baseline SD	Follow-up Total	Follow-up Mean	Follow-up SD	SMD	95%-CI	Weight
Liu,X.D. et al 2009	50	98.85	20.5400	50	45.77	7.1200	3.43	[2.80; 4.05]	12.0%
Chen,Z.W. et al 2010	45	0.23	0.5600	45	0.21	0.3500	0.04	[-0.37; 0.46]	12.7%
Han,L. et al 2016	90	38.12	13.0700	90	25.67	8.7400	1.12	[0.80; 1.43]	12.9%
Pang,A.M. et al 2017	39	611.67	516.9000	39	416.89	213.7200	0.49	[0.04; 0.94]	12.6%
Gao,D. et al 2017	60	399.82	26.8800	60	300.89	37.2200	3.03	[2.50; 3.56]	12.3%
Bian,Z.Q. et al 2018	79	95.86	36.5700	79	38.04	20.3900	1.94	[1.56; 2.32]	12.7%
Pang,J. et al 2019	31	183.37	71.6700	31	93.00	39.0000	1.55	[0.97; 2.12]	12.2%
Li,D. 2020	60	473.58	204.6400	60	233.43	96.3400	1.49	[1.09; 1.90]	12.7%
**Random effects model**	**454**			**454**			**1.62**	**[0.91; 2.33]**	**100.0%**

Heterogeneity: $I^2 = 95\%$, $\tau^2 = 0.9829$, $p < 0.01$

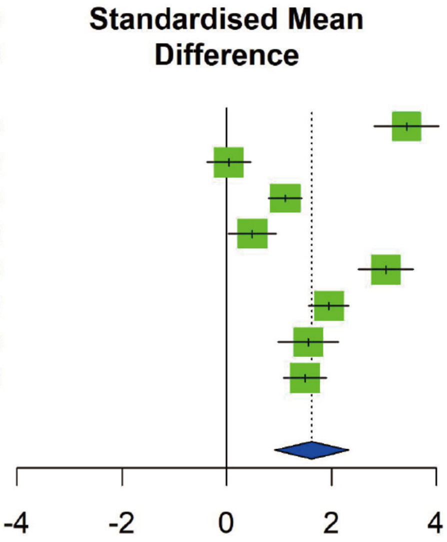

Serum level of anti-CCP level in control group(Baseline VS Follow-up)
