# Supplementary material for: The Effect of Chinese Medicine Compound in the Treatment of Rheumatoid Arthritis on the Level of Rheumatoid Factor and Anti-Cyclic Citrullinated Peptide Antibodies: A Systematic Review and Meta-Analysis
Source: Front Pharmacol. 2021 Jun 30;12:686360. doi: 10.3389/fphar.2021.686360 (PMC8278104; doi:10.3389/fphar.2021.686360)
Supplement: Supplementary file 4 [file DataSheet1.docx]

| **Literature** | **Year** | **Intervention methods** | |  | **Comment** |
| --- | --- | --- | --- | --- | --- |
|  |  | **EG** | **CG** | **Species, concentration** |  |
| Clinical observation on treatment of Rheumatoid arthritis by Sanbi Decoction[1] | 2006 | MTX(10mg qw)+SSZ(0.75g tid)+Sanbi Decoction(bid) | MTX(10mg qw)+SSZ(0.75g tid)+Meloxicam*(7.5mg qd) | Gentiana macrophylla Pall.[Gentianaceae;Radix Gentianae Macrophyllae], 9g; Angelica dahurica (Hoffm.) Benth. & Hook.f. ex Franch. & Sav. [Apiaceae;Radix Angelicae Pubescentis], 9g; Saposhnikovia divaricata (Turcz. ex Ledeb.) Schischk. [Apiaceae;Radix Saposhnikoviae], 6g; Asarum sieboldii[Aristolochiaceae；Herba cum Radix Asari], 3g; Conioselinum anthriscoides ‘Chuanxiong’[Apiaceae;Rhizoma Ligustici], 9g; Angelica sinensis (Oliv.) Diels[Apiaceae;Radix Angelicae Sinensis], 12g; Rehmannia glutinosa (Gaertn.) DC. [Orobanchaceae;Radix Rehmanniae Preparata], 15g; Paeonia lactiflora Pall.[Paeoniaceae;Radix Paeoniae Alba], 12g; Neolitsea cassia (L.) Kosterm.[Lauraceae;Ramulus Cinnamomi], 6g; Poria[Polyporaceae; Sclerotium Poriae Cocos], 12g; Eucommia ulmoides Oliv. [Eucommiaceae; Cortex Eucommiae], 15g; Achyranthes bidentata Blume [Amaranthaceae;Radix Achyranthis Bidentatae], 30g; Codonopsis pilosula[Campanulaceae; Radix Codonopsis], 12g; Glycyrrhiza glabra[Fabaceae; Radix Glycyrrhizae], 10g; Astragalus mongholicus Bunge[Fabaceae;Radix Astragali seu Hedysari], 20g; Dipsacus asper[Caprifoliaceae; Radix Dipsaci], 15g; Citrus × aurantium L.[Rutaceae;Pericarpium Citri Reticulatae], 9g; Zingiber officinale[Zingiberaceae; Rhizoma Zingiberis], 9g |  |
| Observation on effect of Centipede Longsnake Decoction combined with Western medicine in treating rheumatoid arthritis[2] | 2006 | MTX(10mg qw)+D-PEN(250mg bid)+Longsnake Decoction*(bid) | MTX(10mg qw)+D-PEN(250mg bid) | Centipede[Scolopendridae; Scolopendra], 1g; Pheretima asiatica Michaelsen[Megascolecidae;Lumbricus], 10g; Agkistrodon[Deinagkistrodon;Bungarus], 1g; Aconitum carmichaeli Debeaux [Ranunculaceae;Radix Aconiti], 3g(decocted earlier); Lindera aggregata (Sims) Kosterm. [Lauraceae; Radix Linderae], 3g(decocted earlier); Calamus draco Willd. [Calamus draco Willd.; Sanguis Draconis], 3g; Arisaema heterophyllum Blume [Araceae; Rhizoma Arisaematis], 5g(decocted earlier); Eupolyphaga[Corydidae;Eupolyphaga Seu Steleophaga], 5g; Smilax nipponica [Magnoliaceae; Dashenjin], 10g; Clematis chinensis Osbeck[Ranunculaceae;Radix Clematidis], 10g; Frankincense[Burseraceae; Olibanum], 10g; Commiphora myrrha[Burseraceae; Resina Commiphorae], 10g; Dipsacus asper[Caprifoliaceae; Radix Dipsaci], 10g; Angelica dahurica (Hoffm.) Benth. & Hook.f. ex Franch. & Sav.[Apiaceae;Radix Angelicae Dahuricae], 10g; Garden Balsam Stem [Euphorbiaceae; Speranskia tuberculata (Bunge) Baill], 10g; Angelica sinensis (Oliv.) Diels[Apiaceae;Radix Angelicae Sinensis], 20g; Conioselinum anthriscoides ‘Chuanxiong’[Apiaceae;Rhizoma Ligustici], 20g; Glycyrrhiza glabra[Fabaceae; Radix Glycyrrhizae], 20g |  |
| Observation of 40 cases of rheumatoid arthritis treated by combination of Traditional Chinese and Western medicine[3] | 2006 | MTX(7.5-15mg qw)+SSZ(0.75g tid)+NASIDs*+Siwu Wuteng Decoction&(bid) | MTX(7.5-15mg qw)+SSZ(0.75g tid)+NASIDs* | Rehmannia glutinosa (Gaertn.) DC. [Orobanchaceae;Radix Rehmanniae Preparata], 12g; Paeonia lactiflora Pall. [Paeoniaceae;Radix Paeoniae Rubra], 10g; Paeonia lactiflora Pall.[Paeoniaceae;Radix Paeoniae Alba], 10g; Angelica sinensis (Oliv.) Diels[Apiaceae;Radix Angelicae Sinensis], 15g; Conioselinum anthriscoides ‘Chuanxiong’[Apiaceae;Rhizoma Ligustici], 10g; Sinomenium acutum[Menispermaceae; Caulis Sinomenii], 15-30g; Tripterygium wilfordii[Celastraceae; Radix Tripterygii Wilfordii], 10g; Trachelospermum jasminoides (Lindl.) Lem. [Apocynaceae; Caulis Trachelospermi], 15-30g; Spatholobus suberectus Dunn[Fabaceae;Caulis Spatholobi], 30g; Reynoutria multiflora (Thunb.) Moldenke [Polygonaceae; Caulis Polygoni Multiflori], 30g | *: Discontinue when symptoms improve |
| Siwu Decoction combined with methotrexate attenuated and effective treatment for rheumatoid arthritis[4] | 2007 | MTX(15mg qw)+Siwu Decoction(tid) | MTX(15mg qw) | Angelica sinensis (Oliv.) Diels[Apiaceae;Radix Angelicae Sinensis], 10g; Conioselinum anthriscoides ‘Chuanxiong’[Apiaceae;Rhizoma Ligustici], 8g; Paeonia lactiflora Pall.[Paeoniaceae;Radix Paeoniae Alba], 10g; Rehmannia glutinosa (Gaertn.) DC. [Orobanchaceae;Radix Rehmanniae Preparata], 15g |  |
| thirty-two  cases of senile rheumatoid arthritis were treated with Jianpi Yiqi Huoxue Decoction[5] | 2008 | Jianpi Yiqi Huoxue Decoction(bid) | MTX(7.5mg-15mg qw)+SSZ(0.75g-1.0g bid)+Meloxicam(7.5mg qd-bid) | Astragalus mongholicus Bunge[Fabaceae;Radix Astragali seu Hedysari], 30g; Codonopsis pilosula[Campanulaceae; Radix Codonopsis], 15g; Atractylodes macrocephala Koidz.[Asteraceae;Rhizoma Atractylodis Macrocephalae], 15g; Paeonia × suffruticosa [Paeoniaceae;Cortex Moutan Radicis], 15g; Gentiana macrophylla Pall.[Gentianaceae;Radix Gentianae Macrophyllae], 15g; Zaocys dhumnades(Cantor)[Natricinae;Zaocys dhumnades], 15g; Dioscorea oppositifolia[Dioscoreaceae; Rhizoma Dioscoreae], 18g; Coix lacryma-jobi L.[Poaceae;Semen Coicis], 18g; Curcuma longa[Zingiberaceae; Rhizoma Curcumae Longae], 12g; Bombyx mori Linnaeus[Bombyx Linnaeus;Bombyx Batryticatus], 9g; Carthamus tinctorius[Asteraceae; Flos Carthami], 6g; Buthus martensii Karsch[Scorpiones;Scorpio], 3g; Asarum sieboldii[Aristolochiaceae; Herba cum Radix Asari], 3g |  |
| Effect of Ziyin YiQi Decoction on hormone withdrawal in rheumatoid arthritis[6] | 2008 | Piroxicam Tablets(20mg qd)+Prednisone*(Original dose)+Ziyin YiQi Decoction | Piroxicam Tablets(20mg qd)+Prednisone*(Original dose) | Rehmannia glutinosa[Orobanchaceae; Radix Rehmanniae]; Ophiopogon japonicus (Thunb.) Ker Gawl. [Asparagaceae; Radix Ophiopogonis]; Rehmannia glutinosa (Gaertn.) DC. [Orobanchaceae;Radix Rehmanniae Preparata] ;Eucommia ulmoides Oliv. [Eucommiaceae; Cortex Eucommiae] ; Achyranthes bidentata Blume [Amaranthaceae;Radix Achyranthis Bidentatae] ; Taxillus chinensis (DC.) Danser[Loranthaceae; Herba Taxilli] ; Astragalus mongholicus Bunge[Fabaceae;Radix Astragali seu Hedysari] ; Pseudostellaria heterophylla (Miq.) Pax [Caryophyllaceae; Radix Pseudostellariae heterophylly] ; Angelica sinensis (Oliv.) Diels[Apiaceae;Radix Angelicae Sinensis] ;Salvia miltiorrhiza Bunge[Lamiaceae; Radix Salviae Miltiorrhizae]; Polygonatum sibiricum Redouté [Asparagaceae; Rhizoma Polygonati] ;Gentiana macrophylla Pall.[Gentianaceae;Radix Gentianae Macrophyllae] ; Angelica dahurica (Hoffm.) Benth. & Hook.f. ex Franch. & Sav. [Apiaceae;Radix Angelicae Pubescentis] ; Spatholobus suberectus Dunn[Fabaceae;Caulis Spatholobi] ; Silkworm shit [Bombycidae; Bombyx mori L.] ; Glycyrrhiza glabra[Fabaceae; Radix Glycyrrhizae], | *：Decrease 2.50-5.00 mg per week depending on the condition, and 1.25-2.50 mg per week for prednisone doses less than 10 mg/d. |
| Forty cases of rheumatoid arthritis were treated with Bushen Decoction and Western medicine[7] | 2009 | MTX(10-15mg qw)+SSZ(0.75g-1.0g bid)+Celebrex(200mg qd-bid)+Bushen Decoction(bid) | MTX(10-15mg qw)+SSZ(0.75g-1.0g bid)+Celebrex(200mg qd-bid) | Gentiana macrophylla Pall.[Gentianaceae;Radix Gentianae Macrophyllae], 15g; Eucommia ulmoides Oliv. [Eucommiaceae; Cortex Eucommiae], 15g; Dipsacus asper[Caprifoliaceae; Radix Dipsaci], 15g; Rehmannia glutinosa (Gaertn.) DC. [Orobanchaceae;Radix Rehmanniae Preparata], 15g; Angelica dahurica (Hoffm.) Benth. & Hook.f. ex Franch. & Sav. [Apiaceae;Radix Angelicae Pubescentis], 15g; Taxillus chinensis (DC.) Danser[Loranthaceae; Herba Taxilli], 15g; Reynoutria multiflora (Thunb.) Moldenke[Polygonaceae;Radix Polygoni Multiflori], 15g; Lindera aggregata (Sims) Kosterm. [Lauraceae; Radix Linderae], 15g; Pheretima asiatica Michaelsen[Megascolecidae;Lumbricus], 10g; Bombyx mori Linnaeus[Bombyx Linnaeus;Bombyx Batryticatus], 10g; Neolitsea cassia[Lauraceae; Cortex Cinnamomi], 10g; Astragalus mongholicus Bunge[Fabaceae;Radix Astragali seu Hedysari], 30g |  |
| Clinical study on the treatment of rheumatoid arthritis by Sanwu capsule combined with methotrexate and salazosulphapyridine[8] | 2009 | MTX(10mg qw)+SSZ(1.0g bid)+Meloxicam*(7.5mg qd)+Sanwu capsule(4# tid) | MTX(10mg qw)+SSZ(1.0g bid)+Meloxicam*(7.5mg qd) | Panax notoginseng (Burkill) F.H.Chen[Araliaceae;Radix Notoginseng] ; Lindera aggregata (Sims) Kosterm. [Lauraceae; Radix Linderae] ; Sinomenium acutum[Menispermaceae; Caulis Sinomenii] ; Cibotium barometz (L.) J.Sm.[Cyatheaceae;Rhizoma Cibotii] ; Homalomena occulta (Lour.) Schott [Araceae; Rhizoma Homalomenae] ; Curcuma longa[Zingiberaceae; Rhizoma Curcumae Longae] ; Paeonia lactiflora Pall.[Paeoniaceae;Radix Paeoniae Alba]; etc. | *：A COX-2 inhibitor, taken only for the first 15 days after enrollment |
| Siteng Yin and Siwu Tang were used to treat 160 cases of rheumatoid arthritis[9] | 2009 | Siteng Yin and Siwu Tang(bid) | MTX(10mg qw)+Diclofenac Sodium Enteric-coated Tablets(25mg tid) | Tripterygium wilfordii[Celastraceae; Radix Tripterygii Wilfordii], 15g; Sinomenium acutum[Menispermaceae; Caulis Sinomenii], 20g; Lonicera japonica Thunb. [Caprifoliaceae;Caulis Lonicerae], 20g; Spatholobus suberectus Dunn[Fabaceae;Caulis Spatholobi], 30g; Morus alba[Moraceae; Ramulus Mori], 30g; Paeonia lactiflora Pall.[Paeoniaceae;Radix Paeoniae Alba], 30g; Anemarrhena asphodeloides[Liliaceae; Rhizoma Anemarrhenae], 8g; Saposhnikovia divaricata (Turcz. ex Ledeb.) Schischk. [Apiaceae;Radix Saposhnikoviae], 12g; Epimedium sagittatum (Siebold & Zucc.) Maxim. [Berberidaceae;Herba Epimedii], 15g; Conioselinum anthriscoides ‘Chuanxiong’[Apiaceae;Rhizoma Ligustici], 15g; Pheretima asiatica Michaelsen[Megascolecidae;Lumbricus], 12g; Buthus martensii Karsch[Scorpiones;Scorpio], 10g; Taxillus chinensis (DC.) Danser[Loranthaceae; Herba Taxilli], 15g; Angelica sinensis (Oliv.) Diels[Apiaceae;Radix Angelicae Sinensis], 10g; Rehmannia glutinosa (Gaertn.) DC. [Orobanchaceae;Radix Rehmanniae Preparata], 15g |  |
| Effect of Wenhua Juanbi Decoction on TNF and IL-1 in peripheral blood of patients with rheumatoid arthritis[10] | 2009 | MTX(10mg qw)+SSZ(0.5g tid)+Meloxicam*(7.5mg bid)+Wenhua Juanbi Decoction*(bid) | MTX(10mg qw)+SSZ(0.5g tid)+Meloxicam*(7.5mg bid) | Saposhnikovia divaricata (Turcz. ex Ledeb.) Schischk. [Apiaceae;Radix Saposhnikoviae] ;Angelica dahurica (Hoffm.) Benth. & Hook.f. ex Franch. & Sav.[Apiaceae;Radix Angelicae Dahuricae] ; Clematis chinensis Osbeck[Ranunculaceae;Radix Clematidis], ; Buthus martensii Karsch[Scorpiones;Scorpio] ; Centipede[Scolopendridae; Scolopendra] ; Sinapis alba L. [Brassicaceae;Semen sinapis.] ; Lonicera japonica Thunb. [Caprifoliaceae;Caulis Lonicerae] ; Salvia miltiorrhiza Bunge[Lamiaceae; Radix Salviae Miltiorrhizae] ; Coix lacryma-jobi L.[Poaceae;Semen Coicis] ; Bombyx mori Linnaeus[Bombyx Linnaeus;Bombyx Batryticatus] ; Corydalis yanhusuo[Papaveraceae;Rhizoma Corydalis] ; Atractylodes macrocephala Koidz.[Asteraceae;Rhizoma Atractylodis Macrocephalae], | *：The dosage of meloxicam tablets and MTX was gradually reduced during the treatment. |
| Efficacy of Shenshi Qianghuo Dihuang Decoction in rheumatoid arthritis: a randomized controlled trial[11] | 2010 | Shenshi Qianghuo Dihuang Decoction (100ml bid)+Meloxicam*(7.5mg qd) | MTX(15mg qw)+Meloxicam*(7.5mg qd) | Hansenia weberbaueriana (Fedde ex H.Wolff) Pimenov & Kljuykov [Apiaceae;Rhizoma et Radix Notopterygii], 30g; Rehmannia glutinosa[Orobanchaceae; Radix Rehmanniae], 30g; Astragalus mongholicus Bunge[Fabaceae;Radix Astragali seu Hedysari], 30g; Aconitum carmichaeli Debeaux [Ranunculaceae;Radix Aconiti], 9g; Sauromatum giganteum (Engl.) Cusimano [Araceae;Rhizoma Typhonii ], 9g; Caragana sinica (Buc’hoz) Rehde, 30g; Rumex japonicus Houtt. [Polygonaceae; Radix Rumicis Japonici], 30g; Sinapis alba L. [Brassicaceae;Semen sinapis.], 12g; Curcuma longa[Zingiberaceae; Rhizoma Curcumae Longae], 12g | *：A COX-2 inhibitor, taken as needed |
| Clinical observation of 58 cases of rheumatoid arthritis treated by Rebi Decoction[12] | 2010 | Rebi Decoction&(150mL bid) | LEF(20mg qd)+Nimesulide Dispersible Tablets*(0.1g bid)+Chinese medicine placebo  (150mL bid) | Tripterygium wilfordii[Celastraceae; Radix Tripterygii Wilfordii], （decocted earlier）20g; Sarcandra glabra (Thunb.) Nakai[Chloranthaceae;Herba Sarcandrae], 15g; Lonicera japonica [Caprifoliaceae;Flos Lonicerae], 24g; Equus asinus L. [Equidae; Colla Corii Asini], 24g; Scleromitrion diffusum (Willd.) R.J.Wang[Rubiaceae;Herba Hedyotis], 30g; Phellodendron amurense Rupr. [Rutaceae;Cortex Phellodendri], 12g; Atractylodes lancea (Thunb.) DC.[Asteraceae;Rhizoma Atractylodis], 9g; Coix lacryma-jobi L.[Poaceae;Semen Coicis], 30g; TuPoria[Polyporaceae; Sclerotium Poriae Cocos], 20g; Bolbostemma paniculatum (Maxim.) Franquet [Cucurbitaceae;Rhizoma Bolbostematis ], 12g; Paeonia lactiflora Pall. [Paeoniaceae;Radix Paeoniae Rubra], 20g; Hansenia weberbaueriana (Fedde ex H.Wolff) Pimenov & Kljuykov [Apiaceae;Rhizoma et Radix Notopterygii], 9g; Angelica dahurica (Hoffm.) Benth. & Hook.f. ex Franch. & Sav. [Apiaceae;Radix Angelicae Pubescentis], 9g | *：For those who cannot tolerate the swelling and pain, Nimesulide Dispersible Tablets (Nimesulide Dispersible Tablets), 0.1g/dose, twice daily, should be given orally. |
| A clinical study on Simiao Xiaobi Decoction in the treatment of rheumatoid arthritis in active stage[13] | 2010 | Simiao Xiaobi Decoction&(bid) | MTX*(10mg qw) | Lonicera japonica [Caprifoliaceae;Flos Lonicerae], 30g; Angelica sinensis (Oliv.) Diels[Apiaceae;Radix Angelicae Sinensis], 20g; Scrophularia ningpoensis[Scrophulariaceae; Radix Scrophulariae], 20g; Glycyrrhiza glabra[Fabaceae; Radix Glycyrrhizae], 10g; Scleromitrion diffusum (Willd.) R.J.Wang[Rubiaceae;Herba Hedyotis], 30g; Cremastra appendiculata (D.Don) Makino[Orchidaceae;Pseudobulbus Cremastrae seu Pleiones], 9g; Sigesbeckia orientalis[Asteraceae; Herba Siegesbeckiae], 30g; Reynoutria japonica Houtt. [Polygonaceae; Rhizoma Polygoni Cuspidati], 15g; TuPoria[Polyporaceae; Sclerotium Poriae Cocos], 20g; Paeonia lactiflora Pall.[Paeoniaceae;Radix Paeoniae Alba], 30g; Clematis chinensis Osbeck[Ranunculaceae;Radix Clematidis], 20g; Dioscorea collettii var. hypoglauca (Palib.) S.J.Pei & C.T.Ting[Dioscoreaceae;Rhizome Dioscoreae Hypoglaucae], 20g | *：Patients treated with NSAIDs and hormones (prednisone ≤10 mg/d or equivalent) entered the trial at a stable dose for at least 30 days and were maintained on subsequent therapy. Patients receiving disease-modifying antirheumatic drugs (DMARDs) must be off medication for more than 30 days. No other Chinese or Western medications with therapeutic effects in RA should be combined during the course of treatment. |
| Assessment of Clinical Effect of Therapy Combining Disease with  Syndrome on Rheumatoid Arthritis[14] | 2011 | MTX(10-15mg qw)+Loxoprofen Sodium Tablet(60mg tid)+CMC(bid) | MTX(10-15mg qw)+Loxoprofen Sodium Tablet(60mg tid) | Erodium stephanianum Willd. [Geraniaceae; Herba Geranii], 15g; Manis pentadactyla Linnaeus [Manidae; Radix Actinidiae chinensis], 15g; Sigesbeckia orientalis[Asteraceae; Herba Siegesbeckiae], 15g; TuPoria[Polyporaceae; Sclerotium Poriae Cocos], 15g; Reynoutria japonica Houtt. [Polygonaceae; Rhizoma Polygoni Cuspidati], 15g; Sanguisorba officinalis L. [Rosaceae; Radix Sanguisorbae] , 15g; Xuchangqin g 10g; Ephedra sinica Stapf [Ephedraceae;Herba Ephedrae], 5g |  |
| A randomized controlled study of Huatan Quyu Juanbi Decoction in the treatment of rheumatoid arthritis[15] | 2011 | MTX(10mg qw)+SASP(0.5g tid)+Meloxicam*(7.5mg bid)+Huatan Quyu Juanbi Decoction*(bid) | MTX(10mg qw)+SASP(0.5g tid)+Meloxicam*(7.5mg bid) | Clematis chinensis Osbeck[Ranunculaceae;Radix Clematidis], 30g; Buthus martensii Karsch[Scorpiones;Scorpio], 3 pieces; Centipede[Scolopendridae; Scolopendra], 1 piece; Sinapis alba L. [Brassicaceae;Semen sinapis.], 12g; Salvia miltiorrhiza Bunge[Lamiaceae; Radix Salviae Miltiorrhizae], 20g; Coix lacryma-jobi L.[Poaceae;Semen Coicis], 30g |  |
| Clinical observation of Simiao Pill combined with Western medicine in treating 20 cases of active rheumatoid arthritis[16] | 2011 | MTX(7.5mg qw)+Diclofenac Sodium Sustained Release Tablets(75mg qd)+Simiao Pill(150mL bid) | MTX(7.5mg qw)+Diclofenac Sodium Sustained Release Tablets(75mg qd) | Atractylodes lancea (Thunb.) DC.[Asteraceae;Rhizoma Atractylodis], 12g; Phellodendron amurense Rupr. [Rutaceae;Cortex Phellodendri], 12g; Achyranthes bidentata Blume [Amaranthaceae;Radix Achyranthis Bidentatae], 12g; Coix lacryma-jobi L.[Poaceae;Semen Coicis], 12g |  |
| Effect of tonifying kidney and removing blood stasis on bone metabolism in patients with early rheumatoid arthritis[17] | 2012 | CMC*(bid) | Alfacalcidol capsule(0.25ug qd)+Calcium D tablet(600mg qd) | Kidney-Yin deficiency syndrome: Rehmannia glutinosa (Gaertn.) DC. [Orobanchaceae;Radix Rehmanniae Preparata], 15g; Shanyu 12g; Eucommia ulmoides Oliv. [Eucommiaceae; Cortex Eucommiae], 12g; Dipsacus asper[Caprifoliaceae; Radix Dipsaci], 10g; Psoralea fructus[Fabaceae; Fructus Psoraliae], 15g; Angelica sinensis (Oliv.) Diels[Apiaceae;Radix Angelicae Sinensis], 10g; Achyranthes bidentata Blume [Amaranthaceae;Radix Achyranthis Bidentatae], 15g; Pheretima asiatica Michaelsen[Megascolecidae;Lumbricus], 10g. kidney-Yang deficiency syndrome: Aconitum carmichaeli[Ranunculaceae; Radix Aconiti Lateralis Preparata], 10g; Curculigo orchioides Gaertn.[Hypoxidaceae;Rhizoma Curculigins], 30g; Gynochthodes officinalis (F.C.How) Razafim. [Rubiaceae; Radix Morindae Officinalis], 15g; Epimedium sagittatum (Siebold & Zucc.) Maxim. [Berberidaceae;Herba Epimedii], 12g; Angelica sinensis (Oliv.) Diels[Apiaceae;Radix Angelicae Sinensis], 10g; Buthus martensii Karsch[Scorpiones;Scorpio], 2g |  |
| Clinical observation on the treatment of rheumatoid arthritis with Yaotongning Capsule[18] | 2013 | Yaotongning Capsule(4-6# qd) | MTX(10mg qw) | Strychnos nux-vomica L. [Loganiaceae; Semen Strychni]; Frankincense[Burseraceae; Olibanum] ; Commiphora myrrha[Burseraceae; Resina Commiphorae] ; Glycyrrhiza glabra[Fabaceae; Radix Glycyrrhizae]; etc. | *：Patients receiving NSAIDs and hormones (prednisone ≤ 10 mg/cl or equivalent, treatment patients into the pre-trial dose stable for at least 30 d, and maintained in the subsequent treatment, patients receiving improved course of anti-rheumatic drugs must interrupt the medication for more than 30 d, between the course of treatment shall not be combined with other Chinese and Western drugs with RA therapeutic effect . |
| Clinical observation of 50 cases of active rheumatoid arthritis treated by combination of Chinese and Western medicine[19] | 2013 | LEF(20mg qd)+SSZ(1g bid)+Meloxicam*(7.5mg bid)+Flavored Simiao Yongan Soup | LEF(20mg qd)+SSZ(1g bid)+Meloxicam*(7.5mg bid) | Lonicera japonica [Caprifoliaceae;Flos Lonicerae], 30g; Scrophularia ningpoensis[Scrophulariaceae; Radix Scrophulariae], 20g; Angelica sinensis (Oliv.) Diels[Apiaceae;Radix Angelicae Sinensis], 20g; Glycyrrhiza glabra[Fabaceae; Radix Glycyrrhizae], 10g; Chrysanthemum × morifolium (Ramat.) Hemsl.[Asteraceae;Flos Chrysanthemi], 20g; Phellodendron amurense Rupr. [Rutaceae;Cortex Phellodendri], 12g; Atractylodes lancea (Thunb.) DC.[Asteraceae;Rhizoma Atractylodis],12g; TuPoria[Polyporaceae; Sclerotium Poriae Cocos], 20g; Achyranthes bidentata Blume [Amaranthaceae;Radix Achyranthis Bidentatae], 12g; Sinomenium acutum[Menispermaceae; Caulis Sinomenii], 20g; Zaocys dhumnades(Cantor)[Natricinae;Zaocys dhumnades], 6g; Pheretima asiatica Michaelsen[Megascolecidae;Lumbricus], 10g | *：Reduce the dose to 7.5mg qd when the symptoms improve. |
| Clinical analysis of 40 cases of rheumatoid arthritis treated by combination of Chinese and Western medicine[20] | 2013 | LEF(20mg qd)+CMC(bid) | LEF(20mg qd) | Caragana sinica (Buc’hoz) Rehde, 30g; Scrophularia ningpoensis[Scrophulariaceae; Radix Scrophulariae], 25g; Coix lacryma-jobi L.[Poaceae;Semen Coicis], 25g; Hansenia weberbaueriana (Fedde ex H.Wolff) Pimenov & Kljuykov [Apiaceae;Rhizoma et Radix Notopterygii], 15g; Angelica dahurica (Hoffm.) Benth. & Hook.f. ex Franch. & Sav. [Apiaceae;Radix Angelicae Pubescentis], 15g; Sinomenium acutum[Menispermaceae; Caulis Sinomenii], 15g; Paeonia lactiflora Pall. [Paeoniaceae;Radix Paeoniae Rubra], 15g; Sigesbeckia orientalis[Asteraceae; Herba Siegesbeckiae],15g; Atractylodes lancea (Thunb.) DC.[Asteraceae;Rhizoma Atractylodis],15g; Salvia miltiorrhiza Bunge[Lamiaceae; Radix Salviae Miltiorrhizae], 10g; Clematis chinensis Osbeck[Ranunculaceae;Radix Clematidis], 10g; Bombyx mori Linnaeus[Bombyx Linnaeus;Bombyx Batryticatus], 10g; Eupolyphaga[Corydidae;Eupolyphaga Seu Steleophaga], 5g |  |
| Clinical Observation of Traditional Chinese Medicine Combined with Etanercept in Treatment of Elderly Rheumatoid Arthritis[21] | 2013 | MTX(15mg qw)+HCQ*(0.2g bid)+Diclofenac Sodium Enteric-coated Tablets(50mg bid)+Etanercept(25mg,twice a week)+CMC*(bid) | MTX(15mg qw)+HCQ*(0.2g bid)+Diclofenac Sodium Enteric-coated Tablets(50mg bid)+Etanercept(25mg,twice a week) | Centipede[Scolopendridae; Scolopendra], 2 pieces; Agkistrodon[Deinagkistrodon;Bungarus], 1 piece; Buthus martensii Karsch[Scorpiones;Scorpio], 10g; Hansenia weberbaueriana (Fedde ex H.Wolff) Pimenov & Kljuykov [Apiaceae;Rhizoma et Radix Notopterygii], 15g; Angelica dahurica (Hoffm.) Benth. & Hook.f. ex Franch. & Sav. [Apiaceae;Radix Angelicae Pubescentis], 15g; Corydalis yanhusuo[Papaveraceae;Rhizoma Corydalis], 15g; Gentiana macrophylla Pall.[Gentianaceae;Radix Gentianae Macrophyllae], 15g; Stephania tetrandra[Menispermaceae; Radix Stephaniae Tetrandrae], 15g; Poria[Polyporaceae; Sclerotium Poriae Cocos], 20g; Liquidambar formosana Hance [Altingiaceae; Fructus Liquidambaris], 20g; Astragalus mongholicus Bunge[Fabaceae;Radix Astragali seu Hedysari], 30g; Codonopsis pilosula[Campanulaceae; Radix Codonopsis], 30g; Atractylodes macrocephala Koidz.[Asteraceae;Rhizoma Atractylodis Macrocephalae], 15g; Glycyrrhiza glabra[Fabaceae; Radix Glycyrrhizae], 10g |  |
| Treatment of 42 cases of rheumatoid arthritis with Compound Maqianzi Powder[22] | 2013 | Maqianzi Powder(12.2g bid) | LEF(20mg qd)+Thymosin enteric-coated tablets(10mg qd)+Naproxen(100mg bid) | Strychnos nux-vomica L. [Loganiaceae; Semen Strychni], 0.2g; Dioscorea nipponica Makino[Dioscoreaceae;Rhizoma Dioscoreae Nipponicae], 8g; Rhododendron molle (Blume) G.Don [Ericaceae; Rhododendri mollis flos], 0.2g; Epimedium sagittatum (Siebold & Zucc.) Maxim. [Berberidaceae;Herba Epimedii], 3g; Paeonia lactiflora Pall.[Paeoniaceae;Radix Paeoniae Alba], 3g; Wushe 3g; Dibiechong 3g; Sarcandra glabra (Thunb.) Nakai[Chloranthaceae;Herba Sarcandrae], 4g |  |
| Treatment of rheumatoid arthritis by Yangxue Tongluo Recipe combined with immunosuppressive agents: a clinical observation[23] | 2014 | MTX(10mg qw)+LEF(10mg qd)+Yangxue Tongluo Decoction (bid) | MTX(10mg qw)+LEF(10mg qd) | Yangtao Actinidia Root [Actinidiaceae; Actinidia chinensis Planch.], 30g; Pheretima asiatica Michaelsen[Megascolecidae;Lumbricus], 10g; Bombyx mori Linnaeus[Bombyx Linnaeus;Bombyx Batryticatus], 10g; Poria[Polyporaceae; Sclerotium Poriae Cocos], 15g; Glycyrrhiza glabra[Fabaceae; Radix Glycyrrhizae], 5g; Spatholobus suberectus Dunn[Fabaceae;Caulis Spatholobi], 30g; Angelica sinensis (Oliv.) Diels[Apiaceae;Radix Angelicae Sinensis], 15g; Rehmannia glutinosa (Gaertn.) DC. [Orobanchaceae;Radix Rehmanniae Preparata], 15g; Coix lacryma-jobi L.[Poaceae;Semen Coicis], 30g; Astragalus mongholicus Bunge[Fabaceae;Radix Astragali seu Hedysari], 30g; Paeonia lactiflora Pall. [Paeoniaceae;Radix Paeoniae Rubra], 15g |  |
| Clinical efficacy and safety of Bizhengning in the treatment of rheumatoid arthritis[24] | 2014 | MTX(10mg qd)+LEF(10mg qd)+Bizhengnin Decoction(bid) | MTX(10mg qd)+LEF(10mg qd) | Deerhorn glue[Cervidae;Colla Corni Cervi], 10g; Aconitum carmichaeli[Ranunculaceae; Radix Aconiti Lateralis Preparata], 10g; Neolitsea cassia (L.) Kosterm.[Lauraceae;Ramulus Cinnamomi], 10g; Angelica dahurica (Hoffm.) Benth. & Hook.f. ex Franch. & Sav. [Apiaceae;Radix Angelicae Pubescentis], 10g; Asarum sieboldii[Aristolochiaceae；Herba cum Radix Asari], 5g; Angelica sinensis (Oliv.) Diels[Apiaceae;Radix Angelicae Sinensis], 15g; Clematis chinensis Osbeck[Ranunculaceae;Radix Clematidis], 15g; Sinomenium acutum[Menispermaceae; Caulis Sinomenii], 20g; Paeonia lactiflora Pall. [Paeoniaceae;Radix Paeoniae Rubra], 10g; Paeonia lactiflora Pall.[Paeoniaceae;Radix Paeoniae Alba], 10g; Pheretima asiatica Michaelsen[Megascolecidae;Lumbricus], 10g; Coix lacryma-jobi L.[Poaceae;Semen Coicis], 30g; Rehmannia glutinosa[Orobanchaceae; Radix Rehmanniae], 10g; Glycyrrhiza glabra[Fabaceae; Radix Glycyrrhizae], 15g; Centipede[Scolopendridae; Scolopendra], 2 pieces  ; Vincetoxicum mukdenense Kitag.[Apocynaceae;Radix Cynanchi Paniculati], 15g |  |
| Clinical observation of Duhuo Qushi Zhitong Decoction combined with meloxicam in treatment of rheumatoid arthritis[25] | 2014 | Meloxicam(7.5mg qd)+Duhuo Qushi Zhitong Decoction(bid) | Meloxicam(7.5mg qd) | Angelica dahurica (Hoffm.) Benth. & Hook.f. ex Franch. & Sav. [Apiaceae;Radix Angelicae Pubescentis], 15g; Clematis chinensis Osbeck[Ranunculaceae;Radix Clematidis], 15g;Buthus martensii Karsch[Scorpiones;Scorpio], 6g;Pheretima asiatica Michaelsen[Megascolecidae;Lumbricus], 12g; Atractylodes lancea (Thunb.) DC.[Asteraceae;Rhizoma Atractylodis], 12g;Neolitsea cassia (L.) Kosterm.[Lauraceae;Ramulus Cinnamomi], 10g; Phellodendron amurense Rupr. [Rutaceae;Cortex Phellodendri], 10g; Zhechong10g; Wuqiaoshe 10g; Carthamus tinctorius[Asteraceae; Flos Carthami], 10g; Achyranthes bidentata Blume [Amaranthaceae;Radix Achyranthis Bidentatae], 15g; Curcuma longa[Zingiberaceae; Rhizoma Curcumae Longae], 15g |  |
| Observation on the curative effect of Guizhi Shaoyao Zhimu Decoction combined with Juanbi Decoction in treating rheumatoid arthritis[26] | 2014 | MTX(10mg qw)+Guizhi Shaoyao Zhimu Decoction combined with Juanbi Decoction(bid) | MTX(10mg qw) | Paeonia lactiflora Pall.[Paeoniaceae;Radix Paeoniae Alba], 20g; Chaenomeles lagenaria (Loisel.) Koidz. [Rosaceae; Fructus Chaenomelis], 15g; Pyrola calliantha Andres [Ericaceae; Herba Pyrolae], 15g; Lycopodium japonicum[Lycopodiaceae; Herba Lycopodii], 15g; Atractylodes macrocephala Koidz.[Asteraceae;Rhizoma Atractylodis Macrocephalae], 15g; Neolitsea cassia (L.) Kosterm.[Lauraceae;Ramulus Cinnamomi], 12g; Gentiana macrophylla Pall.[Gentianaceae;Radix Gentianae Macrophyllae], 12g; Piper kadsura[Piperaceae; Caulis Piperis Kadsurae], 12g; Aconitum carmichaeli Debeaux [Ranunculaceae;Radix Aconiti], 10g; Saposhnikovia divaricata (Turcz. ex Ledeb.) Schischk. [Apiaceae;Radix Saposhnikoviae], 10g; Angelica sinensis (Oliv.) Diels[Apiaceae;Radix Angelicae Sinensis], 10g; Achyranthes bidentata Blume [Amaranthaceae;Radix Achyranthis Bidentatae], 10g; Frankincense[Burseraceae; Olibanum], 10g; Cremastra appendiculata (D.Don) Makino[Orchidaceae;Pseudobulbus Cremastrae seu Pleiones], 10g; Glycyrrhiza glabra[Fabaceae; Radix Glycyrrhizae], 6g; Ephedra sinica Stapf [Ephedraceae;Herba Ephedrae], 4g |  |
| Clinical observation of heat clearing and wetting combined with Western medicine in the treatment of dampness-heat type rheumatoid arthritis[27] | 2014 | LEF(10mg qd )+Meloxicam(10mg bid)+ Compound Qiyi Capsule  (3# tid)+Huangqin Qingre Chubi Capsules  (4# tid) | LEF(10mg qd) +Meloxicam(10mg bid) | Compound Qiyi Capsule：Coix lacryma-jobi L.[Poaceae;Semen Coicis] ; Astragalus mongholicus Bunge[Fabaceae;Radix Astragali seu Hedysari] ; Centipede[Scolopendridae; Scolopendra] ; Tripterygium wilfordii[Celastraceae; Radix Tripterygii Wilfordii],；Astragalus mongholicus Bunge[Fabaceae;Radix Astragali seu Hedysari],n Qingre Chubi Capsules：Astragalus mongholicus Bunge[Fabaceae;Radix Astragali seu Hedysari],n ; Gardenia jasminoides J.Ellis[Rubiaceae;Fructus Gardeniae] ; Prunus persica (L.) Batsch[Rosaceae;Semen Persicae] ; Clematis chinensis Osbeck[Ranunculaceae;Radix Clematidis]; etc. |  |
| Observation on effect of integrated Chinese medicine and western medicine on 32 pafients with rheumatoid arthritis[28] | 2014 | MTX(12.5mg qw)+SSZ(1g bid)+Meloxicam*(7.5mg bid)+Diclofenac Sodium Sustained Release Capsules(25mg qd)+Yiyiren Soup | MTX(12.5mg qw)+SSZ(1g bid)+Meloxicam*(7.5mg bid)+Diclofenac Sodium Sustained Release Capsules(25mg qd) | Coix lacryma-jobi L.[Poaceae;Semen Coicis], 20g; Atractylodes lancea (Thunb.) DC.[Asteraceae;Rhizoma Atractylodis], 12g; Glycyrrhiza glabra[Fabaceae; Radix Glycyrrhizae], 6g; Hansenia weberbaueriana (Fedde ex H.Wolff) Pimenov & Kljuykov [Apiaceae;Rhizoma et Radix Notopterygii], 10g; Angelica dahurica (Hoffm.) Benth. & Hook.f. ex Franch. & Sav. [Apiaceae;Radix Angelicae Pubescentis], 10g; Saposhnikovia divaricata (Turcz. ex Ledeb.) Schischk. [Apiaceae;Radix Saposhnikoviae], 10g; Ephedra sinica Stapf [Ephedraceae;Herba Ephedrae], 6g; Neolitsea cassia (L.) Kosterm.[Lauraceae;Ramulus Cinnamomi], 10g; Aconitum carmichaeli Debeaux [Ranunculaceae;Radix Aconiti], 10g; Angelica sinensis (Oliv.) Diels[Apiaceae;Radix Angelicae Sinensis], 8g; Conioselinum anthriscoides ‘Chuanxiong’[Apiaceae;Rhizoma Ligustici], 8g |  |
| Clinical observation on treating rheumatoid arthritis by Bushen Huoxue therapy[29] | 2015 | MTX(10-15mg qw)+LEF(20mg qd)+Aceclofenac Tablets(100mg bid)+Rabeprazole enteric-coated capsules(20mg qd)+CMC* | MTX(10-15mg qw)+LEF(20mg qd)+Aceclofenac Tablets(100mg bid)+Rabeprazole enteric-coated capsules(20mg qd) | Angelica dahurica (Hoffm.) Benth. & Hook.f. ex Franch. & Sav. [Apiaceae;Radix Angelicae Pubescentis], 20g; Taxillus chinensis (DC.) Danser[Loranthaceae; Herba Taxilli], 30g; Eucommia ulmoides Oliv. [Eucommiaceae; Cortex Eucommiae], 15g; Asarum sieboldii[Aristolochiaceae；Herba cum Radix Asari], 3g; Angelica sinensis (Oliv.) Diels[Apiaceae;Radix Angelicae Sinensis], 10g; Paeonia lactiflora Pall.[Paeoniaceae;Radix Paeoniae Alba], 20g; Glycyrrhiza glabra[Fabaceae; Radix Glycyrrhizae], 6g; Rehmannia glutinosa (Gaertn.) DC. [Orobanchaceae;Radix Rehmanniae Preparata], 15g; Conioselinum anthriscoides ‘Chuanxiong’[Apiaceae;Rhizoma Ligustici], 15g; Neolitsea cassia (L.) Kosterm.[Lauraceae;Ramulus Cinnamomi], 15g; Codonopsis pilosula[Campanulaceae; Radix Codonopsis], 15g; Gleditsia sinensis Lam. [Fabaceae; Spina Gleditsiae], 12g; Quanchong 5g; Centipede[Scolopendridae; Scolopendra], 2 pieces; Spatholobus suberectus Dunn[Fabaceae;Caulis Spatholobi], 30g |  |
| Clinical Observation on Yishen Qingluo Huoxue Decoction in Treatment of Intermingled Phlegm and Blood Stasis Type of Rheumatoid Arthritis[30] | 2015 | Yishen Qingluo Huoxue Decoction(bid) | MTX(10mg qw)+LEF(20mg qd) | Sophora flavescens Aiton[Fabaceae;Radix Sophorae Flavescentis], 9g; Sinomenium acutum[Menispermaceae; Caulis Sinomenii], 9g; Phellodendron amurense Rupr. [Rutaceae;Cortex Phellodendri], 9g; Dioscorea collettii var. hypoglauca (Palib.) S.J.Pei & C.T.Ting[Dioscoreaceae;Rhizome Dioscoreae Hypoglaucae], 10g; Astragalus mongholicus Bunge[Fabaceae;Radix Astragali seu Hedysari], 30g; Angelica sinensis (Oliv.) Diels[Apiaceae;Radix Angelicae Sinensis], 15g; Sargentodoxa cuneata (Oliv.) Rehder [Lardizabalaceae; Caulis Sargentodoxae], 15g; Spatholobus suberectus Dunn[Fabaceae;Caulis Spatholobi], 15g; Tripterygium wilfordii[Celastraceae; Radix Tripterygii Wilfordii], (decocted earlier)10g; Equus asinus L. [Equidae; Colla Corii Asini], 25g; Citrus × aurantium L.[Rutaceae;Pericarpium Citri Reticulatae], 9g; Taxillus chinensis (DC.) Danser[Loranthaceae; Herba Taxilli], 15g; Centipede[Scolopendridae; Scolopendra], 1 piece; Zaocys dhumnades(Cantor)[Natricinae;Zaocys dhumnades], 10g |  |
| Clinical observation of Yishen Qingluo Huoxue Decoction in treating rheumatoid arthritis with Mutual junction of phlegm and blood stasis[31] | 2015 | Yishen Qingluo Huoxue Decoction(bid) | LEF(10mg bid) | Astragalus mongholicus Bunge[Fabaceae;Radix Astragali seu Hedysari], 30g; Equus asinus L. [Equidae; Colla Corii Asini], 20g; Angelica sinensis (Oliv.) Diels[Apiaceae;Radix Angelicae Sinensis], 15g; Sargentodoxa cuneata (Oliv.) Rehder [Lardizabalaceae; Caulis Sargentodoxae], 15g; Spatholobus suberectus Dunn[Fabaceae;Caulis Spatholobi], 15g; Cuscuta chinensis Lam. [Convolvulaceae;Semen Cuscutae] 12g; Dioscorea collettii var. hypoglauca (Palib.) S.J.Pei & C.T.Ting[Dioscoreaceae;Rhizome Dioscoreae Hypoglaucae], 12g; Epimedium sagittatum (Siebold & Zucc.) Maxim. [Berberidaceae;Herba Epimedii], 12g; Sinomenium acutum[Menispermaceae; Caulis Sinomenii], 9g; Pinellia ternata (Thunb.) Makino [Araceae; Rhizoma Pinelliae], 9g; Sophora flavescens Aiton[Fabaceae;Radix Sophorae Flavescentis], 9g; Phellodendron amurense Rupr. [Rutaceae;Cortex Phellodendri], 9g |  |
| Observation on the curative effect of combined Chinese and Western medicine on rheumatoid arthritis[32] | 2015 | MTX(10mg qw)+Prednisone(8mg qd)+CMC*(bid) | MTX(10mg qw)+Prednisone(8mg qd) | Saposhnikovia divaricata (Turcz. ex Ledeb.) Schischk. [Apiaceae;Radix Saposhnikoviae], 30g; Gypsum [Mineral; Gypsum Fibrosum], 30g; Gardenia jasminoides J.Ellis[Rubiaceae;Fructus Gardeniae], 30g; Astragalus mongholicus Bunge[Fabaceae;Radix Astragali seu Hedysari], 30g; Citrus × aurantium L.[Rutaceae;Pericarpium Citri Reticulatae], 30g;Atractylodes macrocephala Koidz.[Asteraceae;Rhizoma Atractylodis Macrocephalae], 25g; Pogostemon cablin (Blanco) Benth. [Lamiaceae; Pogostemon Cablin (Blanco) Benth.] 15g; Actaea cimicifuga L. [Ranunculaceae; Rhizoma Cimicifugae], 15g; Bupleurum falcatum L. [Apiaceae; Radix Bupleuri], 15g; Codonopsis pilosula[Campanulaceae; Radix Codonopsis], 25g; Angelica sinensis (Oliv.) Diels[Apiaceae;Radix Angelicae Sinensis], 20g; Glycyrrhiza glabra[Fabaceae; Radix Glycyrrhizae], 10g |  |
| Clinical observation on the treatment of rheumatoid arthritis by adding or subtracting Judanxi gout prescription combined with methotrexate[33] | 2015 | MTX(10mg qw)+NSAIDs*+JuDanxi gout prescription(3-4g, bid or tid) | MTX(10mg qw)+NSAIDs* | Atractylodes lancea (Thunb.) DC.[Asteraceae;Rhizoma Atractylodis], 10g; Phellodendron amurense Rupr. [Rutaceae;Cortex Phellodendri], 10g; Stephania tetrandra[Menispermaceae; Radix Stephaniae Tetrandrae], 10g; Conioselinum anthriscoides ‘Chuanxiong’[Apiaceae;Rhizoma Ligustici], 10g; Hansenia weberbaueriana (Fedde ex H.Wolff) Pimenov & Kljuykov [Apiaceae;Rhizoma et Radix Notopterygii], 10g; Angelica dahurica (Hoffm.) Benth. & Hook.f. ex Franch. & Sav.[Apiaceae;Radix Angelicae Dahuricae], 6g; Clematis chinensis Osbeck[Ranunculaceae;Radix Clematidis], 10g; Neolitsea cassia (L.) Kosterm.[Lauraceae;Ramulus Cinnamomi], 5g; Sinapis alba L. [Brassicaceae;Semen sinapis.], 10g; Prunus persica (L.) Batsch[Rosaceae;Semen Persicae], 10g; Carthamus tinctorius[Asteraceae; Flos Carthami], 10g; Zaocys dhumnades(Cantor)[Natricinae;Zaocys dhumnades], 10g; Sigesbeckia orientalis[Asteraceae; Herba Siegesbeckiae], 30g; Medicated leaven[Massa Medicata Fermentata], 10g |  |
| Zhuifeng Tougu Capsules combined with Methotmxate on the Treatment of Rheumatoid Arthritis for 80 Cases[34] | 2015 | MTX(15mg qw)+Zhuifeng Tougu Capsules(4# bid)+NSAIDs* | MTX(15mg qw)+NSAIDs* | Aconitum carmichaeli Debeaux [Ranunculaceae;Radix Aconiti]; Aconitum kusnezoffii Rchb.[Ranunculaceae;Radix Aconiti Kusnezoffii]; Cyperus rotundus L. [Cyperaceae; Rhizoma Cyperi];Conioselinum anthriscoides ‘Chuanxiong’[Apiaceae;Rhizoma Ligustici]; Ephedra sinica Stapf [Ephedraceae;Herba Ephedrae],;Angelica sinensis (Oliv.) Diels[Apiaceae;Radix Angelicae Sinensis];Arisaema heterophyllum Blume [Araceae; Rhizoma Arisaematis];Angelica dahurica (Hoffm.) Benth. & Hook.f. ex Franch. & Sav.[Apiaceae;Radix Angelicae Dahuricae];Commiphora myrrha[Burseraceae; Resina Commiphorae];Frankincense[Burseraceae; Olibanum];Pheretima asiatica Michaelsen[Megascolecidae;Lumbricus], | *：Addition of low-dose NSAIDs for those with significant pain |
| Clinical Effect of Duhuo Jisheng Decoction Combined with Meloxicam in Treatment of Rheumatoid Arthritis[35] | 2016 | Meloxicam(15mg qd)+Duhuo Jisheng Decoction(bid) | Meloxicam(15mg qd) | Angelica dahurica (Hoffm.) Benth. & Hook.f. ex Franch. & Sav. [Apiaceae;Radix Angelicae Pubescentis], 10g; Eucommia ulmoides Oliv. [Eucommiaceae; Cortex Eucommiae], 15g; Achyranthes bidentata Blume [Amaranthaceae;Radix Achyranthis Bidentatae], 10g; Asarum sieboldii[Aristolochiaceae；Herba cum Radix Asari], 6g; Panax ginseng C.A.Mey.[Araliaceae;Radix Ginseng], 10g; Taxillus chinensis (DC.) Danser[Loranthaceae; Herba Taxilli], 20g; Saposhnikovia divaricata (Turcz. ex Ledeb.) Schischk. [Apiaceae;Radix Saposhnikoviae], 10g; Neolitsea cassia[Lauraceae; Cortex Cinnamomi], 10g; Angelica sinensis (Oliv.) Diels[Apiaceae;Radix Angelicae Sinensis], 10g; Gentiana macrophylla Pall.[Gentianaceae;Radix Gentianae Macrophyllae], 10g; Conioselinum anthriscoides ‘Chuanxiong’[Apiaceae;Rhizoma Ligustici], 10g; Glycyrrhiza glabra[Fabaceae; Radix Glycyrrhizae], 6g; Paeonia lactiflora Pall.[Paeoniaceae;Radix Paeoniae Alba], 10g |  |
| A clinical study on treatment of rheumatoid arthritis by Juanbi Yanggan Yishen decoction[36] | 2016 | LEF(20mg qd)+Loxoprofen Sodium Tablets(60mg tid)+Juanbi Yanggan Yishen decoction(bid) | LEF(20mg qd) )+Loxoprofen Sodium Tablets(60mg tid) | Astragalus mongholicus Bunge[Fabaceae;Radix Astragali seu Hedysari], 30g; Angelica sinensis (Oliv.) Diels[Apiaceae;Radix Angelicae Sinensis], 20g; Conioselinum anthriscoides ‘Chuanxiong’[Apiaceae;Rhizoma Ligustici], 15g; Shudi 15g; Saposhnikovia divaricata (Turcz. ex Ledeb.) Schischk. [Apiaceae;Radix Saposhnikoviae], 15g; Hansenia weberbaueriana (Fedde ex H.Wolff) Pimenov & Kljuykov [Apiaceae;Rhizoma et Radix Notopterygii], 10g;Angelica dahurica (Hoffm.) Benth. & Hook.f. ex Franch. & Sav. [Apiaceae;Radix Angelicae Pubescentis], 10g; Neolitsea cassia (L.) Kosterm.[Lauraceae;Ramulus Cinnamomi], 15g; Cucumis melo L. [Cucurbitaceae; Retinervus Luffae Fructus], 15g; Gentiana macrophylla Pall.[Gentianaceae;Radix Gentianae Macrophyllae], 15g; Piper kadsura[Piperaceae; Caulis Piperis Kadsurae], 12g; Trachelospermum jasminoides (Lindl.) Lem. [Apocynaceae; Caulis Trachelospermi], 20g; Uncaria rhynchophylla (Miq.) Miq. [Rubiaceae; Ramulus Uncariae Cum Uncis] , 20g; Spatholobus suberectus Dunn[Fabaceae;Caulis Spatholobi], 30g; Paeonia lactiflora Pall.[Paeoniaceae;Radix Paeoniae Alba], 30g; Paeonia lactiflora Pall. [Paeoniaceae;Radix Paeoniae Rubra], 15g;Epimedium sagittatum (Siebold & Zucc.) Maxim. [Berberidaceae;Herba Epimedii], 15g; Taxillus chinensis (DC.) Danser[Loranthaceae; Herba Taxilli], 12g; Pheretima asiatica Michaelsen[Megascolecidae;Lumbricus], 12g; Buthus martensii Karsch[Scorpiones;Scorpio], 10g; Curcuma longa[Zingiberaceae; Rhizoma Curcumae Longae], 10g; Coix lacryma-jobi L.[Poaceae;Semen Coicis],10g; Glycyrrhiza glabra[Fabaceae; Radix Glycyrrhizae], 10g |  |
| Clinical observation of Liuwei Dihuang combined with Siwu Decoction combined with methotrexate in the treatment of rheumatoid arthritis[37] | 2016 | MTX(10mg qw)+Liuwei Dihuang combined with Siwu Decoction(bid) | MTX(10mg qw) | Rehmannia glutinosa (Gaertn.) DC. [Orobanchaceae;Radix Rehmanniae Preparata], 15g; Astragalus mongholicus Bunge[Fabaceae;Radix Astragali seu Hedysari],n 15g; Shanyu 12g; Paeonia × suffruticosa [Paeoniaceae;Cortex Moutan Radicis], 12g; Poria[Polyporaceae; Sclerotium Poriae Cocos], 12g; Coptis chinensis Franch.[Ranunculaceae;Rhizoma Coptidis], 12g; Dioscorea oppositifolia[Dioscoreaceae; Rhizoma Dioscoreae], 10g; Chrysanthemum × morifolium (Ramat.) Hemsl.[Asteraceae;Flos Chrysanthemi], 10g; Alisma plantago-aquatica subsp. orientale (Sam.) Sam.[Alismataceae;Rhizoma Alismatis], 10g; Angelica sinensis (Oliv.) Diels[Apiaceae;Radix Angelicae Sinensis], 10g |  |
| Effect of removing wind, dehumidifying and tonifying kidney in the treatment of senile rheumatoid arthritis and the influence of antibody levels against cyclic citrulline polypeptide, rheumatoid factor, erythrocyte sedimentation rate, C-reactive protein and keratin[38] | 2016 | LEF(20mg qd)+Prednisone(7.5mg qd)+CMC*(bid) | LEF(20mg qd)+Prednisone(7.5mg qd) | Zaocys dhumnades(Cantor)[Natricinae;Zaocys dhumnades], 12g; Taxillus chinensis (DC.) Danser[Loranthaceae; Herba Taxilli], 20g; Buthus martensii Karsch[Scorpiones;Scorpio], 6g; Lonicera japonica Thunb. [Caprifoliaceae;Caulis Lonicerae], 30g; Coix lacryma-jobi L.[Poaceae;Semen Coicis], 30g; Atractylodes lancea (Thunb.) DC.[Asteraceae;Rhizoma Atractylodis],12g; Astragalus mongholicus Bunge[Fabaceae;Radix Astragali seu Hedysari], 30g; Saposhnikovia divaricata (Turcz. ex Ledeb.) Schischk. [Apiaceae;Radix Saposhnikoviae], 12g; Conioselinum anthriscoides ‘Chuanxiong’[Apiaceae;Rhizoma Ligustici], 12g; Epimedium sagittatum (Siebold & Zucc.) Maxim. [Berberidaceae;Herba Epimedii], 10g; Rehmannia glutinosa (Gaertn.) DC. [Orobanchaceae;Radix Rehmanniae Preparata], 10g; Psoralea fructus[Fabaceae; Fructus Psoraliae], 10g |  |
| Clinical Observation of 35 Cases of Rheumatoid Arthritis of Damp Heat Type Treated with Simiao Podwer combined with Xuanbi Decoction and Western Medicine[39] | 2016 | MTX(5mg-10mg qd-biw)+LEF(20mg qd)+ Simiao Podwer combined with Xuanbi Decoction*(250mL bid) | MTX(5mg-10mg qd-biw)+LEF(20mg qd) | Forsythia suspensa (Thunb.) Vahl [Oleaceae; Fructus Forsythiae], 10g; Phellodendron amurense Rupr. [Rutaceae;Cortex Phellodendri], 10g; Paeonia lactiflora Pall.[Paeoniaceae;Radix Paeoniae Alba], 10g; Stephania tetrandra[Menispermaceae; Radix Stephaniae Tetrandrae], 15g; Coix lacryma-jobi L.[Poaceae;Semen Coicis], 15g; Grona styracifolia (Osbeck) H.Ohashi & K.Ohashi [Fabaceae; Herba Lysimachiae], 10g; Lonicera japonica [Caprifoliaceae;Flos Lonicerae], 10g; Clematis chinensis Osbeck[Ranunculaceae;Radix Clematidis], 15g; Viola philippica Cav. [Violaceae; Herba Violae], 10g; Agkistrodon[Deinagkistrodon;Bungarus], 10g; Patrinia scabiosifolia Link [Caprifoliaceae; Herba Patriniae], 10g; Saposhnikovia divaricata (Turcz. ex Ledeb.) Schischk. [Apiaceae;Radix Saposhnikoviae], 10g; Lonicera japonica Thunb. [Caprifoliaceae;Caulis Lonicerae], 10g; Morus alba[Moraceae; Ramulus Mori], 10g; Pheretima asiatica Michaelsen[Megascolecidae;Lumbricus], 6g; Glycyrrhiza glabra[Fabaceae; Radix Glycyrrhizae], 6g |  |
| Clinical observation of 35 cases of Rheumatoid arthritis of Kidney Qi deficiency and Cold type treated by Yishen Juanbi Capsule combined with Western medicine[40] | 2016 | MTX(10mg qw)+LEF(10mg qd)+Celebrex*(200mg qd)+Yishen Juanbi Capsule(2g tid) | MTX(10mg qw)+LEF(10mg qd)+Celebrex*(200mg qd) | Ant ; Panax ginseng C.A.Mey.[Araliaceae;Radix Ginseng],; Aconitum carmichaeli Debeaux [Ranunculaceae;Radix Aconiti] ; Neolitsea cassia (L.) Kosterm.[Lauraceae;Ramulus Cinnamomi] ; Atractylodes lancea (Thunb.) DC.[Asteraceae;Rhizoma Atractylodis],; Phellodendron amurense Rupr. [Rutaceae;Cortex Phellodendri] ; Coix lacryma-jobi L.[Poaceae;Semen Coicis] ; Alisma plantago-aquatica subsp. orientale (Sam.) Sam.[Alismataceae;Rhizoma Alismatis] ; Centipede[Scolopendridae; Scolopendra] ; Zaocys dhumnades(Cantor)[Natricinae;Zaocys dhumnades] ; Lycopodium japonicum[Lycopodiaceae; Herba Lycopodii]; Strychnos nux-vomica L. [Loganiaceae; Semen Strychni]; Spatholobus suberectus Dunn[Fabaceae;Caulis Spatholobi] ; Garden Balsam Stem [Euphorbiaceae; Speranskia tuberculata (Bunge) Baill] ; Salvia miltiorrhiza Bunge[Lamiaceae; Radix Salviae Miltiorrhizae], | *：Add in when scores of visual analogue scale (VAS) ＞6 |
| Analysis and evaluation of the curative effect of Self-prepared Chinese medicine combined with anti-rheumatism medicine on active rheumatoid arthritis[41] | 2016 | LEF(20mg qd)+CMC* | LEF(20mg qd) | Aconitum carmichaeli Debeaux [Ranunculaceae;Radix Aconiti], 8g; Neolitsea cassia (L.) Kosterm.[Lauraceae;Ramulus Cinnamomi], 8g; Angelica sinensis (Oliv.) Diels[Apiaceae;Radix Angelicae Sinensis], 10g; Shengdi15g; Paeonia lactiflora Pall.[Paeoniaceae;Radix Paeoniae Alba], 20g; Anemarrhena asphodeloides[Liliaceae; Rhizoma Anemarrhenae], 20g; Lonicera japonica Thunb. [Caprifoliaceae;Caulis Lonicerae], 20g; Pheretima asiatica Michaelsen[Megascolecidae;Lumbricus], 12g; Bombyx mori Linnaeus[Bombyx Linnaeus;Bombyx Batryticatus], 12g; Zaocys dhumnades(Cantor)[Natricinae;Zaocys dhumnades], 10g; Glycyrrhiza glabra[Fabaceae; Radix Glycyrrhizae], 6g |  |
| Clinical research on treating rheumatoid arthritis by the Buyi Qixue, Qushi Tongluo therapy[42] | 2017 | LEF(10mg qd)+Aceclofenac Enteric-coated Tablets(100mg bid)+CMC(tid) | LEF(10mg qd)+Aceclofenac Enteric-coated Tablets(100mg bid) | Astragalus mongholicus Bunge[Fabaceae;Radix Astragali seu Hedysari], 60g; Neolitsea cassia (L.) Kosterm.[Lauraceae;Ramulus Cinnamomi], 20g; Paeonia lactiflora Pall.[Paeoniaceae;Radix Paeoniae Alba], 20g; Zingiber officinale[Zingiberaceae; Rhizoma Zingiberis], 10g; Ziziphus jujuba Mill. [Rhamnaceae; Fructus Jujubae], 10g; Glycyrrhiza glabra[Fabaceae; Radix Glycyrrhizae], 5g; Tripterygium wilfordii[Celastraceae; Radix Tripterygii Wilfordii], 15g; Spatholobus suberectus Dunn[Fabaceae;Caulis Spatholobi], 30g; Piper kadsura[Piperaceae; Caulis Piperis Kadsurae], 30g; Trachelospermum jasminoides (Lindl.) Lem. [Apocynaceae; Caulis Trachelospermi], 30g; Yinhuateng 30g |  |
| Effects of Hebi Formula on bone erosion in early rheumatoid arthritis of liver and spleen disorder type[43] | 2017 | *MTX(7.5mg qw)+Folic acid tablets(5mg qw)+*Diclofenac sodium double release Intestine-sol Capsule(75mg qd)+Hebi Formula(bid) | *MTX(7.5mg qw)+Folic acid tablets(5mg qw)+*Diclofenac sodium double release Intestine-sol Capsule(75mg qd) | Angelica sinensis (Oliv.) Diels[Apiaceae;Radix Angelicae Sinensis], 20g; Paeonia lactiflora Pall.[Paeoniaceae;Radix Paeoniae Alba], 30g; Atractylodes macrocephala Koidz.[Asteraceae;Rhizoma Atractylodis Macrocephalae], 15g; Atractylodes lancea (Thunb.) DC.[Asteraceae;Rhizoma Atractylodis],10g; Sinomenium acutum[Menispermaceae; Caulis Sinomenii], 20g; Sarcandra glabra (Thunb.) Nakai[Chloranthaceae;Herba Sarcandrae], 30g; Conioselinum anthriscoides ‘Chuanxiong’[Apiaceae;Rhizoma Ligustici], 20g; Saposhnikovia divaricata (Turcz. ex Ledeb.) Schischk. [Apiaceae;Radix Saposhnikoviae], 9g; Glycyrrhiza glabra[Fabaceae; Radix Glycyrrhizae], 30g | *: MTX may be increased by 2.5 mg every 2-4 weeks to 12.5 mg if well treated and tolerated; for those with a symptom score of 4 or more, add diclofenac sodium double-release enteric capsules 75 mg once daily |
| Effect of Modified Juanbi Decoction on Inflammatory Factors and Serum MMP-3，OPG and ＲANKL in Synovial Fluid of Patients with Wind-cold-wetness Type Rheumatoid Arthritis[44] | 2017 | MTX(10mg qw)+Meloxicam(15mg qd)+Juanbi Decoction*(100mL bid) | MTX(10mg qw)+Meloxicam(15mg qd) | Angelica dahurica (Hoffm.) Benth. & Hook.f. ex Franch. & Sav. [Apiaceae;Radix Angelicae Pubescentis], 15g; Hansenia weberbaueriana (Fedde ex H.Wolff) Pimenov & Kljuykov [Apiaceae;Rhizoma et Radix Notopterygii], 15g; Conioselinum anthriscoides ‘Chuanxiong’[Apiaceae;Rhizoma Ligustici], 15g; Frankincense[Burseraceae; Olibanum], 10g; Gentiana macrophylla Pall.[Gentianaceae;Radix Gentianae Macrophyllae], 10g; Aucklandia costus Falc. [Asteraceae; Radix Aucklandiae], 10g; Morus alba[Moraceae; Ramulus Mori], 10g; Piper kadsura[Piperaceae; Caulis Piperis Kadsurae], 10g; Angelica sinensis (Oliv.) Diels[Apiaceae;Radix Angelicae Sinensis], 20g; Neolitsea cassia (L.) Kosterm.[Lauraceae;Ramulus Cinnamomi], 12g; Glycyrrhiza glabra[Fabaceae; Radix Glycyrrhizae], 6g |  |
| Clinical Research on Liuwei Dihuang Decoction combined with Siwu Decoction Treatment of Rheumatoid Arthritis[45] | 2017 | MTX(10mg qw)+Liuwei Dihuang Decoction combined with Siwu Decoction(bid) | MTX(10mg qw) | Rehmannia glutinosa (Gaertn.) DC. [Orobanchaceae;Radix Rehmanniae Preparata], 30g; Astragalus mongholicus Bunge[Fabaceae;Radix Astragali seu Hedysari],n 15g; Shanyu 20g; Paeonia × suffruticosa [Paeoniaceae;Cortex Moutan Radicis], 10g; Poria[Polyporaceae; Sclerotium Poriae Cocos], 15g; Coptis chinensis Franch.[Ranunculaceae;Rhizoma Coptidis], 10g; Dioscorea oppositifolia[Dioscoreaceae; Rhizoma Dioscoreae], 20g; Chrysanthemum × morifolium (Ramat.) Hemsl.[Asteraceae;Flos Chrysanthemi], 10g; Alisma plantago-aquatica subsp. orientale (Sam.) Sam.[Alismataceae;Rhizoma Alismatis], 10g; Paeonia lactiflora Pall. [Paeoniaceae;Radix Paeoniae Rubra], 15g; Angelica sinensis (Oliv.) Diels[Apiaceae;Radix Angelicae Sinensis], 10g |  |
| Clinical observation on 340 cases of Rheumatoid arthritis treated by Mayi Tongbi Capsule[46] | 2017 | Diclofenac Sodium Enteric-coated Sustained Release Capsules(75mg qd)+Mayi Tongbi Capsule(4# tid) | MTX(10mg qw)+Diclofenac Sodium Enteric-coated Sustained Release Capsules(75mg qd) | Ant ; Atractylodes macrocephala Koidz.[Asteraceae;Rhizoma Atractylodis Macrocephalae] ; Aconitum carmichaeli[Ranunculaceae; Radix Aconiti Lateralis Preparata] ; Neolitsea cassia (L.) Kosterm.[Lauraceae;Ramulus Cinnamomi]; etc. |  |
| Clinical Study on Jiawei Wutou Decoction in Treatment of Rheumatoid Arthritis[47] | 2017 | MTX(10mg qw)+LEF(20mg qd)+Jiawei Wutou Decoction(bid) | MTX(10mg qw)+LEF(20mg qd) | Aconitum carmichaeli Debeaux [Ranunculaceae;Radix Aconiti], 20g; Ephedra sinica Stapf [Ephedraceae;Herba Ephedrae], 6g; Astragalus mongholicus Bunge[Fabaceae;Radix Astragali seu Hedysari], 30g; Paeonia lactiflora Pall.[Paeoniaceae;Radix Paeoniae Alba], 20g; Glycyrrhiza glabra[Fabaceae; Radix Glycyrrhizae], 10g; Neolitsea cassia (L.) Kosterm.[Lauraceae;Ramulus Cinnamomi], 10g; Clematis chinensis Osbeck[Ranunculaceae;Radix Clematidis], 10g; Asarum sieboldii[Aristolochiaceae；Herba cum Radix Asari], 3g; Eupolyphaga[Corydidae;Eupolyphaga Seu Steleophaga], 10g; Morus alba[Moraceae; Ramulus Mori], 20g; Lycopodium japonicum[Lycopodiaceae; Herba Lycopodii], 20g; Angelica dahurica (Hoffm.) Benth. & Hook.f. ex Franch. & Sav. [Apiaceae;Radix Angelicae Pubescentis], 20g; Taxillus chinensis (DC.) Danser[Loranthaceae; Herba Taxilli], 20g; Reynoutria multiflora (Thunb.) Moldenke[Polygonaceae;Radix Polygoni Multiflori], 20g; Dipsacus asper[Caprifoliaceae; Radix Dipsaci], 15g |  |
| Clinical observation of Buyang Huanwu Decoction in treating phlegm and blood stasis type rheumatoid arthritis[48] | 2018 | MTX(15mg qw)+Celebrex(200mg qd)+Buyang Huanwu Decoction(qd) | MTX(15mg qw)+Celebrex(200mg qd) | Paeonia lactiflora Pall. [Paeoniaceae;Radix Paeoniae Rubra], 15g; Angelica sinensis (Oliv.) Diels[Apiaceae;Radix Angelicae Sinensis], 15g; Pheretima asiatica Michaelsen[Megascolecidae;Lumbricus], 10g; Astragalus mongholicus Bunge[Fabaceae;Radix Astragali seu Hedysari], 25g; Conioselinum anthriscoides ‘Chuanxiong’[Apiaceae;Rhizoma Ligustici], 10g; Arisaema erubescens[Araceae; Arisaemae cum Bile], 10g; Sinapis alba L. [Brassicaceae;Semen sinapis.], 10g; Buthus martensii Karsch[Scorpiones;Scorpio], 5g; Angelica dahurica (Hoffm.) Benth. & Hook.f. ex Franch. & Sav. [Apiaceae;Radix Angelicae Pubescentis], 15g; Hansenia weberbaueriana (Fedde ex H.Wolff) Pimenov & Kljuykov [Apiaceae;Rhizoma et Radix Notopterygii], 15g; Neolitsea cassia (L.) Kosterm.[Lauraceae;Ramulus Cinnamomi], 10g; Paeonia lactiflora Pall.[Paeoniaceae;Radix Paeoniae Alba], 15g; Glycyrrhiza glabra[Fabaceae; Radix Glycyrrhizae], 5g |  |
| Gulao Yukang Pills combined with methotrexate tablets in the treatment of 40 cases of rheumatoid arthritis[49] | 2018 | MTX(5mg, twice a week)+Gulao Yukang Pills(15# tid) | MTX(5mg, twice a week) | Cervus nippon Temminck [Cervidae; Cornu Cervi Degelatinatum] ; Equus asinus L. [Equidae; Colla Corii Asini] ; Buthus martensii Karsch[Scorpiones;Scorpio] ; Panax notoginseng (Burkill) F.H.Chen[Araliaceae;Radix Notoginseng]; Neolitsea cassia[Lauraceae; Cortex Cinnamomi] ; Coptis chinensis Franch.[Ranunculaceae;Rhizoma Coptidis] ; Ligustrum lucidum W.T.Aiton [Oleaceae; Fructus Ligustri Lucidi] ; Chinemys reevesii (Gray) [Testudinidae; Plastrum Testudinis] ; Trionyx sinensis Wiegmann [Trionychidae; Carapax Trionycis]; Drynaria roosii[Polypodiaceae; Rhizoma Drynariae] ; Spatholobus suberectus Dunn[Fabaceae;Caulis Spatholobi] ; Glycyrrhiza glabra[Fabaceae; Radix Glycyrrhizae], | *：Both groups were given folic acid tablets (manufactured by Tianjin Lisheng Pharmaceutical Co., Ltd., lot No. 1011023, 5mg per tablet) on day 2 of oral methotrexate tablets, 10mg each time, once daily, orally. |
| Clinical observation on the treatment of rheumatoid arthritis with heat and blood stasis by traditional Chinese medicine therapy of clearing heat and activating blood circulation[50] | 2018 | MTX*(7.5mg-15mg qw)+LEF(10mg qd)+Diclofenac Sodium Sustained Release Tablets*(75mg qd)+Simiao Yongan Decoction(bid) | MTX*(7.5mg-15mg qw)+LEF(10mg qd)+Diclofenac Sodium Sustained Release Tablets*(75mg qd) | Lonicera japonica Thunb. [Caprifoliaceae;Caulis Lonicerae], 30g; Angelica sinensis (Oliv.) Diels[Apiaceae;Radix Angelicae Sinensis], 30g; Scrophularia ningpoensis[Scrophulariaceae; Radix Scrophulariae], 20g; Glycyrrhiza glabra[Fabaceae; Radix Glycyrrhizae], 10g; Dioscorea nipponica Makino[Dioscoreaceae;Rhizoma Dioscoreae Nipponicae], 30g; Arctium lappa L. [Arctium lappa L.; Fructus Arctii], 15g; Bombyx mori Linnaeus[Bombyx Linnaeus;Bombyx Batryticatus], 10g; etc. | *：MTX 7.5mg/week initially, once/week and thereafter in weekly increments of 2.5mg to 15mg/week for maintenance treatment. Diclofenac sodium administered as needed (take with joint pain, stop with pain relief)。 |
| Wenyang Chushi, Huatan tongluo prescription combined with conventional therapy to treat 90 cases of cold and dampness bizu rheumatoid arthritis[51] | 2018 | Aceclofenac(0.1g bid)+Wenyang Chushi, Huatan tongluo prescription combined(tid) | Aceclofenac(0.1g bid)+Celebrex*(200mg qd) | Astragalus mongholicus Bunge[Fabaceae;Radix Astragali seu Hedysari], 30g; Neolitsea cassia (L.) Kosterm.[Lauraceae;Ramulus Cinnamomi], 15g; Paeonia lactiflora Pall. [Paeoniaceae;Radix Paeoniae Rubra], 15g; Paeonia lactiflora Pall.[Paeoniaceae;Radix Paeoniae Alba], 15g; Angelica sinensis (Oliv.) Diels[Apiaceae;Radix Angelicae Sinensis], 20g; Arisaema erubescens[Araceae; Arisaemae cum Bile], 15g; Zingiber officinale[Zingiberaceae; Rhizoma Zingiberis],10g; Bombyx mori Linnaeus[Bombyx Linnaeus;Bombyx Batryticatus], 15g; Cremastra appendiculata (D.Don) Makino[Orchidaceae;Pseudobulbus Cremastrae seu Pleiones], 15g; Sinapis alba L. [Brassicaceae;Semen sinapis.], 9g; Corydalis yanhusuo[Papaveraceae;Rhizoma Corydalis], 15g; Pheretima asiatica Michaelsen[Megascolecidae;Lumbricus], 15g; Lonicera japonica Thunb. [Caprifoliaceae;Caulis Lonicerae], 30g; Glycyrrhiza glabra[Fabaceae; Radix Glycyrrhizae], 6g; |  |
| Forty cases of rheumatoid arthritis were treated by renal differentiation[52] | 2018 | Diclofenac Sodium Sustained Release Capsules(50mg bid)+CMC(bid) | Diclofenac Sodium Sustained Release Capsules(50mg bid) | Damp-heat obstruction type: Gentiana macrophylla Pall.[Gentianaceae;Radix Gentianae Macrophyllae], 15g; Angelica dahurica (Hoffm.) Benth. & Hook.f. ex Franch. & Sav. [Apiaceae;Radix Angelicae Pubescentis], 15g; Stephania tetrandra[Menispermaceae; Radix Stephaniae Tetrandrae], 9g; Poria[Polyporaceae; Sclerotium Poriae Cocos], 15g; Coix lacryma-jobi L.[Poaceae;Semen Coicis], 30g; Lycium barbarum L. [Lycium barbarum L.; Lycii Cortex], 15g; Lonicera japonica Thunb. [Caprifoliaceae;Caulis Lonicerae], 30g; Dipsacus asper[Caprifoliaceae; Radix Dipsaci], 15g; Drynaria roosii[Polypodiaceae; Rhizoma Drynariae], 15g; Anemarrhena asphodeloides[Liliaceae; Rhizoma Anemarrhenae], 15g; Phellodendron amurense Rupr. [Rutaceae;Cortex Phellodendri], 15g; Frankincense[Burseraceae; Olibanum], 9g; Glycyrrhiza glabra[Fabaceae; Radix Glycyrrhizae], 6g. Kidney qi deficiency and cold type：Epimedium sagittatum (Siebold & Zucc.) Maxim. [Berberidaceae;Herba Epimedii], 15g; Curculigo orchioides Gaertn.[Hypoxidaceae;Rhizoma Curculigins], 12g; Angelica dahurica (Hoffm.) Benth. & Hook.f. ex Franch. & Sav. [Apiaceae;Radix Angelicae Pubescentis], 15g; Hansenia weberbaueriana (Fedde ex H.Wolff) Pimenov & Kljuykov [Apiaceae;Rhizoma et Radix Notopterygii], 9g; Neolitsea cassia (L.) Kosterm.[Lauraceae;Ramulus Cinnamomi], 15g; Dipsacus asper[Caprifoliaceae; Radix Dipsaci], 15g; Drynaria roosii[Polypodiaceae; Rhizoma Drynariae], 15g; Clematis chinensis Osbeck[Ranunculaceae;Radix Clematidis], 15g; Psoralea fructus[Fabaceae; Fructus Psoraliae], 15g; Achyranthes bidentata Blume [Amaranthaceae;Radix Achyranthis Bidentatae], 15g; Lycopodium japonicum[Lycopodiaceae; Herba Lycopodii], 30g; Saposhnikovia divaricata (Turcz. ex Ledeb.) Schischk. [Apiaceae;Radix Saposhnikoviae], 10g; Aconitum carmichaeli[Ranunculaceae; Radix Aconiti Lateralis Preparata], 9g; Glycyrrhiza glabra[Fabaceae; Radix Glycyrrhizae], 6g. Blood stasis type: Eucommia ulmoides Oliv. [Eucommiaceae; Cortex Eucommiae], 15g; Dipsacus asper[Caprifoliaceae; Radix Dipsaci], 15g; Deerhorn glue[Cervidae;Colla Corni Cervi], 10g; Cyperus rotundus L. [Cyperaceae; Rhizoma Cyperi], 15g; Psoralea fructus[Fabaceae; Fructus Psoraliae], 15g; Drynaria roosii[Polypodiaceae; Rhizoma Drynariae], 15g; Salvia miltiorrhiza Bunge[Lamiaceae; Radix Salviae Miltiorrhizae], 20g; Reynoutria multiflora (Thunb.) Moldenke[Polygonaceae;Radix Polygoni Multiflori], 15g; Frankincense[Burseraceae; Olibanum], 9g; Commiphora myrrha[Burseraceae; Resina Commiphorae], 9g; Angelica dahurica (Hoffm.) Benth. & Hook.f. ex Franch. & Sav. [Apiaceae;Radix Angelicae Pubescentis], 15g; Spatholobus suberectus Dunn[Fabaceae;Caulis Spatholobi], 30g; Achyranthes bidentata Blume [Amaranthaceae;Radix Achyranthis Bidentatae], 15g; Angelica sinensis (Oliv.) Diels[Apiaceae;Radix Angelicae Sinensis], 15g; Paeonia lactiflora Pall. [Paeoniaceae;Radix Paeoniae Rubra], 12g. |  |
| Observation on the curative effect of combined Chinese and Western medicine on rheumatoid arthritis[53] | 2018 | MTX(10mg qw)+Celebrex*(200mg qd)+Tenglong Decoction combined with Siwu Decoction(bid) | MTX(10mg qw)+Celebrex*(200mg qd) | Piper kadsura[Piperaceae; Caulis Piperis Kadsurae], 12g; Sinomenium acutum[Menispermaceae; Caulis Sinomenii], 12g; Spatholobus suberectus Dunn[Fabaceae;Caulis Spatholobi], 12g; Yinhuateng 12g; Saposhnikovia divaricata (Turcz. ex Ledeb.) Schischk. [Apiaceae;Radix Saposhnikoviae], 10g; Dioscorea nipponica Makino[Dioscoreaceae;Rhizoma Dioscoreae Nipponicae], 10g; Sinapis alba L. [Brassicaceae;Semen sinapis.], 10g; Buthus martensii Karsch[Scorpiones;Scorpio], 6g; Angelica sinensis (Oliv.) Diels[Apiaceae;Radix Angelicae Sinensis], 15g; Arisaema erubescens[Araceae; Arisaemae cum Bile], 10g; Conioselinum anthriscoides ‘Chuanxiong’[Apiaceae;Rhizoma Ligustici], 8g; Paeonia lactiflora Pall.[Paeoniaceae;Radix Paeoniae Alba], 10g; Prunus persica (L.) Batsch[Rosaceae;Semen Persicae], 9g; Carthamus tinctorius[Asteraceae; Flos Carthami], 6g | *：A COX-2 inhibitor that is tapered off after symptoms resolved |
| The clinical effect of traditional Chinese medicine combined with antirheumatic drugs on rheumatoid arthritis[54] | 2018 | MTX(10mg qw)+HCQ*(0.4g qd)+Celebrex(200mg qd)+Juanbi Pills(7-8# tid)+Zushima tablet  (3# tid) | MTX(10mg qw)+HCQ*(0.4g qd)+Celebrex(200mg qd) | Rehmannia glutinosa (Gaertn.) DC. [Orobanchaceae;Radix Rehmanniae Preparata] ; Epimedium sagittatum (Siebold & Zucc.) Maxim. [Berberidaceae;Herba Epimedii] ; Clematis chinensis Osbeck[Ranunculaceae;Radix Clematidis] ; Cibotium barometz (L.) J.Sm.[Cyatheaceae;Rhizoma Cibotii] ; Anemarrhena asphodeloides[Liliaceae; Rhizoma Anemarrhenae] ; Lycopodium japonicum[Lycopodiaceae; Herba Lycopodii] ; Drynaria roosii[Polypodiaceae; Rhizoma Drynariae]; etc. | *：First dose 0.4g/dose in divided doses, when the efficacy no longer improves, the dose is reduced to 0.2g |
| Short-term and Long-term Effects of Applying Traditional Chinese Medicine Syndrome Differentiation Combined with Methotrexate in the Treatment of Rheumatoid Arthritis[55] | 2018 | MTX(7.5mg qw)+CMC(bid) | MTX(7.5mg qw) | Wind-cold-dampness syndrome：Clematis chinensis Osbeck[Ranunculaceae;Radix Clematidis], 15g; Hansenia weberbaueriana (Fedde ex H.Wolff) Pimenov & Kljuykov [Apiaceae;Rhizoma et Radix Notopterygii], 9g; Neolitsea cassia (L.) Kosterm.[Lauraceae;Ramulus Cinnamomi], 9g; Commiphora myrrha[Burseraceae; Resina Commiphorae], 9g; Conioselinum anthriscoides ‘Chuanxiong’[Apiaceae;Rhizoma Ligustici], 9g; Gentiana macrophylla Pall.[Gentianaceae;Radix Gentianae Macrophyllae], 9g; Angelica dahurica (Hoffm.) Benth. & Hook.f. ex Franch. & Sav. [Apiaceae;Radix Angelicae Pubescentis], 9g; Frankincense[Burseraceae; Olibanum], 9g; Angelica sinensis (Oliv.) Diels[Apiaceae;Radix Angelicae Sinensis], 9g. Wind-heat-dampness syndrome: Morus alba[Moraceae; Ramulus Mori], 20g; Lonicera japonica Thunb. [Caprifoliaceae;Caulis Lonicerae], 20g; Paeonia lactiflora Pall.[Paeoniaceae;Radix Paeoniae Alba], 15g; Stephania tetrandra[Menispermaceae; Radix Stephaniae Tetrandrae], 12g; Erythrina indica Lam. [Leguminosae; Cortex Erythrinae] , 12g; Anemarrhena asphodeloides[Liliaceae; Rhizoma Anemarrhenae], 10g; Saposhnikovia divaricata (Turcz. ex Ledeb.) Schischk. [Apiaceae;Radix Saposhnikoviae], 9g; Neolitsea cassia (L.) Kosterm.[Lauraceae;Ramulus Cinnamomi], 6g. of Phlegm and blood stasis syndrome：Codonopsis pilosula[Campanulaceae; Radix Codonopsis], 15g; Taxillus chinensis (DC.) Danser[Loranthaceae; Herba Taxilli], 12g; Gentiana macrophylla Pall.[Gentianaceae;Radix Gentianae Macrophyllae], 12g; Rehmannia glutinosa (Gaertn.) DC. [Orobanchaceae;Radix Rehmanniae Preparata], 12g; Poria[Polyporaceae; Sclerotium Poriae Cocos], 12g; Paeonia lactiflora Pall.[Paeoniaceae;Radix Paeoniae Alba], 12g; Eucommia ulmoides Oliv. [Eucommiaceae; Cortex Eucommiae], 10g; Angelica sinensis (Oliv.) Diels[Apiaceae;Radix Angelicae Sinensis], 10g; Achyranthes bidentata Blume [Amaranthaceae;Radix Achyranthis Bidentatae], 10g; Conioselinum anthriscoides ‘Chuanxiong’[Apiaceae;Rhizoma Ligustici], 10g; Angelica dahurica (Hoffm.) Benth. & Hook.f. ex Franch. & Sav. [Apiaceae;Radix Angelicae Pubescentis], 9g; Saposhnikovia divaricata (Turcz. ex Ledeb.) Schischk. [Apiaceae;Radix Saposhnikoviae], 9g; Neolitsea cassia[Lauraceae; Cortex Cinnamomi], 3g; Asarum sieboldii[Aristolochiaceae; Herba cum Radix Asari], 3g. Kidney-Yang deficiency syndrome: Wooly datvhmanspipe herb [Aristolochiaceae;Herba Aristolochiae Mollissimae], 15g; Atractylodes macrocephala Koidz.[Asteraceae;Rhizoma Atractylodis Macrocephalae], 15g; Epimedium sagittatum (Siebold & Zucc.) Maxim. [Berberidaceae;Herba Epimedii], 15g; Clematis chinensis Osbeck[Ranunculaceae;Radix Clematidis], 15g; Gynochthodes officinalis (F.C.How) Razafim. [Rubiaceae; Radix Morindae Officinalis], 12g; Achyranthes bidentata Blume [Amaranthaceae;Radix Achyranthis Bidentatae], 12g; Dioscorea oppositifolia[Dioscoreaceae; Rhizoma Dioscoreae], 12g; Poria[Polyporaceae; Sclerotium Poriae Cocos], 12g; Cibotium barometz (L.) J.Sm.[Cyatheaceae;Rhizoma Cibotii], 12g; Aconitum carmichaeli[Ranunculaceae; Radix Aconiti Lateralis Preparata], 9g; Shanyu 9g; Neolitsea cassia (L.) Kosterm.[Lauraceae;Ramulus Cinnamomi], 9g. |  |
| Clinical Observation of Baihu Plus Guizhi Decoction Combined with Western Medicine  in Treating Rheumatoid Arthritis with Rheumatic Heat Arthralgia Syndrome[56] | 2019 | MTX(15mg qw)+Meloxicam*(7.5mg qd)+Baihu Plus Guizhi Decoction(bid) | MTX(15mg qw)+Meloxicam*(7.5mg qd) | Gypsum [Mineral; Gypsum Fibrosum], 30g; Anemarrhena asphodeloides[Liliaceae; Rhizoma Anemarrhenae], 20g; Non-glutinous rice [Gramineae; Oryza sativa L.], 20g; Neolitsea cassia (L.) Kosterm.[Lauraceae;Ramulus Cinnamomi], 6g; Glycyrrhiza glabra[Fabaceae; Radix Glycyrrhizae], 6g; Angelica sinensis (Oliv.) Diels[Apiaceae;Radix Angelicae Sinensis], 10g; Rehmannia glutinosa[Orobanchaceae; Radix Rehmanniae], 50g; Prunus armeniaca L. [Rosaceae; Semen Armeniacae Amarum], 12g; Coix lacryma-jobi L.[Poaceae;Semen Coicis], 12g; Clematis chinensis Osbeck[Ranunculaceae;Radix Clematidis], 12g; Arnebia euchroma (Royle ex Benth.) I.M.Johnst. [Boraginaceae; Radix Lithospermi], 30g; Paeonia lactiflora Pall. [Paeoniaceae;Radix Paeoniae Rubra], 30g | *：A COX-2 inhibitor, taken only for the first 15 days after enrollment |
| Clinical study of Guikun Fengshi Mixture combined with methotrexate in the treatment of rheumatoid arthritis[57] | 2019 | MTX(7.5-20mg qw)+*Celebrex(200mg qd) or Meloxicam(7.5mg qd)+Guikun Fengshi Mixture(200ml qd) | MTX(7.5-20mg qw)+Celebrex(200mg qd) or Meloxicam(7.5mg qd) | Neolitsea cassia (L.) Kosterm.[Lauraceae;Ramulus Cinnamomi] ; Tripterygium hypoglaucum (H.Lév.) Hutch. [Celastraceae; Tripterygium hypoglaucum（Devl.）Hutch.] ; Paeonia lactiflora Pall.[Paeoniaceae;Radix Paeoniae Alba] ; Spatholobus suberectus Dunn[Fabaceae;Caulis Spatholobi] ; Zaocys dhumnades(Cantor)[Natricinae;Zaocys dhumnades] ; Coix lacryma-jobi L.[Poaceae;Semen Coicis] ; Zingiber officinale[Zingiberaceae; Rhizoma Zingiberis]; etc. | *: Patients in both groups were given one NSAIDs drug (Cilazep 0.2g or meloxicam tablets/capsules 7.5mg/dose) orally at baseline |
| Clinical efficacy of the Guizhi Shaoyao Zhimu Decoction on rheumatoid arthritis of the Fenghan Shibi type[58] | 2019 | MTX(10mg qw)+Guizhi Shaoyao Zhimu Decoction(bid) | *MTX(10mg qw) | Paeonia lactiflora Pall.[Paeoniaceae;Radix Paeoniae Alba], 12g; Anemarrhena asphodeloides[Liliaceae; Rhizoma Anemarrhenae], 12g; Neolitsea cassia (L.) Kosterm.[Lauraceae;Ramulus Cinnamomi], 9g; Paeonia lactiflora Pall. [Paeoniaceae;Radix Paeoniae Rubra], 9g; Ephedra sinica Stapf [Ephedraceae;Herba Ephedrae], 9g; Atractylodes macrocephala Koidz.[Asteraceae;Rhizoma Atractylodis Macrocephalae], 9g; Saposhnikovia divaricata (Turcz. ex Ledeb.) Schischk. [Apiaceae;Radix Saposhnikoviae], 9g; Glycyrrhiza glabra[Fabaceae; Radix Glycyrrhizae], 6g; Aconitum carmichaeli[Ranunculaceae; Radix Aconiti Lateralis Preparata], 6g; Zingiber officinale[Zingiberaceae; Rhizoma Zingiberis], 3 pieces | *: The dose may be increased by 2.5 mg every 2-4 weeks to a maximum of 15-20 mg, depending on the patient's treatment outcome and tolerance |
| Clinical Observation on Treating 41 Cases of Rheumatoid Arthritis of Mixed Heat and Cold Type with Guizhi Shaoyao Zhimu Decoction Combined with Methotrexate[59] | 2019 | MTX(7.5mg qw)+*Celebrex(100mg qd)+Guizhi Shaoyao Zhimu Decoction(bid) | MTX(7.5mg qw)+*Celebrex(100mg qd) | Neolitsea cassia (L.) Kosterm.[Lauraceae;Ramulus Cinnamomi], 15g; Anemarrhena asphodeloides[Liliaceae; Rhizoma Anemarrhenae], 10-15g; Paeonia lactiflora Pall.[Paeoniaceae;Radix Paeoniae Alba], 10-30g; Ephedra sinica Stapf [Ephedraceae;Herba Ephedrae], 10g; Zingiber officinale[Zingiberaceae; Rhizoma Zingiberis],10g; Saposhnikovia divaricata (Turcz. ex Ledeb.) Schischk. [Apiaceae;Radix Saposhnikoviae], 10g; Atractylodes macrocephala Koidz.[Asteraceae;Rhizoma Atractylodis Macrocephalae], 10g; Aconitum carmichaeli[Ranunculaceae; Radix Aconiti Lateralis Preparata], 10-30g; Glycyrrhiza glabra[Fabaceae; Radix Glycyrrhizae], 10g | *：Administer as needed (take when joint pain is present, stop when pain is relieved) |
| Effects of Wenyang Bushen Method on the Levels of Serum 25 Hydroxyvitamin D3 in Patients with Plateau Rheumatoid Arthritis[60] | 2019 | MTX(10mg qw)+CMC(bid) | MTX(10mg qw) | Rehmannia glutinosa (Gaertn.) DC. [Orobanchaceae;Radix Rehmanniae Preparata], 30g; Angelica dahurica (Hoffm.) Benth. & Hook.f. ex Franch. & Sav. [Apiaceae;Radix Angelicae Pubescentis], 15g; Paeonia lactiflora Pall.[Paeoniaceae;Radix Paeoniae Alba], 15g; Angelica sinensis (Oliv.) Diels[Apiaceae;Radix Angelicae Sinensis], 12g; Neolitsea cassia[Lauraceae; Cortex Cinnamomi], 10g; Deerhorn glue[Cervidae;Colla Corni Cervi], 10g; Sinapis alba L. [Brassicaceae;Semen sinapis.], 10g; Ephedra sinica Stapf [Ephedraceae;Herba Ephedrae], 10g; Glycyrrhiza glabra[Fabaceae; Radix Glycyrrhizae], 9g; Poria[Polyporaceae; Sclerotium Poriae Cocos], 9g |  |
| Research on the Clinical Effect of Integrated Chinese and Western Medicinesin Treatment of 47 Cases of Rheumatoid Arthritis[61] | 2019 | MTX(10mg qw)+LEF(20mg qd)+Yiyiren Decoction(bid) | MTX(10mg qw)+LEF(20mg qd) | Coix lacryma-jobi L.[Poaceae;Semen Coicis], 30g; Angelica sinensis (Oliv.) Diels[Apiaceae;Radix Angelicae Sinensis], 12g; Saposhnikovia divaricata (Turcz. ex Ledeb.) Schischk. [Apiaceae;Radix Saposhnikoviae], 10g; Conioselinum anthriscoides ‘Chuanxiong’[Apiaceae;Rhizoma Ligustici], 10g; Ephedra sinica Stapf [Ephedraceae;Herba Ephedrae], 10g; Atractylodes lancea (Thunb.) DC.[Asteraceae;Rhizoma Atractylodis],10g; Glycyrrhiza glabra[Fabaceae; Radix Glycyrrhizae], 10g; Hansenia weberbaueriana (Fedde ex H.Wolff) Pimenov & Kljuykov [Apiaceae;Rhizoma et Radix Notopterygii], 5g; Angelica dahurica (Hoffm.) Benth. & Hook.f. ex Franch. & Sav. [Apiaceae;Radix Angelicae Pubescentis], 5g; Neolitsea cassia (L.) Kosterm.[Lauraceae;Ramulus Cinnamomi], 5g |  |
| Self-designed tongluo powder combined with Leflumide in the treatment of wind-cold and rheumatism type rheumatoid arthritis and its effect on serum WNT-3 and BMP-2 levels[62] | 2019 | LEF(20mg qd)+Celebrex(200mg qd)+Self-designed Tongluo Powder*(bid) | LEF(20mg qd)+Celebrex(200mg qd) | Astragalus mongholicus Bunge[Fabaceae;Radix Astragali seu Hedysari], 15g; Conioselinum anthriscoides ‘Chuanxiong’[Apiaceae;Rhizoma Ligustici], 15g; Aconitum carmichaeli[Ranunculaceae; Radix Aconiti Lateralis Preparata], 15g; Zaocys dhumnades(Cantor)[Natricinae;Zaocys dhumnades], 15g; Paeonia lactiflora Pall.[Paeoniaceae;Radix Paeoniae Alba], 10g; Zingiber officinale Roscoe [Zingiberaceae; Rhizoma Zingiberis], 10g; Neolitsea cassia (L.) Kosterm.[Lauraceae;Ramulus Cinnamomi], 10g; Aconitum carmichaeli Debeaux [Ranunculaceae;Radix Aconiti], 10g; Atractylodes macrocephala Koidz.[Asteraceae;Rhizoma Atractylodis Macrocephalae], 10g; Angelica sinensis (Oliv.) Diels[Apiaceae;Radix Angelicae Sinensis], 10g; Glycyrrhiza glabra[Fabaceae; Radix Glycyrrhizae], 6g; Ephedra sinica Stapf [Ephedraceae;Herba Ephedrae], 9g; Asarum sieboldii[Aristolochiaceae；Herba cum Radix Asari], 5g; Centipede[Scolopendridae; Scolopendra], 1 piece; Buthus martensii Karsch[Scorpiones;Scorpio], 1 piece; Spatholobus suberectus Dunn[Fabaceae;Caulis Spatholobi], 30g |  |
| Clinical Efficacy of Modified Duhuo Jisheng Decoction Combined with Methotrexate in Treatment of Patients with Rheumatoid Arthritis of Active Stage[63] | 2020 | MTX(10mg qw)+Duhuo Jisheng Decoction(bid) | MTX(10mg qw) | Angelica dahurica (Hoffm.) Benth. & Hook.f. ex Franch. & Sav. [Apiaceae;Radix Angelicae Pubescentis], 9g; Taxillus chinensis (DC.) Danser[Loranthaceae; Herba Taxilli], 9g; Eucommia ulmoides Oliv. [Eucommiaceae; Cortex Eucommiae], 9g; Achyranthes bidentata Blume [Amaranthaceae;Radix Achyranthis Bidentatae], 9g; Gentiana macrophylla Pall.[Gentianaceae;Radix Gentianae Macrophyllae], 9g; Neolitsea cassia[Lauraceae; Cortex Cinnamomi], 9g; Saposhnikovia divaricata (Turcz. ex Ledeb.) Schischk. [Apiaceae;Radix Saposhnikoviae], 9g; Conioselinum anthriscoides ‘Chuanxiong’[Apiaceae;Rhizoma Ligustici], 9g; Angelica sinensis (Oliv.) Diels[Apiaceae;Radix Angelicae Sinensis], 9g; Paeonia lactiflora Pall.[Paeoniaceae;Radix Paeoniae Alba], 9g; Asarum sieboldii[Aristolochiaceae；Herba cum Radix Asari], 5g; Rehmannia glutinosa[Orobanchaceae; Radix Rehmanniae], 9g; Hansenia weberbaueriana (Fedde ex H.Wolff) Pimenov & Kljuykov [Apiaceae;Rhizoma et Radix Notopterygii], 9g; Neolitsea cassia (L.) Kosterm.[Lauraceae;Ramulus Cinnamomi], 9g |  |
| Effect of Huatan Huoxue Formula on swollen joint count index and joint pain indexin patients with phlegm and blood stasis type of rheumatoid arthritis[64] | 2020 | MTX(15mg qw)+*Diclofenac sodium double release Intestine-sol Capsule(75mg qd)+SSZ(0.25g tid)+Huatan Huoxue Formula(bid) | MTX(15mg qw)+*Diclofenac sodium double release Intestine-sol Capsule(75mg qd)+SSZ(0.25g tid) | Cremastra appendiculata (D.Don) Makino[Orchidaceae;Pseudobulbus Cremastrae seu Pleiones], 10g; Sinapis alba L. [Brassicaceae;Semen sinapis.], 10g; Bombyx mori Linnaeus[Bombyx Linnaeus;Bombyx Batryticatus], 10g; Pheretima asiatica Michaelsen[Megascolecidae;Lumbricus], 10g; Arisaema erubescens[Araceae; Arisaemae cum Bile], 15g | *：Discontinue when joint pain is relieved |
| Clinical Study on the Treatment of Rheumatoid Arthritis by Removing Phlegm and Removing Blood Stasis[65] | 2020 | MTX(7.5mg qw)+SSZ*(1.0g qd)+Diclofenac Sodium Enteric-coated Tablets (50mg tid)+CMC(bid) | MTX(7.5mg qw)+SSZ*(1.0g qd)+Diclofenac Sodium Enteric-coated Tablets(50mg tid) | Dioscorea nipponica Makino[Dioscoreaceae;Rhizoma Dioscoreae Nipponicae], 20g; Sinomenium acutum[Menispermaceae; Caulis Sinomenii], 30g; Sinapis alba L. [Brassicaceae;Semen sinapis.], 10g; Paeonia lactiflora Pall. [Paeoniaceae;Radix Paeoniae Rubra], 10g; Bolbostemma paniculatum (Maxim.) Franquet [Cucurbitaceae;Rhizoma Bolbostematis ], 8g; Curcuma kwangsiensis S.G.Lee & C.F.Liang [Zingiberaceae; Rhizoma Curcumae], 6g | *：SSZ refers to salazosulfapyridine, 1.0g once a day for one week orally, then increase 0.25g daily until the dosage reaches 2-3g a day. |

**References:**

1. Li SW: **Clinical observation on treatment of Rheumatoid arthritis by Sanbi Decoction**. *Chinese Archives of Traditional Chinese Medicine* 2006:1738-1739.

2. Huang GD, Li JB, Huang DF, Huang YH, Xiao MZ, Tang LJ, Zhang SX: **Observation on effect of Centipede Longsnake Decoction combined with Western medicine in treating rheumatoid arthritis**. *Chinese Journal of Clinical Rehabilitation* 2006:186-187.

3. Wang SM, Wang X, Gong Q: **Observation of 40 cases of rheumatoid arthritis treated by combination of Traditional Chinese and Western medicine**. *Journal of Sichuan of Traditional Chinese Medicine* 2006:47-48.

4. Liu B, Liu W, Wang Y: **Siwu Decoction combined with methotrexate attenuated and effective treatment for rheumatoid arthritis**. *Chinese Journal of Integrative Medicine* 2007, **27**.

5. Luo YH: **32 cases of senile rheumatoid arthritis were treated with Jianpi Yiqi Huoxue Decoction**. *Shaanxi J Tradit Chin Med* 2008:997-999.

6. Shen YP, Chuan XW, Liu Y: **Effect of Ziyin YiQi Decoction on hormone withdrawal in rheumatoid arthritis**. *Shandong Journal of Traditional Chinese Medicine* 2008:95-96.

7. Xiang CC, Xiong QD, Wu JY: **40 cases of rheumatoid arthritis were treated with Bushen Decoction and Western medicine**. *Shaanxi J Tradit Chin Med* 2009, **30**:1614-1616.

8. Ma WK, Zhong Q, Liu ZQ, Yao XM: **Clinical study on the treatment of rheumatoid arthritis by Sanwu capsule combined with methotrexate and salazosulphapyridine**. *Journal of New Chinese Medicine* 2009, **41**:42-44.

9. Li AM: **Siteng Yin and Siwu Tang were used to treat 160 cases of rheumatoid arthritis**. *Chinese ethnic and folk medicine* 2009, **18**:51-52.

10. Liu XD, Zhang JL, Ye LH, Liu FY, Chen Y: **Effect of Wenhua Juanbi Decoction on TNF and IL-1 in peripheral blood of patients with rheumatoid arthritis**. *Chinese Journal of Integrative Medicine* 2009, **29**:787-790.

11. Chen ZW, Sun J, Li YM, Chen YQ: **[Efficacy of Shenshi Qianghuo Dihuang Decoction in rheumatoid arthritis: a randomized controlled trial]**. *Zhong Xi Yi Jie He Xue Bao* 2010, **8**:35-39.

12. Yao JH: **Clinical observation of 58 cases of rheumatoid arthritis treated by Rebi Decoction**. *Journal of Traditional Chinese Medicine* 2010, **51**:1086-1088.

13. Zhou CY, Tang JY, Fang DY, Pan Z, Ma F: **A clinical study on Simiao Xiaobi Decoction in the treatment of rheumatoid arthritis in active stage**. *Chinese Journal of Integrative Medicine* 2010, **30**:275-279.

14. Shen HB, Bai YJ, Huo ZJ, Li WN, Tang XP: **Assessment of clinical effect of therapy combining disease with syndrome on rheumatoid arthritis**. *Journal of Traditional Chinese Medicine* 2011, **31**:39-43.

15. Han SL, Song YW: **A randomized controlled study of Huatan Quyu Juanbi Decoction in the treatment of rheumatoid arthritis**. *Chinese Archives of Traditional Chinese Medicine* 2011, **29**:2808-2810.

16. Yang B, Liang QH, Wu D, Tang T, Peng WJ: **Clinical observation of Simiao Pill combined with Western medicine in treating 20 cases of active rheumatoid arthritis**. *Journal of Traditional Chinese Medicine* 2011, **52**:1566-1569.

17. Li ZL, Wang RS, Shen J, Xu LM, Yue T, Zhu Q, Jiang T, He DY: **Effect of tonifying kidney and removing blood stasis on bone metabolism in patients with early rheumatoid arthritis**. *Journal of Traditional Chinese Medicine* 2012, **53**:215-218.

18. Liu EC, Liu Y, Wang SH, Wang SF: **Clinical observation on the treatment of rheumatoid arthritis with Yaotongning Capsule**. *Clinical Journal of Traditional Chinese Medicine* 2013, **25**:109-111.

19. Wei Y, Yang XM, Wang SE: **Clinical observation of 50 cases of active rheumatoid arthritis treated by combination of Chinese and Western medicine**. *Science and Technology of Chinese Traditional Medicine* 2013, **20**:54-55.

20. Li JP: **Clinical analysis of 40 cases of rheumatoid arthritis treated by combination of Chinese and Western medicine**. *Journal of Sichuan of Traditional Chinese Medicine* 2013, **31**:86-88.

21. Wei W, Liu KK: **Clinical Observation of Traditional Chinese Medicine Combined with Etanercept in Treatment of Elderly Rheumatoid Arthritis**. *Chinese Archives of Traditional Chinese Medicine* 2013, **31**:939-941.

22. Yang YJ: **Treatment of 42 cases of rheumatoid arthritis with Compound Maqianzi Powder**. *China pharmaceutical* 2013, **22**:84-85.

23. Wang Z, Tao XJ: **[Treatment of rheumatoid arthritis by Yangxue Tongluo Recipe combined with immunosuppressive agents: a clinical observation]**. *Zhongguo Zhong Xi Yi Jie He Za Zhi* 2014, **34**:276-278.

24. He DC, Xiao JJ: **Clinical efficacy and safety of Bizhengning in the treatment of rheumatoid arthritis**. *Modern Journal of Integrated Traditional Chinese and Western Medicine* 2014, **23**:3090-3092.

25. Zhang HJ, Chen TB: **Clinical observation of Duhuo Qushi Zhitong Decoction combined with meloxicam in treatment of rheumatoid arthritis**. *Shaanxi J Tradit Chin Med* 2014:1336-1338.

26. Niu JH: **Observation on the curative effect of Guizhi Shaoyao Zhimu Decoction combined with Juanbi Decoction in treating rheumatoid arthritis**. *Shaanxi J Tradit Chin Med* 2014, **35**:984-986.

27. Cao YH, Liu J: **Clinical observation of heat clearing and wetting combined with Western medicine in the treatment of dampness-heat type rheumatoid arthritis**. *Journal of Anhui University of Chinese Medicine* 2014, **33**:19-22.

28. Yu M, Chen YY: **Observation on effect of integrated Chinese medicine and western medicine on 32 pafients with rheumatoid arthritis**. *Internal Medicine of China* 2014, **9**:12-14.

29. Su SZ, Ye XY, Peng JH, Chen B: **Clinical observation on treating rheumatoid arthritis by Bushen Huoxue therapy**. *Clinical Journal of Chinese Medicine* 2015:18-20.

30. Shu C, Hua DP, Li Y: **Clinical Observation on Yishen Qingluo Huoxue Decoction in Treatment of Intermingled Phlegm and Blood Stasis Type of Rheumatoid Arthritis**. *Chinese Archives of Traditional Chinese Medicine* 2015, **33**:34-37.

31. Li Y, Fan WM, Gu SF, Liu WH: **Clinical observation of Yishen Qingluo Huoxue Decoction in treating rheumatoid arthritis with Mutual junction of phlegm and blood stasis**. *Journal of Anhui University of Chinese Medicine* 2015, **34**:17-20.

32. Zhao FC: **Observation on the curative effect of combined Chinese and Western medicine on rheumatoid arthritis**. *Modern Journal of Integrated Traditional Chinese and Western Medicine* 2015, **24**:2107-2109.

33. Li X, Liu JF, Zhao GQ: **Clinical observation on the treatment of rheumatoid arthritis by adding or subtracting Judanxi gout prescription combined with methotrexate**. *Anhui Medical Journal* 2015, **36**:995-998.

34. Zheng XB, Shi CH: **Zhuithmg Tougu Capsules combined with Methotmxate on the Treatment of Rheumatoid Arthritis for 80 Cases**. *Chinese medicine modern distance education* 2015, **13**:63-65.

35. Qian X, Chen X, Wei G, Guo YK, Sun ZL: **Clinical Effect of Duhuo Jisheng Decoction Combined with Meloxicam in Treatment of Rheumatoid Arthritis**. *Chinese Journal of Experimental Traditional Medical Formulae* 2016, **22**:173-176.

36. Jia FY, Wang J, Yang QB: **A clinical study on treatment of rheumatoid arthritis by eliminating rheum bnourishing the liver and yishen decoction**. *Asia-Pacific Traditional Medicine* 2016, **12**:143-144.

37. You BR, Tian XW, Liu CJ: **Clinical observation of Liuwei Dihuang combined with Siwu Decoction combined with methotrexate in the treatment of rheumatoid arthritis**. *Guiding Journal of TCM* 2016, **22**:95-97.

38. Han L, Ba Y, Gu JNTH, Shi R, Ba HEGL, Wei R: **Effect of removing wind, dehumidifying and tonifying kidney in the treatment of senile rheumatoid arthritis and the influence of antibody levels against cyclic citrulline polypeptide, rheumatoid factor, erythrocyte sedimentation rate, C-reactive protein and keratin**. *Chinese Journal of Gerontology* 2016, **36**:4558-4560.

39. Liu Y, Zhang HJ, Guo YX, Meng QL: **Clinical Observation of 35 Cases of Ｒheumatoid Arthritis of Damp Heat Type Treated with Simiao Podwer combined with Xuanbi Decoction and Western Medicine**. *World Journal of Integrated Traditional and Western Medicine* 2016, **11**:800-803.

40. Chen F, Min CH, Zhou Y, Zhang HY: **Clinical observation of 35 cases of Rheumatoid arthritis of Kidney Qi deficiency and Cold type treated by Yishen Juanbi Capsule combined with Western medicine**. *Journal of Traditional Chinese Medicine* 2016, **57**:1045-1048.

41. Jiang D, Wu J: **Analysis and evaluation of the curative effect of Self-prepared Chinese medicine combined with anti-rheumatism medicine on active rheumatoid arthritis**. *J Mod Med Health* 2016, **32**:3507-3510.

42. Wang HT: **Clinical research on treating rheumatoid arthritis by the Buyi Qixue, Qushi Tongluo therapy**. *Clinical Journal of Chinese Medicine* 2017, **9**:62-63.

43. Pang AM, Jiang P, Li JX, Chi XW: **Effects of Hebi Formula on bone erosion in early rheumatoid arthritis of liver and spleen disorder type**. *Chinese Journal of Traditional Chinese Medicine* 2017, **32**:5682-5685.

44. Li J, Rong B, Jia J, Pan MZ: **Effect of Modified Juanbi Decoction on Inflammatory Factors and Serum MMP-3，OPG and ＲANKL in Synovial Fluid of Patients with Wind-cold-wetness Type Ｒheumatoid Arthritis**. *Chinese Journal of Experimental Traditional Medical Formulae* 2017, **23**:165-170.

45. Gao D, Bu SS: **Clinical Research on Liuwei Dihuang Decoction combined with Siwu Decoction Treatment of Rheumatoid Arthritis**. *ACTA Chinese Medicine* 2017, **32**:1079-1081.

46. Guo HM, Guo LW, Xing JY: **Clinical observation on 340 cases of Rheumatoid arthritis treated by Mayi Tongbi Capsule**. *World Chinese Medicine* 2017, **12**:1859-1862.

47. Wang Y, Tu SH: **Clinical Study on Jiawei Wutou Decoction in Treatment of Rheumatoid Arthritis**. *ACTA Chinese Medicine* 2017, **32**:1716-1719.

48. Ma ZL, Wang CY, Liu BB: **Clinical observation of Buyang Huanwu Decoction in treating phlegm and blood stasis type rheumatoid arthritis**. *Modern Journal of Integrated Traditional Chinese and Western Medicine* 2018, **27**:1313-1315.

49. Guo HL, Wang G, Tian JX: **Gulao Yukang Pills combined with methotrexate tablets in the treatment of 40 cases of rheumatoid arthritis**. *TCM Res* 2018, **31**:23-26.

50. Xu GS, Yu XF, Kong MZ, Chen JC, Qiu MS: **Clinical observation on the treatment of rheumatoid arthritis with heat and blood stasis by traditional Chinese medicine therapy of clearing heat and activating blood circulation**. *Chinese Journal of Traditional Chinese Medicine* 2018, **33**:1167-1170.

51. Bian ZQ, Wang YD: **Wenyang Chushi, Huatan tongluo prescription combined with conventional therapy to treat 90 cases of cold and dampness bizu rheumatoid arthritis**. *Global Chinese Medicine* 2018, **11**:447-450.

52. Shi L, Yang F: **40 cases of rheumatoid arthritis were treated by renal differentiation**. *Journal of Shaanxi University of Traditional Chinese Medicine* 2018, **041**:66-69.

53. Ge S: **Observation on the curative effect of combined Chinese and Western medicine on rheumatoid arthritis**. *Journal of Practical Traditional Chinese Medicine* 2018, **34**:1371-1372.

54. Zeng JY, Chen SK: **The clinical effect of traditional Chinese medicine combined with antirheumatic drugs on rheumatoid arthritis**. *Clinical Journal of Chinese Medicine* 2018, **10**:82-83.

55. Hu H: **Short-term and Long-term Effects of Applying Traditional Chinese Medicine Syndrome Differentiation Combined with Methotrexate in the Treatment of Ｒheumatoid Arthritis**. *Journal of Sichuan of Traditional Chinese Medicine* 2018, **36**:76-79.

56. Yuan L, Wu JY, Tang J, Chen YG, Zhang ZY: **Clinical Observation of Baihu Plus Guizhi Decoction Combined with Western Medicine**

**in Treating Rheumatoid Arthritis with Rheumatic Heat Arthralgia Syndrome**. *Journal of Liaoning university of TCM* 2019, **21**:168-171.

57. Pang J, Xu J, Li L, LI YL: **Clinical study of Guikun Fengshi Mixture combined with methotrexate in the treatment of rheumatoid arthritis**. *Pharmacol Clin Chin Mater Med* 2019, **35**:155-158.

58. Zhao L: **Clinical efficacy of the Guizhi Shaoyao Zhimu decoction on rheumatoid arthritis of the Fenghan Shibi type**. *Clinical Journal of Chinese Medicine* 2019, **11**:51-53.

59. Li JH, Lin YB, Yu GS, Zhang SX, Liu YY, Xu HB: **Clinical Observation on Treating 41 Cases of Rheumatoid Arthritis of Mixed Heat and Cold Type with Guizhi Shaoyao Zhimu Tang Combined with Methotrexate**. *Rheumatism and Arthritis* 2019, **8**:31-34.

60. Song WH, Li Q, Wang FZ, Tao WX, Guan QCR: **Effects of Wenyang Bushen Method on the Levels of Serum 25 Hydroxyvitamin D3 in Patients with Plateau Rheumatoid Arthritis**. *World Chinese Medicine* 2019, **14**:1466-1470.

61. Zhang KL: **Research on the Clinical Effect of Integrated Chinese and Western Medicinesin Treatment of 47 Cases of Rheumatoid Arthritis**. *Chinese ethnic and folk medicine* 2019, **28**:83-85.

62. Fang XG, Wang M, Chen HY, Bai ZX, Chen ZF, Lan PM: **Self-designed tongluo powder combined with Leflumide in the treatment of wind-cold and rheumatism type rheumatoid arthritis and its effect on serum WNT-3 and BMP-2 levels**. *Modern Journal of Integrated Traditional Chinese and Western Medicine* 2019, **28**:1408-1411.

63. Cao MZ, Wang ZL, Jiang XS: **Clinical Efficacy of Modified Duhuo Jisheng Decoction Combined with Methotrexate in Treatment of Patients with Rheumatoid Arthritis of Active Stage**. *China Journal of Pharmaceutical Economics* 2020, **15**:55-58.

64. Li D: **Effect of Huatan Huoxue Formula on swollen joint count index and joint pain indexin patients with phlegm and blood stasis type of rheumatoid arthritis**. *J Medical Forum* 2020, **41**:91-94.

65. Jiang P, Ma LJ: **Clinical Study on the Treatment of Rheumatoid Arthritis by Removing Phlegm and Removing Blood Stasis**. *China Continuing Medical Education* 2020, **12**:158-160.
